# Photosystem Perturbation by *Staygreen* Mutations Confers Allele‐Dependent Defences Against Infections of Pathogens With Different Lifestyles and Abiotic Stress Tolerance

**DOI:** 10.1111/pce.70229

**Published:** 2025-10-13

**Authors:** Junyi Tan, Zhejuan Tian, Feifan Chen, Kang Gao, Jinghao Jin, Anthony P. Keinath, Ronald D. Dymerski, Zhiming Wu, Yiqun Weng

**Affiliations:** ^1^ Department of Plant and Agroecosystem Sciences University of Wisconsin‐Madison Madison Wisconsin USA; ^2^ Institute of Cash Crops Hebei Academy of Agriculture & Forestry Sciences Shijiazhuang China; ^3^ College of Plant Protection Yangzhou University Yangzhou China; ^4^ Department of Plant and Environmental Sciences Clemson University Coastal Research and Education Center Charleston South Carolina USA; ^5^ USDA‐ARS Vegetable Crops Research Unit Madison Wisconsin USA

**Keywords:** abiotic stress tolerance, chlorophyll degradation, cucumber, multiple disease resistance, ROS, senescence, staygreen

## Abstract

The *staygreen* (*SGR*) gene encodes the magnesium dechelatase that plays an important regulatory role during chlorophyll degradation. Our previous work revealed a nonsynonymous SNP (*A323G*) inside cucumber *CsSGR* that is responsible for multiple disease resistance (MDR), but the underlying mechanism is unknown. Here we report the development, phenotypic, genetic, or transcriptomic characterisation of near‐isogenic lines for the *A323G* locus and knock‐out mutants of *CsSGR* (*SGRΔ37* with 37‐bp deletion) in response to biotic/abiotic stresses. Both SNP and *SGRΔ37* mutants show enhanced MDR against infection of five pathogens with different lifestyles, as well as low‐temperature tolerance than the wildtype, and *SGRΔ37* is a stronger allele with higher resistance/tolerance than the *A323G* allele. Physical interactions of CsSGR with itself and other chlorophyll catabolic enzymes (CCEs), light‐harvesting chlorophyll a/b‐binding1 proteins (LHCB1s), and the chlorophyll homoeostasis regulator CsBCM are significantly reduced or abolished in *A323G* and *SGRΔ37* mutants, respectively. Comparative transcriptome analyses revealed a complex regulatory network in which both passive and active defences contribute to *Cssgr*‐conferred MDR. The loss‐of‐susceptibility *CsSGR* mutations downregulate expression of chlorophyll catabolic genes, slow down chlorophyll degradation, and delay pathogenesis‐induced senescence, thus providing passive defence. The active defence involves SA and/or JA biosynthesis/signalling pathways, which are likely triggered by ROS‐mediated retrograde signalling due to perturbation of the photosynthetic electron transport chain. We propose that *CsSGR* is a target of choice for gene editing to develop mutant alleles for enhanced MDR. Further, mutations of genes involving chlorophyll metabolism, photosystems, or chloroplast development could be a potential source of MDR for plant breeding.

## Summary

• *CsSGR* mutations confer allele‐dependent multi‐disease resistance and chilling tolerance in cucumber (*Cucumis sativus* L.).
• The resistance strength correlates with the degree of disruption of SGR's protein interactions and functions through both passive and active defence pathways.

## 1 Introduction

In plants, the ‘staygreen’ (SGR) trait refers to the heritable delayed leaf senescence in relation to a regular, reference phenotype with green foliage. A plant with the staygreen phenotype may maintain active photosynthetic capacity in the critical yield‐formation stage, which thus increasing crop yield or yield stability (Sjödin 1971; Thomas and Smart 1993; Thomas and Ougham 2014). The genetic basis of the staygreen phenotype is largely quantitative in nature; hundreds of staygreen QTL have been identified in many crops, and a few have been cloned (reviewed in Kamal et al. 2019). Loss of chlorophyll (Chl, see Table S1 for list of abbreviations) is the most visible hallmark of leaf senescence. Conceivably, the staygreen phenotype may reflect impaired or delayed Chl catabolism. Indeed, mutations in Chl catabolic genes (*CCGs*) (see Figure S1) often result in staygreen with those of the *staygreen* gene (*SGR*) being the most frequently associated with this mutant phenotype, which has been reported in many plants like *Arabidopsis* (Ren et al. 2007), rice (Cha et al. 2002; Jiang et al. 2007; Park et al. 2007; Zhao et al. 2019; Shin et al. 2020), green pea (Armstead et al. 2007; Sato et al. 2007), soybean (Fang et al. 2014), or tomato (Barry et al. 2008; Hu et al. 2011; Thomason& Ougham, 2014; Chen et al. 2016; Yu et al. 2021). The Arabidopsis *staygreen* gene *AtSGR1/NYE1* encodes the Mg‐dechelatase that removes Mg ion and converts Chl a to pheophytin a during Chl degradation (Shimoda et al. 2016). In higher plants, the conserved SGR proteins could be classified into two groups: SGR and SGRL (SGR‐like) with most plants having one (e.g., cucumber CsSGR) or two SGR (e.g., AtSGR1 and AtSGR2 in Arabidopsis), and one SGRL (Barry et al. 2008; Sakuraba et al. 2014). A typical SGR protein contains three domains: a chloroplast transit peptide domain, a conserved SGR central core domain, and a variable C‐terminal domain (Hörtensteiner 2006; Sakuraba et al. 2015). The Arabidopsis C‐terminal domain contains a conserved cysteine‐rich motif (CRM) with the cysteine residues being essential for AtSGR1 function (Xie et al. 2019).

SGR plays a key role in the regulation of Chl degradation during development‐ or stress‐induced leaf senescence, fruit ripening, or seed development. In Arabidopsis, the *SGR*‐associated gene regulatory network during Chl degradation and senescence involves various phytohormone signalling pathways (e.g., JA, SA, ABA, and ET), which is also influenced by many internal (e.g., lineages, organs, or developmental stages) or external (e.g., light, and temperature) factors (Hörtensteiner 2006; Kuai et al. 2018; Woo et al. 2019; Guo et al. 2021; Tanaka and Ito 2024). In general, the *SGR* expression level is positively correlated with the speed of Chl degradation, which is regulated by many transcription factors (TF). For example, Arabidopsis MYC2/3/4, EIN3, ABI5, PIF4, and NAC016/019/046/055/072 positively regulate *AtSGR1* expression during leaf senescence (Sakuraba et al. 2012, 2014; Oda‐Yamamizo et al. 2016; Yuan et al. 2019). ABI3 upregulates *AtSGR1/AtSGR2* expression during seed maturation (Delmas et al. 2013; Li et al. 2017). The tomato NOR‐like1 (a NAC TF) increases *SlSGR1* expression and Chl degradation during fruit ripening (Gao et al. 2018). The litchi (*Litchi chinensis*) LcNAC002 binds to *LcSGR* to coregulate Chl degradation and anthocyanin biosynthesis (Zou et al. 2023). Chl degradation involves physical interactions among CCEs and between CCEs and LHCII proteins (e.g., LHCBs) (Park et al. 2007; Sakuraba et al. 2012, 2014).

Some *SGR* mutants seem to be practical value in crop improvement. For example, variants or differential expression of the *SGR* gene may improve the digestibility of forage grasses (Zhou et al. 2011; Xu et al. 2019), broccoli nutrition quality (Li, Hussain, et al. 2024), pigment content (lycopene, carotenoids) and postharvest shelf life in tomato (Barry et al. 2008; Hu et al. 2011; Luo et al. 2013; Kim et al. 2023, 2024; Cui et al. 2024), and oil accumulation in the rapeseed (Qian et al. 2016). In rice, *OsSGR* was suggested to be under selection for maturity between the *indica* and *Japonica* cultivation groups, and the *indica* allele could be explored to improve the yield of *Japonica* rice (Shin et al. 2020). There are also reports linking SGR functions with abiotic stress responses such as cold tolerance in cucumber (Dong et al. 2023) or heat tolerance in perennial ryegrass (Zhang et al. 2024). *SGR* has been reported to be associated with disease symptom development. Mur et al. (2010) found that overexpression of *AtSGR1* accelerated HR‐associated cell death caused by the accumulation of phototoxic Chl catabolites and ROS. Pathogen infection‐induced *AtSGR1* expression is required for the development of disease symptoms (chlorosis or necrosis) caused by bacterial or fungal pathogens (Mecey et al. 2011). Similarly, the *sgr* mutant of *Medicago truncatula* and alfalfa *SGR*‐RNAi lines show enhanced defence responses to the infection by the non‐host Asian soybean rust or anthracnose pathogens, suggesting a possible role of *SGR* in non‐host resistance (Ishiga et al. 2015). In soybean, *GmSGR1* and *GmSGR2* are candidates for two anti‐chlorosis resistance QTL against the sudden death syndrome (Chang et al. 2019). A rice *OsSGR* mutant confers enhanced resistance to rice sheath blight through elevating cytokinin content (Xie et al. 2024).

We previously found that a SNP (*A323G*) inside the cucumber *CsSGR* gene was responsible for multiple disease resistance (MDR) against the downy mildew (DM, *dm1* locus), angular leaf spot (ALS, *psl* locus) and anthracnose (AR, *cla* locus) (Y. Wang et al. 2019) caused by the biotrophic oomycete *Pseudoperonospora cubensis* (*Pcu*), the hemi‐biotrophic bacterial *Pseudomonas syringae pv. lachrymans* (*Psl*), and the fungal *Colletotrichum orbiculare (Cor)*, respectively. This loss‐of‐susceptibility mutation (*Cssgr*) provided effective protection of cucumbers in the field against pathogens for over 50 years. However, the molecular mechanism of *Cssgr*‐regulated MDR is unknown. In this study, we developed NILs and knock‐out (KO) mutants for *CsSGR* with CRISPR‐Cas9‐based gene editing. From extensive phenotypic, transcriptomic, and molecular investigations on these lines, we show that (1) NILs and *SGR* KO mutants exhibited enhanced resistance against five diseases caused by pathogens with different lifestyles, as well as chilling tolerance. (2) *Cssgr* mutations can reduce pathogen‐induced *CsSGR* expression and CHL degradation, delay leaf senescence, and maintain high photosynthesis capacity and robust growth, thus mitigating disease damage, which provides passive defence in resistant lines. (3) *Cssgr* mutations result in extensive transcriptome reprogramming, which activates defence responses for enhanced and active defence against pathogen infections. (4) The mutated CsSGR protein impacts interactions with itself and other key players in its regulatory network and the consequences seem to be allele dependent. (5) Thus, the MDR could be enhanced with novel, strong *Cssgr* mutant alleles through gene editing.

## 2 Materials and Methods

### 2.1 Plant Materials, Phenotyping, and QTL Analysis

The resistant (NIL‐R) and susceptible (NIL‐S) NILs for the *dm1/psl/cla* MDR locus were developed with a marker‐assisted backcrossing scheme (Figure S2). Resistances to DM, AR, ALS, TLS, and PM were evaluated in replicated trials (RCBD design) of different environments (growth chambers, greenhouses or fields) following methods described early (He et al. 2013; Pan et al. 2018; Y. Wang et al. 2019) with appropriate resistant and susceptible controls. In growth chamber tests, disease symptoms were recorded at 0, 3 and 5 days postinoculation (dpi). DM and ALS resistance was also evaluated in field or greenhouse trials. DM responses were assessed with three rating criteria, including yellowing (Yel), necrosis (Nec), and general impression (GI) (Tan et al. 2022). For low‐temperature (LT) stress treatment, 3‐week‐old seedlings of different genotypes were exposed to 13/10°C (day/night) for 2 weeks, and the chilling damage was quantified with six ratings (0, 1, 3, 5, 7, 9) (Dong et al. 2023).

QTL analysis of PM resistance was performed using 109 RILs derived from the Gy14 × 9930 cross (Weng et al. 2015) with phenotypic data (scores of yellowing/chlorosis) collected from a greenhouse trial under natural infection. QTL analysis was performed with the *R/qtl* package following Tan et al. (2022). The association of PM‐induced chlorosis with the *SGR* locus was validated with an F_2_ population derived from the cross between NIL‐R and NIL‐S in which plants were scored with either ‘yellowing’ or ‘stay‐green’, and the segregation was tested with the χ^2^ test. CHL content was measured from each plant. These F_2_ plants were further genotyped with the causal *A323G* SNP. Single marker analysis was performed to test the association of *SGR* alleles with CHL contents.

### 2.2 Physiological Measurements

CHL extraction and quantitation in NILs followed Ni et al. (2009). The maximum quantum efficiency of PSII (Fv/Fm) of pathogen‐infected or mock leaves was determined using the LI‐6400‐40 portable Leaf Chamber Fluorometer with three biological replicates per line per treatment. Hydrogen peroxide (H_2_O_2_) accumulation was visualised with DAB staining (Jiang et al. 2011). Quantitation of H_2_O_2_ in the first true leaves before or after pathogen infection was estimated by the Amplex Red H_2_O_2_/Peroxidase assay kit (ThermoFisher, USA). Cell death in NILs was estimated by measuring electrolyte conductivity (EC) (Hatsugai and Katagiri 2018) with a SevenCompact Cond metre S230 (Mettler‐Toledo, OH) at 3, 5 and 7 dpi with pathogens using water (Mock) as controls. There were three biological and three technical replicates for each sample.

### 2.3 CRISPR‐Ca9‐Based Gene Editing

Two pairs of oligonucleotide sequences of sgRNAs (sgRNA1 between introns 2 and 3 and sgRNA2 in exon 3) were designed in the SNP (*A323G*) region of the *CsSGR* gene using CRISPR‐P v2.0 (http://crispr.hzau.edu.cn/CRISPR2/; Information of markers, or primers used in this study is provided in Table S2). The pBSE402‐*CsSGR* vector was introduced into the *Agrobacterium tumefaciens* strain EHA105, and the transformation followed the methods described in Liu et al. (2023). The positive T_0_ plants were confirmed with *bar‐* and *Cas9*‐specific PCR primers and then self‐pollinated to produce T_1_. The mutations in T_2_ plants were further confirmed with Sanger sequencing. Phenotypic characterisation of independent gene‐editing lines was performed in the Walnut Street Greenhouses of the University of Wisconsin‐Madison.

### 2.4 Transcriptome Profiling and qRT‐PCR

RNA‐Seq was performed on leaf samples of two NILs, SGRΔ37‐2 KO, and 9930 (WT) at 0, 3 and 5 dpi with *Pcu* and *Cor* pathogens, with two biological replications for each treatment. High‐quality Illumina sequencing reads were aligned to the Gy14v2.0 reference genome (https://cucurbitgenomics.org/) using TopHat v2.1.1 (Kim et al. 2013). The reads uniquely mapped to each gene were counted with *HTSeq‐count* (Anders et al. 2015). PCA was performed with the R/DESeq. 2 package on regularised logarithm values of read counts. DEGs between pairwise comparisons were also identified with R/DESeq. 2 (Love et al. 2014) with a cutoff of adjusted *P* < *FDR*_*0.05*_. GO enrichment analysis of DEGs was performed with *R/clusterProfiler* v4.0. The ‘Simple Tidy GeneCoEx’ workflow in R was used to identify genes with co‐expression patterns (Li et al. 2023). Potential off‐target sites by gene editing were predicted with the Cas‐OFFinder web tool (http://www.rgenome.net/cas-offinder/) and verified by examining the transcriptome data at predicted off‐target sites.

For qPCR, total RNAs of NILs, KO, and relevant control lines were extracted from seedlings at 0, 3, 5 and 7 dpi with *Pcu*, *Cor*, and *Psl*, and water control (mock). qPCR procedures and data analysis followed Tan et al. (2022) with three biological and three technical replicates for each sample.

### 2.5 Subcellular Localisation and Protein‐Protein Interaction Assays

For subcellular location of SGR, the full‐length CDS of C*sSGR* (WT), *CsSGR* ^*A323G*^ (SNP), and *CsSGR* ^*Δ37*^ (37 bp deletion) were individually inserted into the modified p1307 plant transformation vector under the control of the CaMV‐35S promoter. Transient overexpression in tobacco epidermal cells followed Zhao et al. (2023). For Y2H assays, the full‐length CDS of *CsLHCB1s*, *CsSGR*, *CsSGR* ^*A323G*,^ and *CsSGR* ^*Δ37*^ were PCR amplified and fused into pGADT7 (bait vector) and pGBKT7 (prey vector), which were transformed into the yeast strain AH109. For membrane protein Y2H assays, the full‐length CDS of the *CsCCGs*, *CsSGR*, *CsSGR* ^*A323G*^, and *CsSGR* ^*Δ37*^ were PCR amplified and fused into pMetYCgate (bait vector) and pNXgate32‐3HA (prey vector), which were transformed into the yeast strain THY.AP4. In BiFC assays, full‐length CDS without the stop codon of target genes were PCR amplified and fused into pSPYNE‐35S and pSPYCE‐35S vectors, including each half of the YFP (N‐ or C‐terminus) to create the fusion protein. For LCI assays (Zhao et al. 2023), full‐length CDSs of *CsLHCB1s*, *CsCCGs*, *CsSGR*, *CsSGR* ^A323G^, and *CsSGR* ^*Δ37*^ with or without stop codons were PCR‐amplified and inserted into the pCAMBIA1300‐cLUC and pCAMBIA1300‐nLUC vectors, respectively.

### 2.6 Co‐IP Assay

For Co‐IP assay in tobacco (*Nicotiana benthamiana*), the CDS of *CsSGR*, *CsSGR* ^*A323G*^, *CsSGR* ^*Δ37*^, *CsLHCB1.1*, *CsLHCB1.3*, *CsPPH*, *CsPAO*, and *CsBCM* were cloned into modified pCambia2300 vectors to generate constructs with specific tags (GFP, RFP‐HA, or FLAG). *Agrobacterium* strains containing these constructs were collected and diluted to OD_600_ = 0.6 and then infiltrated into leaves of 4‐week‐old tobacco plants. After 48 h, total proteins were extracted using an extraction buffer containing 10 mM Tris‐HCl (pH 7.4), 150 mM NaCl, 1 mM EDTA, 5 mM DTT, 1% IGEPAL CA‐630, 1 mM EGTA, 1 mM PMSF, and the protease inhibitor cocktail (beyotime). Following centrifugation (12,000 × rpm for 10 min at 4°C), the supernatant was incubated with either Anti‐FLAG Agarose (Sigma) or GFP‐Trap Magnetic Agarose (Chromotek) for 2 h at 4°C. Beads were collected and washed five times with wash buffer (10 mM Tris‐HCl [pH 7.4], 150 mM NaCl, 0.05% IGEPAL CA‐630, 0.5 mM EDTA). Proteins eluted from the beads were then subjected to SDS‐PAGE and immunoblot analyses using anti‐FLAG (Sigma‐Aldrich), anti‐GFP, or anti‐HA antibodies (Abmart, China).

## 3 Results

### 3.1 *Cssgr*‐Conferred MDR Is Associated With Reduced *CCG* Expression, Chl Degradation, ROS Accumulation/Cell Death, and Delayed Leaf Senescence

To characterise the *Cssgr*‐conferred MDR, we developed NILs for this locus in which the resistance allele from Gy14 was introduced into the susceptible ‘9930’ background through MABC (Figure S2a). GBS of two BC_4_F_3_ lines carrying homozygous recessive (NIL‐R, *Cssgr*) and dominant (NIL‐S, *CsSGR*) alleles indicated that both lines had highly homogeneous 9930 background except for a 549‐kbp introgression harbouring the Gy14 allele in NIL‐R (Figure S2b). In multi‐year greenhouse and field trials, we collected data for FT, FL, FD, VL, and LBN in the two NILs and 9930 (Figure S2c–i). Significant differences in these traits were not found among these lines, suggesting no linkage drag of the resistance allele, and NIL‐S is largely an equivalent of 9930 both phenotypically and genetically.

**Figure 1:**
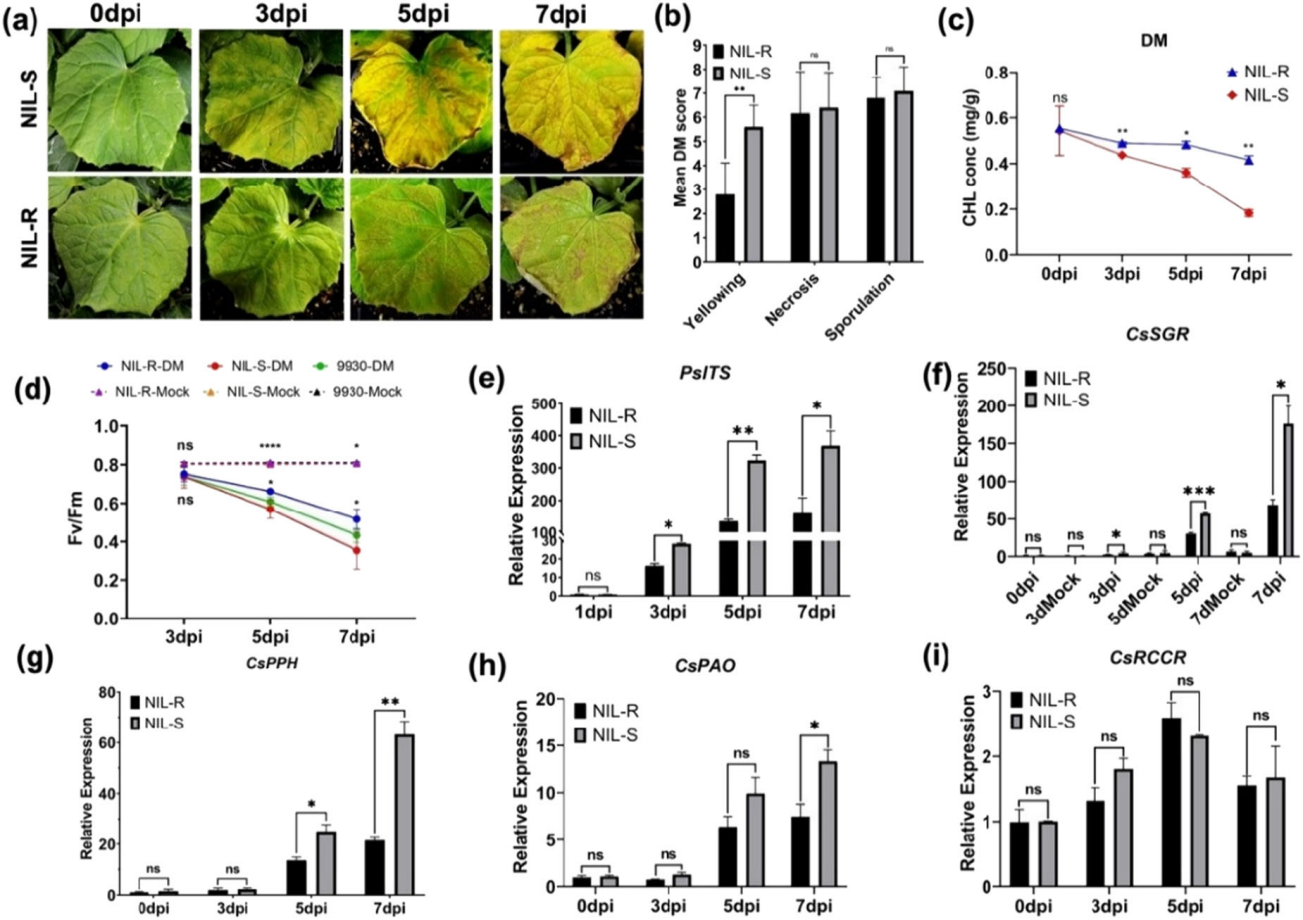
Characterisation of *A323G*‐conferred DM resistance in NILs. (a) Leaf DM symptom development on NILs at different dpi with *P. cubensis* (*Pcu*) under growth chamber conditions. (b) Bar graphs of mean DM disease scores of two NILs showing the anti‐chlorosis effect conferred by the *Cssgr* resistance allele. Upon inoculation, NIL‐R shows reduced CHL degradation (c) and less reduction in maximum quantum yield of PSII (Fv/Fm) (d) than NIL‐S. The upper symbols indicate statistical significance among different genotypes after pathogen infection or under mock treatments based on one‐way ANOVA. The lower symbols indicate statistical significance between NILs based on Student's *t*‐test. *Pcu* growth measured with the relative expression level of *PcuITS* (internal transcribed spacer) from qPCR is also significantly repressed on NIL‐R than on NIL‐S (e). Upon *Pcu* infection, the relative expression of three CHL catabolic genes, *CsSGR* (f), *CsPPH* (g), and *CsPAO* (h) is significantly upregulated in NIL‐S than in NIL‐R but no significance change in the expression in CsRCCR at any time point (i). Bars = mean ± SD (*n* = 8 for B and *n* = 3 for C–H). ns = not significant; *, and **: *p* < *0.05* and *0.01* respectively based on pair‐wise *t*‐tests.

We compared symptom development and pathogen growth on the two NILs in response to artificial inoculation of the *Pcu*, *Cor*, and *Psl* pathogens. Upon *Pcu* infection, NIL‐S displayed angular chlorotic lesions at 3 dpi, which coalesced into large chlorotic and necrotic lesions at 5 dpi. In contrast, NIL‐R showed limited‐size necrotic lesions with a lower yellowing score at 7 dpi; however, there was no difference in necrosis or sporulation scores between them (Figure 1a,b), suggesting a critical role of anti‐chlorosis in *Cssgr*‐dependent DM resistance. Accordingly, in response to *Pcu* infection, NIL‐R maintained a higher CHL content and maximum quantum efficiency of PSII (Fv/Fm) than NIL‐S; from 3 dpi, *Pcu* growth was also repressed in NIL‐R (Figure 1c–e). Similar trends were observed in the two NILs in response to *Cor* and *Psl* inoculations, except that *Cor* growth was only transiently inhibited at 3 dpi in NIL‐R (Figures S3–S4), which may reflect differential responses to pathogens with varying lifestyles. Further, under natural infection in the field, NIL‐R consistently showed higher ALS resistance than NIL‐S and 9930 (Figure S5).

Gy14 shows moderate resistance to PM caused by the biotrophic *Px* fungal pathogen (He et al. 2013). In the greenhouses, both Gy14 and NIL‐R exhibited an anti‐chlorosis effect on natural *Px* infection (Figure S6a). QTL analysis in the Gy14 × 9930 RIL population revealed a major‐effect anti‐chlorosis QTL colocalized with *CsSGR* (Figure S6b). The 3:1 segregation of the anti‐chlorosis effect among 80 NIL‐R×NIL‐S F_2_ plants (61 ‘yellowing’ vs. 19 ‘staygreen’; *p* = 0.796 in χ^2^ test against 3:1) suggests a single recessive gene underlying the ‘staygreen’ phenotype in this population. We measured CHL content and genotyped the *A323G* causal SNP among these F_2_ plants. Single marker analysis of the data revealed a significant association of the resistance allele with high CHL contents (Figure S6c), indicating that *Cssgr* contributed to the anti‐chlorosis effect against PM infection. There was no difference in anti‐sporulation scores to *Px* infection between the NILs.

While the expression of all four *CCGs* (*CsSGR, CsPPH, CsPAO* and *CsRCCR*) was upregulated upon *Pcu, Cor* or *Psl* infection in both NILs, only *CsSGR* and *CsPPH* showed significantly and consistently higher expression in NIL‐S than NIL‐R at each time point (Figures 1f–i; S3f–i and S4f–i), indicating increased pathogen‐induced Chl degradation in NIL‐S. Higher expression of *CsPAO* in NIL‐R was observed at 7 dpi only in response to *Pcu* and *Psl*, and there was no difference in the expression of *CsRCCR* at any time point for all pathogens, supporting the critical role of the *A323G* mutation in *Cssgr* in alleviating pathogenesis‐induced chlorosis and disease severity by reducing Chl degradation and maintaining photosynthesis efficiency.

**Figure 2:**
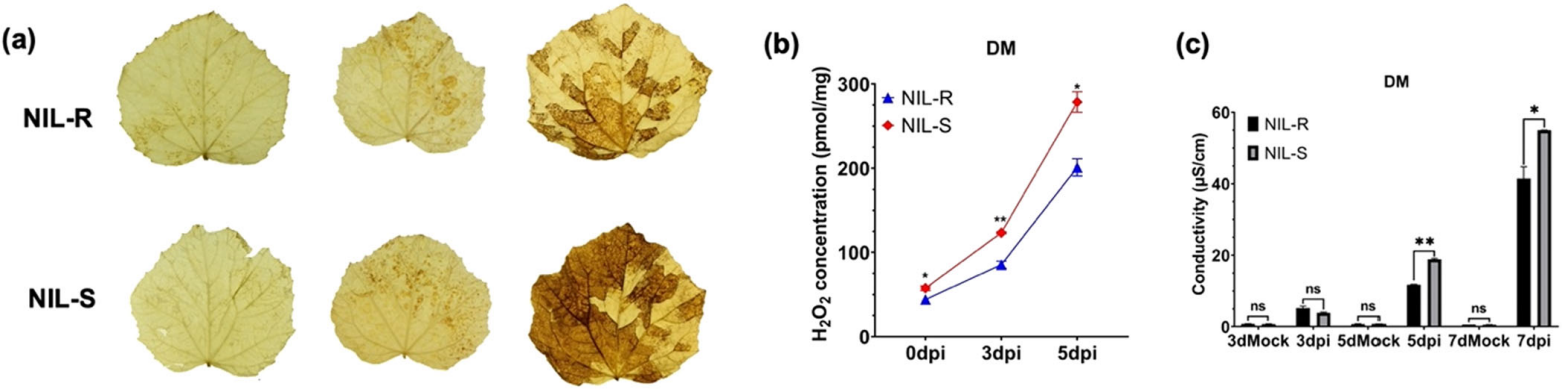
*Cssgr* (*A323G*) mutation reduces ROS accumulation and cell death upon *Pcu* infection. (a, b) Less H_2_O_2_ production in leaves of NIL‐R than in NIL‐S before and after *Pcu* inoculation. In (a), images from left to right are taken at 0, 3, and 5 dpi, respectively. Production of H_2_O_2_ was detected with DAB staining. (c) Cell death assay by measuring electric conductivity (EC) in NILs before and after *Pcu* inoculation with water as the control (mock). Error bar = mean ± SD (*n* = 3). ns = not significant; * and ** indicate *p* < *0.05* and *0.01*, respectively, based on pair‐wise *t*‐tests. [Color figure can be viewed at wileyonlinelibrary.com]

ROS are key players in both plant immunity and pathogenesis. We quantified H_2_O_2_ accumulation in leaves of the two NILs at 0, 3 and 5 dpi (Figures 2a,b and S7a,b). NIL‐R constitutively (at 0 dpi) accumulated less H_2_O_2,_ which continued to increase in both NILs, with more in NIL‐S for all three pathogens. Furthermore, there was more H_2_O_2_ production in response to *Pcu* inoculation than *Cor* and *Psl*. ROS accumulation often results in oxidative stress and cell death, which was significantly mitigated in NIL‐R (Figures 2c and S7e–f). Collectively, these data indicated that *Cssgr*‐mediated MDR can reduce Chl degradation, pathogenesis‐induced chlorosis, ROS accumulation, and cell death, which are associated with reduced expression of *CCGs*.

### 3.2 Knock‐Out *CsSGR* Mutant Alleles Enhance MDR and Chilling Tolerance

**Figure 3:**
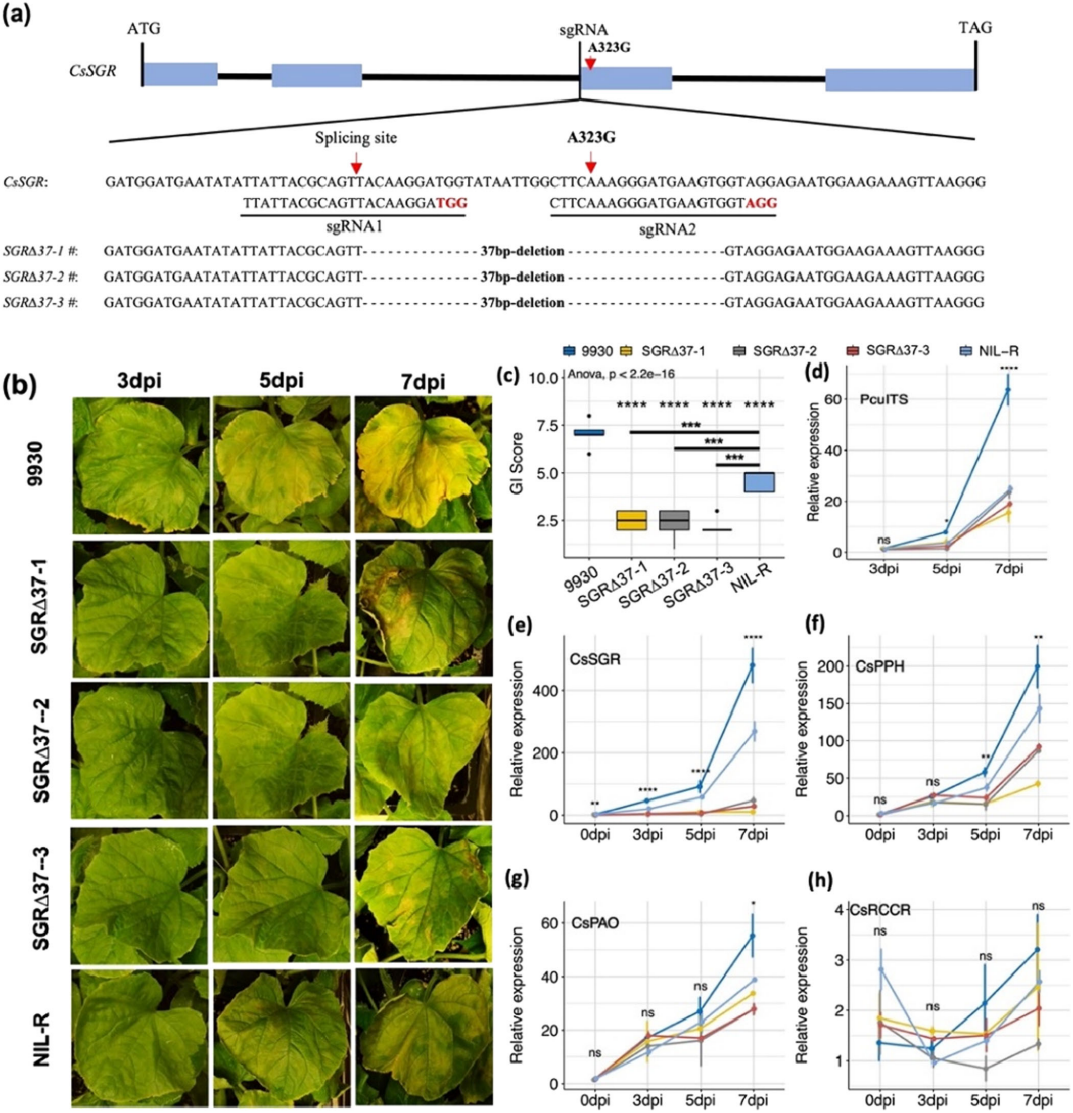
Development and characterisation of *CsSGR* KO mutants with gene editing. (a) Location of gRNA targets of *CsSGR* and three mutants from independent editing events. Three KO mutants, *SGRΔ37‐1/−2/−3* carry the same 37‐bp deletion including the intron‐exon splicing junction site (red arrowhead) and part of the third exon. (b) DM symptom development on three mutants, NIL‐R and 9930 at 3, 5, and 7 dpi under growth chamber conditions. (c) Box plots of mean disease scores showing three KO mutants have higher DM resistance than NIL‐R. Experiment‐wide significant test among genotype means is performed with ANOVA. Nonparametric tests are performed to examine differences in mean DM scores of three KO mutants against that of NIL‐R. In all mutants, upon inoculation, *Pcu* growth reflected from the relative expression level of *PcuITS* from qPCR was significantly repressed (d) compared with the WT (9930). After inoculation, the expression *CsSGR*, *CsPPH* and *CsPAO* (e‐g) is significantly lower in all mutant lines than in 9930 in at least one timepoint but no difference in the expression of *CsRCCR* (h) is observed among all lines. In d‐h, bars = mean ± SD (*n* = 3). ns = not significant; *, **, ***, and **** indicate *p* < 0.05, 0.01, 0001, and 0.0001, respectively based on pair‐wise *t*‐tests. [Color figure can be viewed at wileyonlinelibrary.com]

We performed CRISPR/Cas9‐based editing of the ‘9930’ susceptible allele (WT) (Figure 3a). Three independent, homozygous knock‐out (KO) mutant lines carrying an identical 37‐bp deletion (*SGRΔ37‐1, −2 and −3*) were obtained (Figure S8).

No additional sequence variants were found within *CsSGR*, including the promoter region (Figure S9). Further examination of the SGRΔ37‐2 transcriptomes identified no structural variation at predicted off‐target sites. This 37‐bp deletion would introduce a stop codon, resulting in a premature SGR protein (Figure S8). Under greenhouse conditions, all three KO lines exhibited ‘staygreen’ appearance on adult plants and mature fruits when the WT plants or fruit already turned yellow (Figure S10), which was not observed between NILs, suggesting *SGRΔ37* is a stronger allele than the *A323G* SNP for the ‘staygreen’ phenotype.

Upon inoculation by *Pcu* (Figure 3b–d), *Cor* (Figure S11), and *Psl* (Figure S12), the three KO lines showed significantly lower disease scores than NIL‐R (Figure 3c). A similar trend was also observed in a field trial under natural *Pcu* infection (Figure S13): the anti‐chlorosis effect of the three KO lines was comparable to that of PI 190788 that has a very high DM resistance controlled by multiple QTL (Wang et al. 2018) further supporting that *SGRΔ37* is a stronger allele than *A323G* for MDR. By 7 dpi, the growth of *Pcu* and *Psl*, but not *Cor*, was effectively and non‐differentially inhibited on all resistant lines (Figures 3d; S11c–12c). Consistent with the resistance levels, both *CsSGR* and *CsPPH* had significantly lower expression in all KO lines than in the WT; their expression in NIL‐R was intermediate between KO lines and 9930 (Figures 3e, S12d and S13d).

We questioned if NIL‐R and *SGRΔ37* mutants also possess resistance to other pathogens. NIL‐R and three KO lines showed non‐differentially higher resistance than the WT to the necrotrophic *Cca* with typical HR (Figure S14). All KO lines also showed a clear anti‐chlorosis effect on the PM pathogen (Figure S6d). These observations suggest that while the mutations confer higher disease resistance to all pathogens, the resistance appears more effective against infection by hemibiotrophic or necrotrophic pathogens than against that by biotrophic ones. We also tested the NILs and KO mutants for responses to low‐temperature (LT) stress with chilling‐sensitive 9930 and Gy14 as controls. While the control plants showed leaf yellowing and accelerated senescence, all three KO lines displayed staygreen with lower damage scores and less chlorosis; NIL‐R showed intermediate responses (Figure S15).

Further, the enhanced MDR in the KO mutants does not seem to negatively impact their horticultural performance. In greenhouse experiments, data for plant architecture, fruit quality, or yield‐related traits were collected from the NILs, KO lines, and 9930, including MFT, FFT, LBN, TNN, VL, and parthenocarpic fruit yield (FW per plant). No difference among these lines was found for these traits except the FT which was delayed by 2–3 days in the KO lines compared to 9930 or NIL‐R (Figure S16) which is consistent with the ‘staygreen’ nature of the mutation.

### 3.3 *Cssgr*‐Mediated MDR Is Associated With Transcriptomic Reprogramming for Allele‐Dependent Passive and Positive Defence Against Infection by Pathogens With Different Lifestyles

Transcriptome profiling was performed on NIL‐R, NIL‐S, SGRΔ37‐2, and 9930 with 23 samples collected at 0, 3 and 5 dpi with *Pcu* and *Cor* (Table S3). PCA revealed good reproducibility and significant effects of the treatments among the transcriptomes (Figure S17). DEGs were identified from 11 pairwise comparisons (NIL‐R vs. NIL‐S, and SGRΔ37‐2 vs. 9930) (Tables S4–S5), and 8 time‐wise comparisons in each line (3 vs. 0 dpi and 5 vs. 3 dpi). Enriched GO terms in Biological Processes (BPs) were identified with GO analysis. A subset of ~ 600 DEGs was manually annotated and categorised based on their possible functions in pathogen defence (Table S5).

#### 3.3.1 SGRΔ37‐2 Shows Constitutively Stronger Expression of Photosynthesis‐Related Genes, Lower Expression of CCGs and SAGs

**Figure 4:**
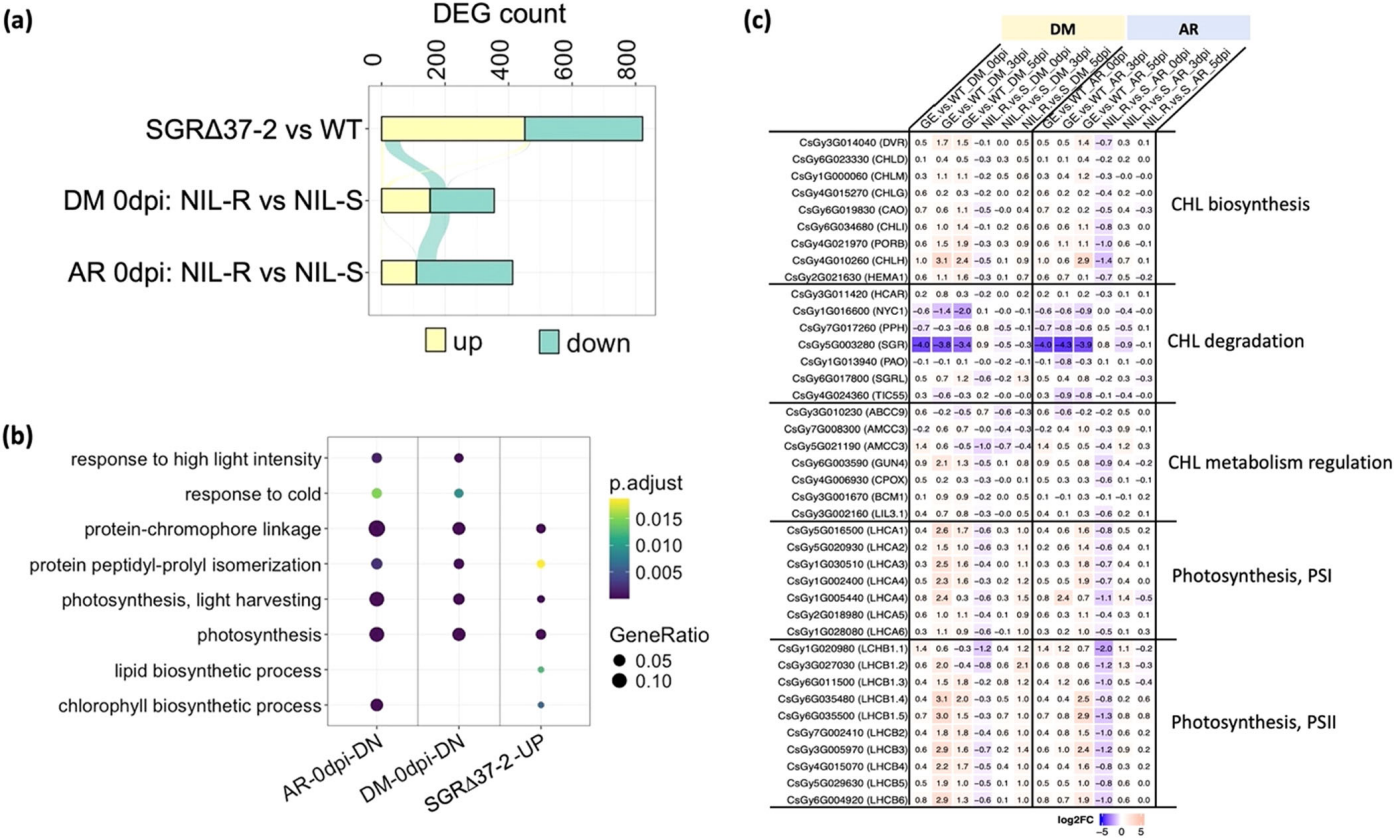
Analysis of constitutively and differentially expressed genes (DEGs) in NILs and SGRΔ37‐2 knock‐out plants in response to *Pcu* (DM) or *Cor* (AR) inoculations. (a) Alluvial plot showing numbers of constitutive DEGs from three comparisons (at 0 dpi). WT = 9930 cucumber. (b) Enriched GO biological process terms of up‐ (UP) and downregulated (DN) constitutive DEGs in different transcriptomes. DEG sets with no significantly enriched GO terms are not shown. (c) Heatmap of log_2_(FoldChange) values of selected constitutive DEGs in NIL‐R versus NIL‐S, and GE (gene edited; SGRΔ37‐2) versus WT (9930) comparisons that are involved in chlorophyll biosynthetic and degradation pathways, as well as LHC family proteins (LHCAs of PSI and LHCBs of PS II). [Color figure can be viewed at wileyonlinelibrary.com]

We first compared transcriptomes at 0 dpi, aiming to identify constitutive DEGs among the four lines. There were 822, 413 and 355 constitutive DEGs in SGRΔ37‐2 versus WT, NIL‐R‐AR versus NIL‐S‐AR, and NIL‐R‐DM versus NIL‐S‐DM comparisons, respectively (Figure 4a; Table S3). Upregulated DEGs in SGRΔ37‐2 were enriched in photosynthesis, CHL metabolism, and related BPs, which, however, were enriched in downregulated DEGs in NIL‐R (Figure 4b). A heatmap of the expression level of selected DEGs in Chl metabolism and LHCI/II is illustrated in Figure 4c (details in Table S6). All LHC protein‐coding genes (*LHCA1‐6* of PSI, *LHCB1‐6* of PSII; total 17) showed constitutively higher expression in SGRΔ37‐2 and NIL‐R than in 9930 or NIL‐S, respectively. Three *CCG*s (*CsSGR*, *CsPPH*, and *CsNYC1*) had lower expression in SGRΔ37‐2 than in 9930, with *CsSGR* having the most reduced expression in SGRΔ37−2, but they had either non‐differential expression between NILs (at 0 dpi_AR) or upregulated (e.g., *PPH*) in NIL‐R (at 0 dpi_DM) (Table S6; Figure S18a,b). Meanwhile, four CHL biosynthetic genes (*CHLG*, *CHLI*, *PROB*, or *CAO*) showed constitutively higher expression in SGRΔ37‐2, but lower or non‐differential expression in NIL‐R versus NIL‐S. Many constitutive DEGs in the KO mutant regulate CHL metabolism (e.g., *CPOX*, *GUN4*, and *AMCC3*, Guo et al. 2013; Ishikawa et al. 2001; Larkin et al. 2003; Richter et al. 2023) and/or JA and SA‐mediated defence responses (e.g., *UGT76B1/CsGy4G018140* for UDP‐glycosyltransferase, von Saint Paul et al. 2011; Bauer et al. 2021) (Table S5). Collectively, these observations are consistent with phenotypic data that *SGRΔ37* is a stronger allele than *A323G* in maintaining photosynthesis capacity and the delay of senescence. The constitutive expression level of pathogen defence‐related DEGs seems consistent with the degree of MDR in the KO mutant lines and NIL‐R.

#### 3.3.2 CsSGR Mutants Show Stronger Expression of Photosynthesis‐Related Genes and Reduced Expression of CCGs and SAGs in Response to Pathogen Infection

**Figure 5:**
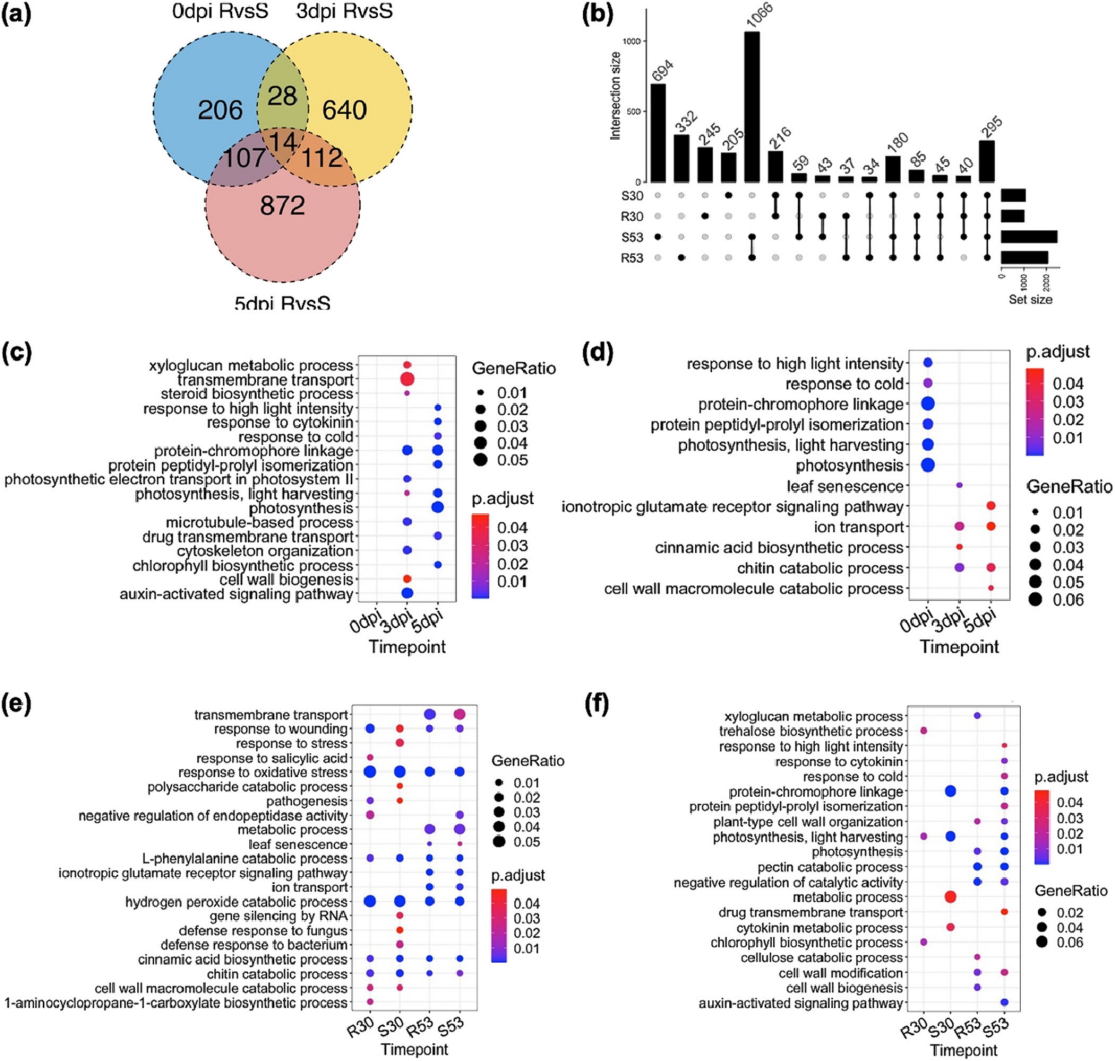
Transcriptome profiling of NIL‐R and NIL‐S in response to *P*. *cubensis* infection. (a) Venn diagram of numbers of DEGs at 0, 3, and 5 dpi in comparisons of NIL‐R versus NIL‐S. (b) UpSet plot showing shared DEGs from comparisons of transcriptomes in and between two NILs at different time points post *Pcu* inoculation. (c) and (d) are top enriched Biological Process GO terms for up‐ and downregulated DEGs in NIL‐R versus NIL‐S comparisons at 0, 3 and 5 dpi, respectively. (e) and (f) are top enriched BP GO terms of up‐, and downregulated DEGs from comparisons between 3 versus 0 and 5 versus 3 dpi in two NILs, respectively. [Color figure can be viewed at wileyonlinelibrary.com]

We identified DEGs in the transcriptomes of SGRΔ37‐2 versus 9930, and NIL‐R versus NIL‐S at 3 and 5 dpi with *Pcu* and *Cor* (Tables S3 and S4; Figures 5a and S19–S21a), as well as DEGs from 3 versus 0 and 5 versus 3 dpi comparisons in each transcriptome (UpSet plots in Figures 5b and S19–S21b). Top enriched BP GO terms for these DEGs were also identified (Figures 5c–f and S19–S21c–f). A subset of representative DEGs associated with Photosynthesis and senescence, Cell death and oxidative stress responses, as well as Phytohormone‐associated stress responses, are exemplified in supplemental Tables S6–S8, respectively.

Overall, in response to *Pcu* and *Cor* infection, expression of CHL biosynthetic (e.g., *HEMA1, CAO*, *BCM1*, and *DVR*) and LHCI/II (e.g., *LHCAs* and *LHCBs*) genes was suppressed while expression of CHL degradation (e.g., *SGR*, *PPH*, and *NYC1)* was upregulated in all lines; however, the magnitude of changes of these DEGs was significantly lower in resistant (KO and NIL‐R) than in susceptible (NIL‐S and 9930) lines (Figure 4C; Tables S5 and S6). This was largely consistent with their constitutive expression patterns supporting more active CHL biosynthesis, and less CHL degradation in SGRΔ37‐2 than in NIL‐R, which agrees with the higher MDR of the KO mutants. Interestingly, the expression of *CsSGRL* was downregulated in all lines with disease progression; by 5 dpi, its expression was higher in resistant ones, indicating its possibly different roles from *CsSGR* in response to pathogen infection in cucumber.

In agreement with the enriched ‘leaf senescence’ GO term, most SAGs were upregulated upon pathogen infection in all genotypes, which, however, were downregulated in KO and NIL‐R plants either constitutively or induced by *Pcu/Cor* infection (Table S6). Some examples include NAC TFs (*NAC029/046/083*), autophagy‐related genes (*ATG18c/g*), and SAGs (*AAF, APX4*, and *SAG1/20/21*) (Kim et al. 2016; Guo et al. 2021; Masri and Kiss 2023). In Arabidopsis, NAC029 is a positive regulator of senescence by promoting ABA production and CHL degradation (Yang et al. 2014). Cucumber *CsNAC029* (*CsGy5G023470*) showed constitutively lower expression in SGRΔ37‐2 but a higher expression in NIL‐R versus NIL‐S; its expression remained downregulated at 3 and 5 dpi upon *Pcu* infection in SGRΔ37‐2 but was at the same expression level between NILs (Table S6). Timewise, *CsNAC029* increased its expression in response to *Pcu* inoculation in all lines, which was the opposite upon *Cor* infection further supporting their mutant allele‐ and pathogen lifestyle‐dependent expression. Consistent with the delay of senescence, many BPs for normal growth (e.g., ‘DNA replication initiation,’ ‘auxin‐activated signalling pathway,’ and ‘response to cytokinin’) were enriched in upregulated DEGs in SGRΔ37‐2 or NIL‐R (e.g., genes in the auxin/IAA and cytokinin pathways) suggesting robust growth and development of the resistant plants under pathogen infection.

**Figure 6:**
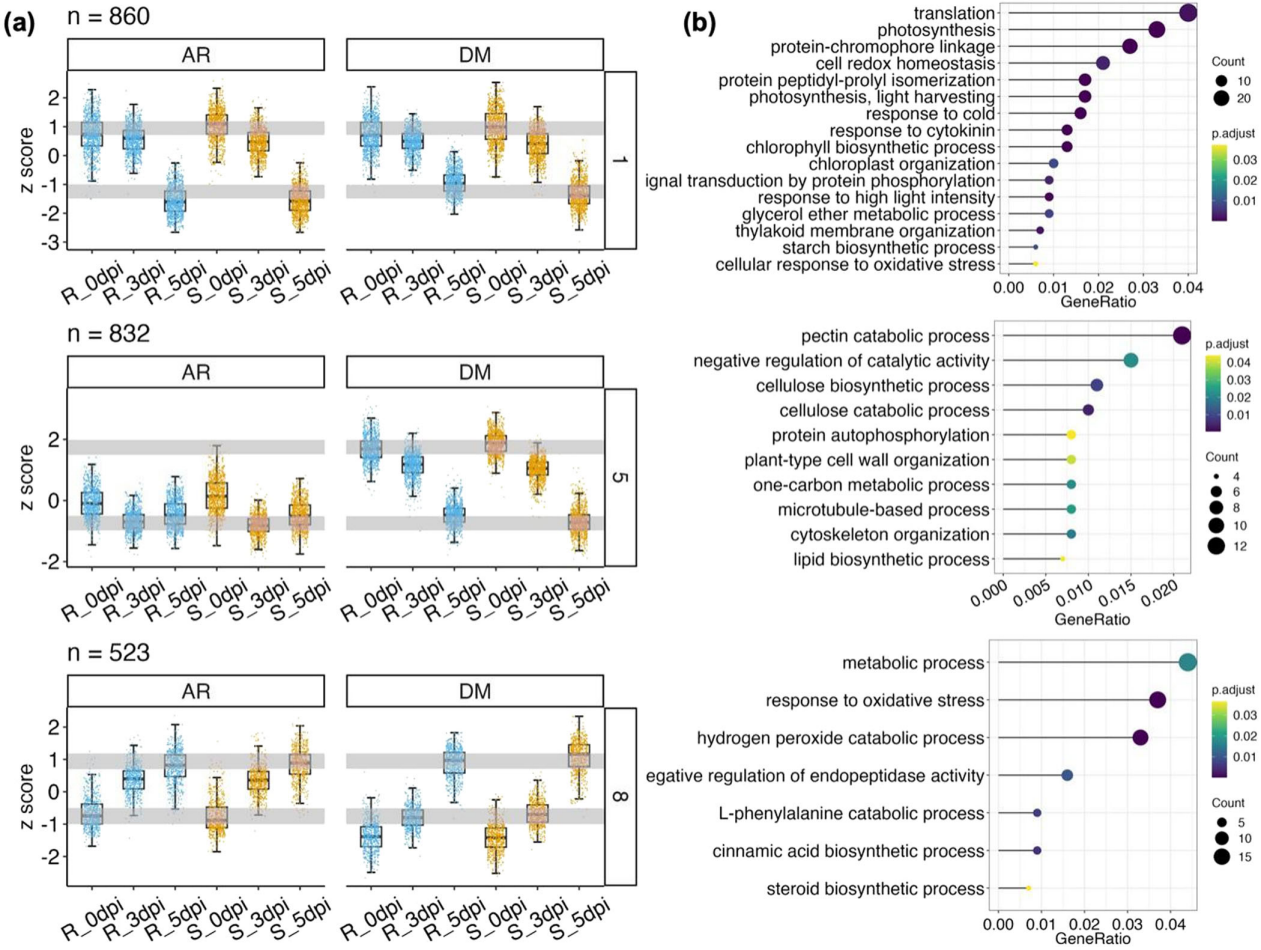
Representative co‐expression modules and enriched GO terms in transcriptomes of NIL‐R and NIL‐S in response to *Pcu* (DM) and *Cor* (AR*)* infection. (a) Barplot visualisation of three gene co‐expression modules. (b) Top enriched GO terms of biological processes in each co‐expression module. [Color figure can be viewed at wileyonlinelibrary.com]

Gene co‐expression analysis on NIL transcriptomes identified 13 co‐expression modules (1–13) (Figures 6 and S22); each module represents largely consistent and unique expression patterns in NIL‐R and NIL‐S. For example, Modules 1 and 5 featured downregulated genes, while Modules 7, 8, 10, and 11 comprised upregulated genes induced by pathogen infection. Most photosynthesis‐related DEGs belonged to Modules 1 and 5 that were enriched in relevant GO terms such as ‘Photosynthesis’, and ‘Light‐harvesting complex’ (Figure 6b). Thus, the expression of these genes was suppressed with pathogen infection, but to a lesser degree in NIL‐R.

#### 3.3.3 DEGs in KO or NIL‐R Lines Imply Reduced Cell Death and Stronger Antioxidant or Detoxification Capabilities

Pathogen infection or perturbation of the photosynthesis systems generates ROS, resulting in oxidative stress and cell death in host plants. Plants have developed a complex antioxidant system of defence for ROS scavenging and detoxification including enzymatic antioxidants (e.g, APX, CAT, DHAR, GR, PER, and SOD) or nonenzymatic compounds (e.g., AA, GSH, α‐tocopherol, carotenoids, and flavonoids) (García‐Caparrós et al. 2021). NIL‐R accumulates less H_2_O_2_, thus less cell death than NIL‐S (Figures 2 and S7), suggesting activation of the antioxidant systems of defence to mitigate oxidative stresses in NIL‐R. In agreement with this, DEGs in NIL and KO lines were enriched in relevant BP GO terms like ‘Photosynthetic electron transfer in PSII’, ‘Response to oxidative stress’, and ‘H_2_O_2_ catabolic process’ (Figures 5 and S19–S21). Many DEGs are associated with oxidative stress‐related cell death and the synthesis of antioxidants or detoxification compounds (Table S7). Their expression patterns support stronger antioxidative stress abilities in the resistant lines. For example, four autophagy (ATG)‐related genes (*ATGc/f/g/1010*, Hanaoka et al. 2002; Lenz et al. 2011) showed constitutively lower expression in SGRΔ37‐2 or were downregulated in SGRΔ37‐2 and NIL‐R in response to *Pcu* infection. Other positive regulators for cell death with similar expression patterns included *HIR1*, *DCD*, *KT11*, *CAD1*, and *BFN1* (Farage‐Barhom et al. 2008; Qi et al. 2011). Conversely, negative regulator genes for cell death were upregulated in SGRΔ37‐2 or NIL‐R (e.g., *PLA2A*, *Thf1*, and *BCS1* (Zhang et al. 2014). Genes encoding almost all categories of enzymatic or nonenzymatic antioxidants showed differential expression in the KO and NIL transcriptomes (Tables S5 and S7); many were in Co‐Expression Module 8 (Figure 6a), suggesting their positive roles in antioxidative stress responses. Interestingly, most TRX family (10 of 11), MATE family (11 of 12), and carotenoid biosynthetic pathway (5 out of 6) genes were upregulated either constitutively or in response to pathogen infection in both SGRΔ37‐2 and NIL‐R. On the other hand, most members in the *GST* and *PER* gene families showed lower expression in resistant lines but increased expression in both lines with the progression of the diseases (Table S7).

#### 3.3.4 CsSGR‐Conferred MDR Is Associated With SA‐ and JA‐Mediated Defence Responses With Distinct Transcriptomic Landscapes in Biotrophic and Necrotrophic Phases of Infection

Among the ~ 600 manually annotated DEGs (Table S5), many were involved in the SA/SAR, and JA biosynthesis/signalling pathways that play critical roles in plant immunity. Relevant GO BF terms like ‘response to SA’ and ‘phenylalanine catabolic process’ were enriched in upregulated DEGs in NIL‐R (Figures 5 and S20). DEGs for many key players in the SA/SAR/JA pathways in response to *Pcu/Cor* inoculations (Table S8) included those for SA biosynthesis (*PALs, CM1*, *CM2*, *FMO1*s, *ICS*) and signalling (e.g., *EDS4, NPR3, PAD4, CBP60B/C/G, SARD1*), JA metabolism (e.g., *LOX1‐3, ACX, AOC, AOS, KAT, JMT, JOX, PLA*, *CYP94B1*) and signalling (e.g., *NAC019, MYC2, MYC4, JAZ1‐4*), and genes regulating JA/SA crosstalk (e.g., *WRKY33/40/51/53/54/70, MYB44, CBP60C*). A positive correlation was not observed for all DEGs between their expression patterns and disease resistance/susceptibility of these lines. This is reasonable because a gene may be a positive or negative regulator in pathogen defence; its expression may also depend on the pathogen's lifestyle (biotrophic and hemibiotrophic). Nevertheless, the transcriptome data support the role of the SA/JA pathways in active defence against DM and AR. Many genes that positively or negatively regulate SA/JA‐mediated pathogen defence were, respectively, up‐ or downregulated in resistant lines (constitutively or induced), including genes for the synthesis of antimicrobial proteins or antioxidants like PR proteins (PR‐2, PR5, PR‐14, and dirigent proteins) (Table S7). For example, the Arabidopsis NAC019 is a negative regulator of plant immunity, which activates *MYC2* to inhibit SA accumulation and upregulates the expression of *AtSGR1* or *AtPAO* to promote CHL degradation (Zheng et al. 2012; Zhu et al. 2015). In SGRΔ37‐2 and NIL‐R, *CsMYC2* and *CsNAC019* were downregulated both constitutively and in response to *Pcu* and *Cor* inoculation, implying a critical role of the SA pathway in *Cssgr*‐conferred MDR.

The data also reveals differential dynamic changes in response to infection by biotrophic *Pcu* and hemibiotrophic *Cor*. The transition from biotrophic to necrotrophic phase of *Cor* growth occurs from 3 to 5 dpi (Gan et al. 2013). The temporal expression patterns of many DEGs in SA/SAR/JA defence pathways seem to support their antagonistic relationships in the biotrophic and necrotrophic phases of *Cor* infection: they were upregulated from 0 to 3 dpi in response to infection of both *Pcu* and *Cor*; which, from 3 to 5 dpi, kept increasing in their expression in NILs in the case of *Pcu* (continuing biotrophic phase) but were downregulated during switching from biotrophic to necrotrophic growth of *Cor* (Tables S7 and S8). *WRKY53* is a positive and negative regulator of SA and JA signalling, respectively (Hu et al. 2012). *CsWRKY53* was upregulated in both NILs from 0 to 3 dpi upon *Pcu* and *Cor* infection; from 3 to 5 dpi, its expression continued to increase for *Pcu* but not for *Cor*. Furthermore, *CsWRKY53* expression was slightly higher in SGRΔ37‐2 than in WT at 3 and 5 dpi of *Pcu*, which was similar in response to *Cor* infection. These data suggest that SA‐ and JA‐mediated defence work at the biotrophic and necrotrophic phases, respectively, for *Cor*, and the expression level varies between strong and weak alleles.

Phenotypically, *Cssgr*‐mediated AR resistance exhibits typical HR while anti‐chlorosis plays a more important role in DM resistance (Figures 1, S3 and S4; Pan et al. 2018). Many genes involved in SA‐mediated pathogen defence, including SAR and HR were upregulated in SGRΔ37‐2 mutant or NIL‐R at 3 dpi in response to *Cor* inoculation but downregulated in NIL‐R in response to *Pcu* (Table S8). For example, *FMO1* (*CsGy1G028220*) is an essential component of SAR and a marker gene for the cell death pathway (Mishina and Zeier 2006; Koch et al. 2006). These data suggest that elevated expression of genes in the SA pathway may contribute to HR upon *Cor* infection in the KO lines and NIL‐R.

Taken together, from the extensive transcriptomic analysis, we may reach the following conclusions. (1) *CsSGR* loss‐of‐susceptibility mutations can down‐regulate expression of *CCGs*, slow down Chl degradation, and delay pathogen‐induced senescence, which allows the plants to maintain active CHL biosynthesis and robust photosynthesis, thus mitigating damages caused by pathogen infection (passive defence). (2) Mutations in *CsSGR* also activate active defence against pathogen infection. The defence involves mainly the SA‐ and JA‐mediated signalling pathways, which seem to play different roles in the biotrophic and necrotrophic phases during infection by the hemibiotrophic *Cor* pathogen. (3) The *SGRΔ37* is a stronger allele than the *A323G* SNP in conferring MDR.

### 3.4 *CsSGR* Mutations Impair Physical Interactions With Itself and Key Players in the Photosynthetic Machinery

Transcriptomic profiling reveals a complex gene regulatory network associated with *Cssgr*‐conferred MDR. How do these weak and strong mutant *CsSGR* alleles impact its structure, function, and interactions with other players in the regulatory network? The CsSGR protein contains a chloroplast transit peptide domain, a highly conserved SGR domain, and a variable C‐terminal region (Figure S23). We confirmed the chloroplast location of CsSGR with subcellular localisation assays (Figure S24a–e). Two days after infiltration, leaf spots infiltrated with the WT (CsSGR^WT^) construct turned yellow faster than those with the CsSGR^Q108R^ mutant (Figure S24f). Consistent with this, total CHL content in tobacco leaf spots infiltrated with CsSGR^WT^ was significantly lower than that infiltrated with CsSGR^Q108R^ (Figure S24g). In another independent experiment, we examined transient overexpression of the WT, *Q108R*, and *Δ37* alleles in tobacco leaves (Figure S25a), and found that the CsSGR^Δ37^ exhibited a stronger loss‐of‐function phenotype than CsSGR^Q108R^ in CHL degradation: leaf spots infiltrated with the CsSGR^Δ37^ construct remained fully stay‐green, while CsSGR^Q108R^ showed partial yellowing (Figure S25a) supporting *CsSGR* ^*Δ37*^ being a stronger allele than *CsSGR* ^*Q108R*^ in reduction of CHL degradation in tobacco.

To assess the consequences of different mutations in *CsSGR*, we first modelled the protein structures of CsSGR^WT^, CsSGR^Q108R,^ and CsSGR^Δ37^ mutants with AlphaFold (Jumper et al. 2021), which predicted that the Q108R amino acid substitution would impact conformational change, SGR dimer formation in the C‐terminal region, while the 37 bp deletion would completely abolish the dimer formation and its interaction with other partners (Figure S26). To experimentally validate this, we first investigated the self‐interactions of CsSGR^WT^, CsSGR^Q108R^, and CsSGR^Δ37^ with Y2H and quantitative β‐galactosidase activity assays (Figure S27) which indicated that the dimer formation of SGR was impacted in CsSGR^Q108R^ and was almost undetected in CsSGR^Δ37^ in vitro (Figure S27a,b). However, initial Co‐IP assays using the flag‐IP unexpectedly detected self‐interaction for all constructs (CsSGR^WT^, CsSGR^Q108R^, and CsSGR^Δ37^), including weak bands in negative controls (Figure S27c). To address potential false positives, we employed a GFP‐trap IP system. Two independent replicate assays (Figure S28d–e) using this system confirmed the self‐interaction for CsSGR^WT^ and CsSGR^Q108R^ (albeit weaker in the WT) but detected no interaction for CsSGR^Δ37^. The immunoblot analysis further confirmed that CsSGR^Δ37^ failed to form dimers at 48 h after infiltration (Figure S25b), which validated the Co‐IP results on CsSGR self‐interaction. Thus, these data suggest that mutations in *CsSG*R significantly impair its dimerisation which seems important to its functions and interactions with its partners in CHL degradation and photosynthesis pathways.

**Figure 7:**
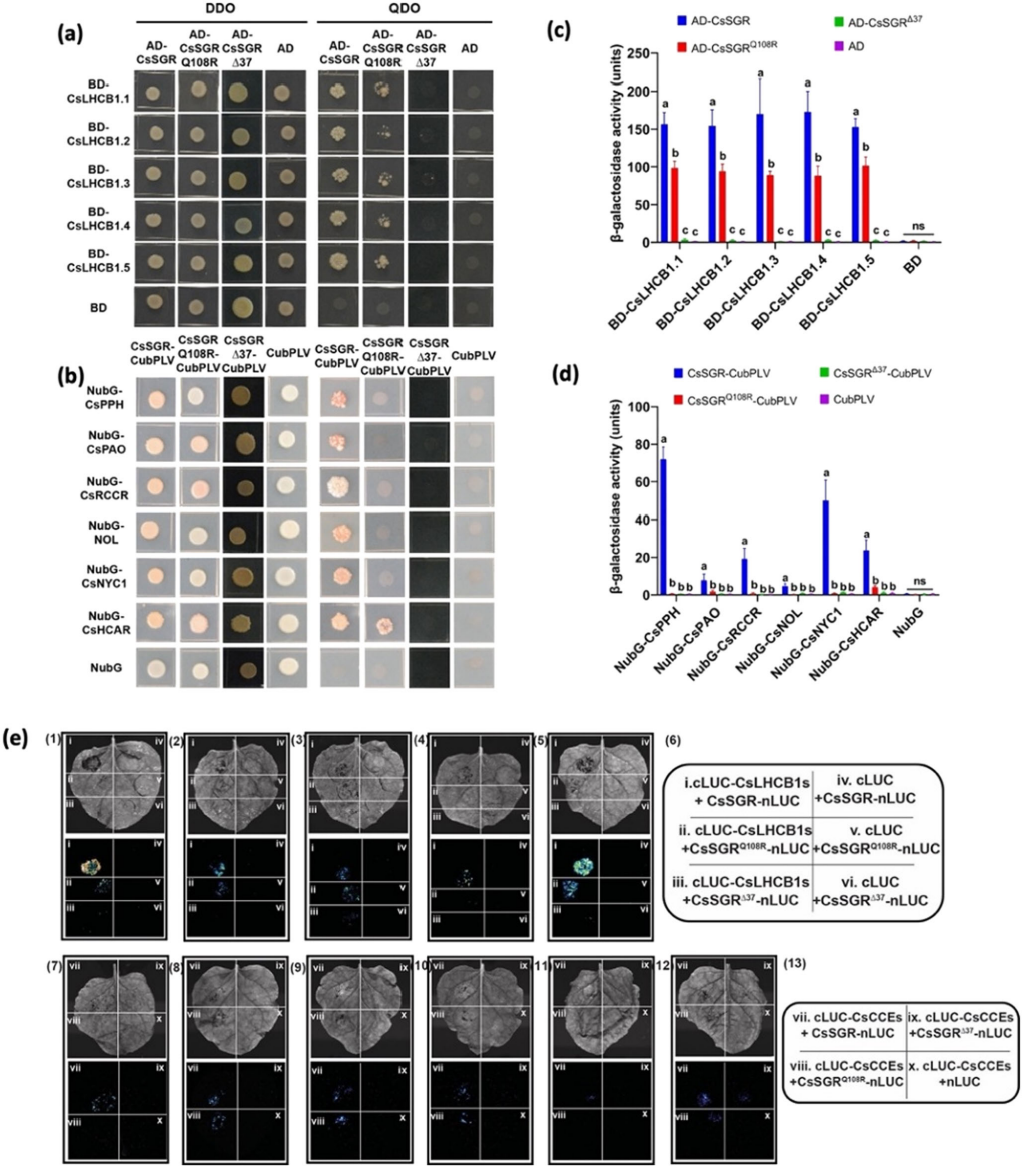
Mutations in *CsSGR* impair its interactions with LHCB1s of PSII and CsCCEs in the chlorophyll degradation pathway. (a) and (b) Y2H assays reveal interactions of CsSGR (WT) with all five LHCB1 and six CCE proteins, respectively, which are reduced in CsSGR^Q108R^, and almost abolished in *SGRΔ37* KO mutant line. In each case, the CsSGR, CsSGR^Q108R^ or CsSGRΔ37 CDS is fused to the activation domain (AD), and each of CsLHCB1s or CCEs is fused to the binding domain (BD), grown on quadruple dropout (SD‐Ade‐His‐Leu‐Trp) medium (QDO). Transformed AH109 (for a) or THY. AP4 (for b) yeast strains were also grown on the double dropout (SD‐Leu‐Trp) medium (DDO) to confirm growth capacity. (c) and (d) Quantitation of interactions of CsSGR/CsSGR^Q108R^/CsSGR^Δ37^ with CsLHCB1s and CsCCEs in yeast β‐galactosidase activity assays, respectively. Each value = mean ± SD (*n* = 3). Means with different letters are significantly different based on Tukey's tests. (e) Luciferase Complementation Imaging (LCI) assays of interactions between CsSGR/CsSGR^Q108R^/CsSGR^Δ37^ and CsLHCB1s or CsCCEs. (1) to (5) are CsLHCB1.1 to CsLHCB1.5, respectively. (6) Layout of different combinations between cLUC and nLUC plasmids for interaction analysis of CsSGR/CsSGR^Q108R^/CsSGR^Δ37^ with CsLHCB1s. (7) to (12): Interactions of CsSGR with CsPPH, CsPAO, CsRCCR, CsNOL, CsNYC1, and CsHCAR, respectively. (13) Layout pf distribution of different combinations between cLUC and nLUC plasmids for interaction analysis of CsSGR/CsSGR^Q108R^/CsSGR^Δ37^ with CsCCEs. [Color figure can be viewed at wileyonlinelibrary.com]

Previous studies have shown that SGR interacts with other CCEs in the CHL degradation pathway and LHCBs of PSII. We hypothesised that critical mutations inside *CsSGR* may impair or disrupt those interactions and thus compromise chloroplast functions, and the degree of disruption varies among different mutant alleles. With Y2H assays, we examined interactions of different CsSGR constructs (CsSGR^WT^, CsSGR^Q108R^, and CsSGR^Δ37^) with five members of the cucumber LHCB1 family (LHCB1.1‐1.5) and six CCEs (PPH, PAO, RCCR, NOL, NYC1, and HCAR). We found that CsSGR^WT^ interacts with all 11 proteins, which was significantly reduced in CsSGR^Q108R^, and completely abolished in CsSGR^Δ37^ (Figure 7a,b). Consistent with this, quantitative β‐galactosidase activity assays revealed significantly lower enzymatic activities for interactions of CsSGR^Q108R^ with all LHCB1s and CCEs, which were undetectable for CsSGR^Δ37^ (Figure 7c,d). All these interactions were confirmed in LCI (Figure 7e) and BiFC (Figure S28). We performed further co‐IP pull‐down assays of the three CsSGR alleles with two LHCB1s (LHCB1.1, LHCB1.3) and two CCEs (PPH, PAO), which further validated the findings from Y2H, and BiFC analyses (Figure S29).

**Figure 8:**
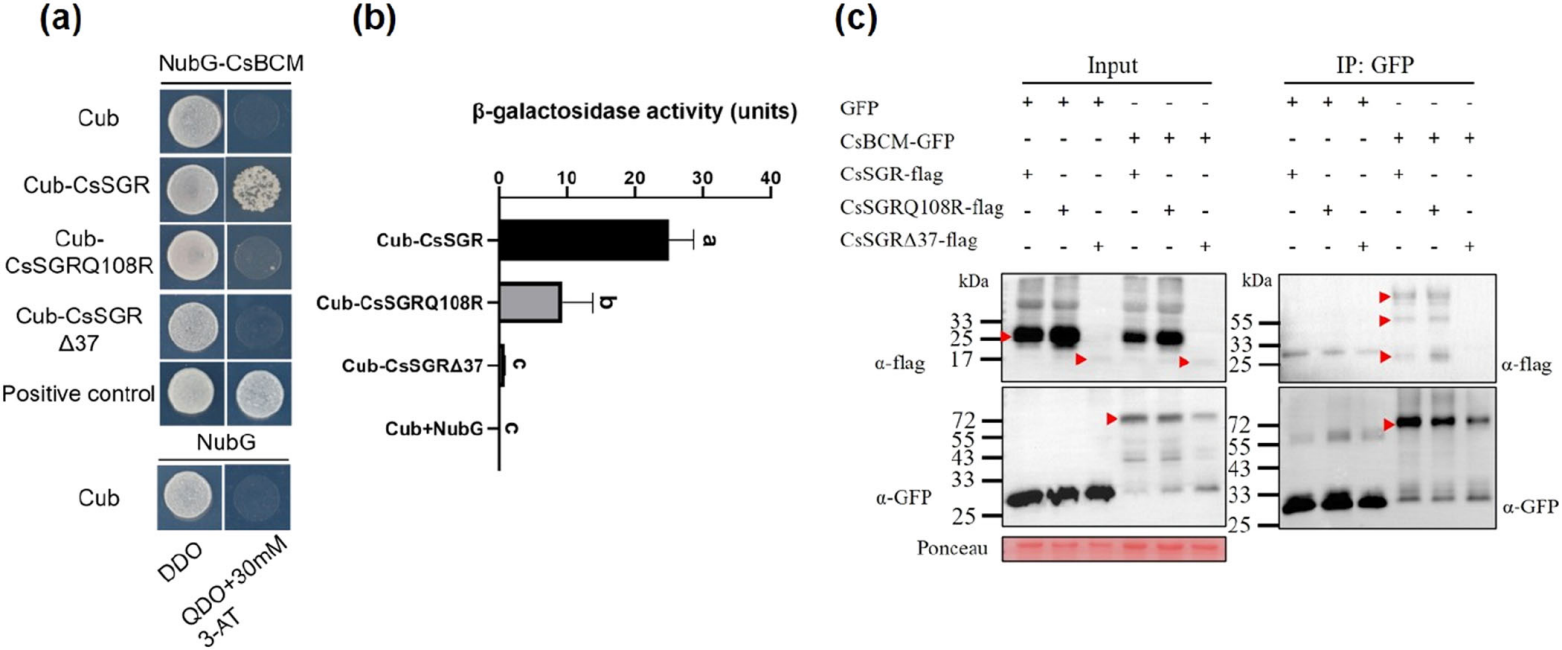
Mutations in *CsSGR* impair its interactions with CsBCM in the chlorophyll biosynthesis pathway. (a) Y2H assays of PPIs of CsSGR^WT^, CsSGR^Q108R^, and CsSGR^Δ37^ with CsBCM. (b) Quantitation of interactions between CsBCM and three CsSGR protein isoforms in yeast β‐galactosidase activity assays. Each value = mean ± SD (*n* = 3). Means with different letters are significantly different based on Tukey's tests. (c) Validation of interactions between CsSGR^WT^, CsSGR^Q108R^, and CsSGR^Δ37^ and CsBCM by Co‐IP assays. [Color figure can be viewed at wileyonlinelibrary.com]

In Arabidopsis, *AtBCM1/2* regulate CHL homoeostasis by stimulating CHL synthesis via interaction with GUN4 and/or inhibiting CHL catabolism by targeting AtSGR1 for degradation (Wang et al. 2020; Zhang et al. 2020; Yamatani et al. 2022). The C‐terminal region of AtSGR1 seems critical for its interaction with AtBCM1 (Fu et al. 2024). Upon inoculation with *Pcu* or *Cor*, many genes in the CHL biosynthesis or regulatory pathway, including CsGUN4 and CsBCM (a homologue of AtBCM1), were significantly upregulated in CsSGR^Q108R^ or CsSGR^Δ37^ mutants (Tables S5 and S6). We reasoned that this upregulation of CHL biosynthesis genes in the mutants may be associated with disrupted CsSGR‐BCM1 interaction. Indeed, Y2H assay confirmed the physical interactions between CsSGR and CsBCM, which was significantly reduced by CsSGR^Q108R^, and completely abolished by CsSGR^Δ37^ (Figure 8a). Consistent with this, quantitative β‐galactosidase activity assays revealed significantly reduced activities for CsSGR^Q108R^‐CsBCM, which was undetectable for CsSGR^Δ37^‐CsBCM (Figure 8b). Finally, Co‐IP assays confirmed interaction between CsBCM and WT CsSGR or CsSGR^Q108R^, but not with CsSGR^Δ37^ (Figure 8c).

Taken together, these data suggest that mutations in *CsSG*R significantly impair its self‐interaction, and interactions with its partners in CHL degradation and biosynthesis/regulation pathways, as well as key photosystem II/II components with *SGRΔ37* as a much stronger allele than the SNP in disrupting such interactions.

## 4 Discussion

### 4.1 Strong Alleles Through Gene Editing Enhance *CsSGR*‐Conferred MDR

Here we show that both NIL‐R and SGRΔ37 KO lines have MDR against DM, AR, ALS, PM, and TLS with the *SGRΔ37* being a stronger allele (Figures 2, S3–S6, S13 and S14). Among the many *SGR* mutants studied in the literature, only those in cucumber (Pan et al. 2018; Y. Wang et al. 2019), soybean (Chang et al. 2019), and rice (Xie et al. 2024) were shown to be associated with disease resistance in the field (i.e., *R* genes in plant breeding). There may be multiple reasons for the limited reports of *SGR* mutants with field disease resistance. For example, some plants like Arabidopsis have two functionally redundant copies of *SGR*; the *SGRL* gene may also interact with SGRs (Sakuraba et al. 2014; Kuai et al. 2018). A more plausible explanation is the varying effects of different alleles of the *SGR* locus. Most reported SNP mutations are located in the central SGR domain (Figure S23) which seem less impactful (weak allele) than mutations carrying SNPs or large indels (strong allele) resulting in a truncated SGR protein missing the C‐terminal region that is critical for SGR functions (Xie et al. 2019; Figure S23). For example, two tomato KO mutants with 1 and 123 bp deletions in *SlSGR* show lower infected areas than WT upon infection of the necrotrophic pathogen *Botrytis cinerea* with the 1‐bp deletion mutant being less resistant than the 123‐bp deletion line (Gianoglio et al. 2022). In this study, the *CsSGRΔ37* (strong allele) confers higher MDR than the *A323G* SNP (weak allele). Visually, all three *SGRΔ37* KO mutants show delayed senescence and ‘staygreen’ phenotype on adult plants and fruits (Figure S10), which was difficult to discern between NILs (Figure S2). Overall, our work reveals an important role of the *SGR* in conferring MDR that could be enhanced with strong alleles through gene editing.

Previous studies have shown that some *SGR* mutations are associated with abiotic stress tolerance (e.g., Dong et al. 2023). We also observed higher cold tolerance of three SGRΔ37 mutant lines and NIL‐R than that of NIL‐S (Figure S15). Several BP GO terms were highly enriched in multiple transcriptomes such as ‘Responses to ABA’, ‘Response to oxidative stress’, ‘Response to cold’ and ‘Response to stress’ (Figures 5 and S20). Although no abiotic stresses were applied on the test lines, there were many DEGs involved in the ABA (34 DEGs) and ET (50 DEGs) pathways, and crosstalk of phytohormone signalling, which are regulators of both biotic and abiotic stress responses (Table S8). For example, three negative transcription regulators of ABA‐mediated abiotic stress responses, *CsGy4G016820* and *CsGy6G019690* for NAC002; *CsGy6G004000* for DIVARICATA) were downregulated either constitutively or in response to pathogen infection.

### 4.2 Both Passive and Active Defences Are Involved in *CsSGR‐*Conferred MDR

In this study, resistant KO mutant and NIL‐R plants had reduced expression of *CCGs* and *SAGs*, and higher expression of photosynthesis‐related genes, which seem to be consistent with work in the tomato *SlSGR1* KO line carrying a 19‐bp deletion (Kim et al. 2024) and *Brassica napus* plants with complete loss of *BnSGR* (Qian et al. 2016). Qian et al. (2016) suggested that the more robust growth in *BnSGR*‐deleted lines is due, at least partially, to the counteraction by strong linkage to neighbouring gene(s) with positive effects on plant growth and development. We did not observe any linkage drag in *SGRΔ37* or *A323G* mutant lines on their horticulture performances (Y. Wang et al. 2019; Figures S2 and 16). Among the 10 predicted genes in the 100 kb region harbouring *CsSGR*, none except *CsSGR* showed differential expression between SGRΔ37‐2 and 9930 (Figure S18b) suggesting that the non‐negative growth is due to the *CsSGR* mutations rather than effects from nearby ones.

We found that the *SGRΔ37* mutation had a consistently stronger expression than the *A323G* SNP (weak allele) for genes involved in photosynthesis or Chl catabolism. How could this be linked with SGR structure and functions? As a CCE, SGR participates in the early obligatory events of destabilization of the LHC proteins in which SGR interacts with all other CCEs and LHCII proteins (Jiang et al. 2007; Park et al. 2007; Hortensteiner 2006; Kusaba et al. 2013; Sakuraba et al. 2012, 2014). LHC degradation promotes further senescence and Chl degradation (Tanaka and Ito 2024). In the KO and NIL plants, 17 *LHCA* and *LHCB* genes were differentially expressed (Table S6; Figure S4c). Interestingly, most of these genes exhibited higher constitutive or pathogenesis‐induced expression in SGRΔ37‐2 mutant than in 9930 which were downregulated in NIL‐R versus NIL‐S (Table S7). Multiple protein‐protein interaction assays (Y2H, BiFC, co‐IP, Immunoblotting, or β‐galactosidase activity) found that CsSGR^WT^ interacts with itself, all six other CCEs, all five LHCB1s, and CsBCM; however, such interactions were significantly reduced in CsSGR^Q108R^ mutant or completely abolished (undetectable) in CsSGR^Δ37^ KO mutant (Figures 7, 8 and S27–S29), which is consistent with the predicted 3D structures of the two mutant alleles (Figure S26). Interestingly, while Co‐IP assays of selected interactions confirmed Y2H results, we observed weaker interactions of CsSGR^Δ37^ with PPH or PAO (Figure S27b). This weak interaction signal may be due to nonspecific binding of CsSGR^Δ37^ to another protein, which binds PPH/PAO in planta. Despite this weak interaction being detected through Co‐IP, *SGRΔ37* acts as a complete loss‐of‐function allele, evidenced by abolished CHL degradation and a complete stay‐green phenotype. We also noticed that in Co‐IP, signals of WT CsSGR were consistently weaker than CsSGR^Q108R^, contrasting with Y2H results. We speculate that the *CsSGR* ^*Q108R*^ mutation enhances protein stability in planta, leading to its higher accumulation and stronger Co‐IP signals. In Arabidopsis, AtSGR1/2 form homo‐ and/or heterodimers during leaf senescence (Sakuraba et al. 2012); in wheat, self‐interaction of TaSGR1 has been confirmed both in vitro and in vivo (Li et al. 2017). Furthermore, the redox regulation affects the conformation of the AtSGR (monomer or dimer) (Xie et al. 2019), and thioredoxin TaTrx catalysers the transformation of TaSGR1 oligomers into monomers (Li et al. 2025). This redox‐sensitivity likely contributes to the conformation of SGR, which may explain the weak signals observed in Co‐IP assays of SGR self‐interaction. Overall, compared with the weak allele (*Q108R*), the strong allele (*SGRΔ37*) results in more disruption of its interactions with other CCEs and LHCB1s and less Chl degradation, and more delay of the onset of senescence, and ultimately less damage, lighter disease symptoms (staygreen), and more robust growth and development of infected plants. That is, the loss‐of‐function *CsSGR* mutations provide a passive defence against pathogen infections.

However, extensive transcriptome profiling of these lines reveals a complex, *SGR*‐associated gene regulatory network for active pathogen defence in mutant plants that involves the activation of the SA/JA pathways. Other than as a site for photosynthesis, and primary and secondary metabolism (e.g., synthesis of defence compounds and phytohormones), the chloroplast also acts as an environmental sensor critical for plant immunity (Domínguez and Cejudo 2021; Littlejohn et al. 2021; Lee and Kim 2024). Any internal or external perturbation can influence the photosynthetic electron transport chain (ETC) of PSI and PSII resulting in ROS accumulation (e.g., ^1^O_2_, and H_2_O_2_). The ROS can cause oxidative stress and damage, but at the same time can also trigger context‐dependent RS pathways to reprogramme nuclear gene expression to maintain cellular homoeostasis by mitigating oxidative damage and regulating processes such as photosynthesis, cell death, and detoxification (Richter et al. 2023; Lee and Kim 2024). In response to pathogen infection, the RS pathways are intertwined with SA and JA defence pathways (Yang et al. 2021; Bali et al. 2023). Here we found that NIL‐R had significantly less ROS (H_2_O_2_) accumulation and cell death than WT before and after pathogen infection (Figures 2 and S7), which is consistent with observations in Arabidopsis *AtSGR1* (Mur et al. 2010) and rice *OsSGR* (Jiang et al. 2011) mutants. Many genes involved in SA and JA signalling, PCD, detoxification or synthesis of antioxidants, PR proteins, or defence chemicals showed differential expression between the resistant and susceptible lines (Tables S4–S7) implying the involvement of SA‐ or JA‐mediated defence against infection of these pathogens. Furthermore, many DEGs (*GUN4, GLK1/2, SAL1*, *FtsHs*, and *PUB22/23*) are key players in RS pathways for SA‐dependent defence (Lee et al. 2023; Nedo et al. 2024; Larkin et al. 2003; Richter et al. 2023; Yamatani et al. 2022; Lee et al. 2023; Tachibana et al. 2024). However, additional work may be needed to confirm the role of RS‐activated SA/JA defence in *Cssgr*‐conferred MDR.

### 4.3 *Cssgr*‐Conferred MDR and Abiotic Stress Tolerance in Cucumber: A General Mechanism Explaining Enhanced Biotic/Abiotic Stress Tolerance in Mutants of Genes in Plant Photosystem Machinery?

**Figure 9:**
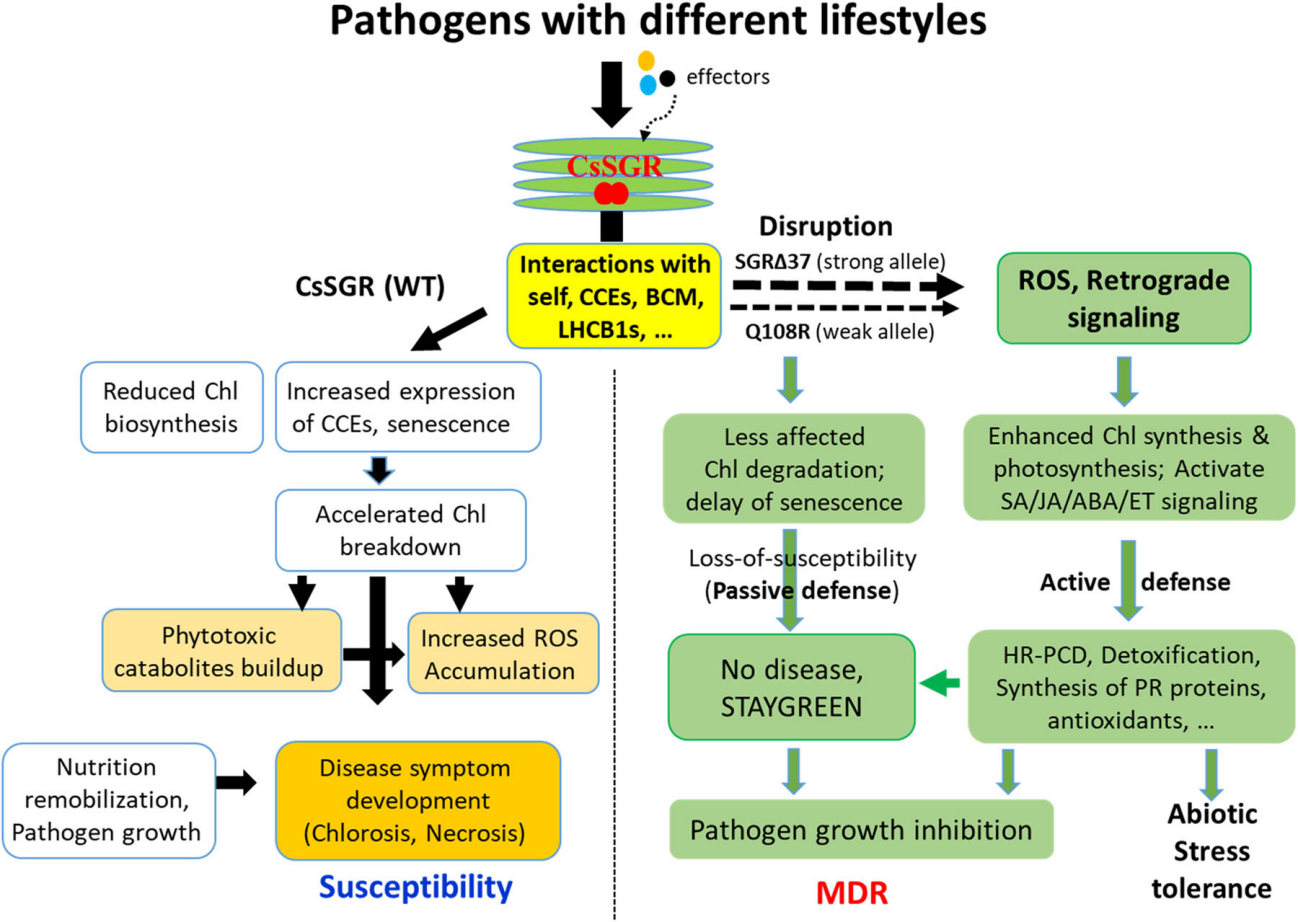
An improved working model explaining possible mechanisms of *Cssgr*‐mediated multiple disease resistance (MDR) in cucumber. The responses upon pathogen infection in the susceptible and resistant plants are depicted to the left and right of the vertical dashed line, respectively. *CsSGR* is necessary for disease symptom development. Mutations in this gene will disrupt interactions of CsSGR with its partners such as LHCB1s and other CCEs which will inhibit normal functions of the chlorophyll degradation pathway genes and delay senescence thus providing passive defence. At the same time, the disruption generates ROS which act as retrograde signalling molecules to trigger SA/JA defence pathways against pathogen invasion, which provides active defence. Note that strong and light dashed lines represent the degree of interaction disruptions caused by strong and weak mutant alleles, respectively, which is correlated with disease resistance levels. It is also possible that effectors from different pathogens may interact with CsSGR to modulate differential responses. The two overlapped, red‐filled circles represent possible dimers (self‐interaction) that may be important for its function. The drawing is revised from Y. Wang et al. (2019). [Color figure can be viewed at wileyonlinelibrary.com]

We previously proposed a model emphasising the passive, loss‐of‐susceptibility strategy for *A323G*‐mediated broad‐spectrum disease resistance in cucumber (Y. Wang et al. 2019). The present study provides further evidence of SA‐/JA‐mediated active defence in *Cssgr*‐conferred MDR. This study also reveals a more complex gene regulatory network associated with *CsSGR* mutations for enhanced chilling tolerance. Thus, these findings allow the development of an improved working model as described in Figure 9. This model depicts the loss‐of‐susceptibility nature of *CsSGR* mutations, which provides passive defence against pathogen infections. Meanwhile, in the mutants, the disruption of self‐interaction of CsSGR and its interactions with LHCB1s, CCEs, and BCM results in ROS‐mediated retrograde signalling for active defence for MDR and enhanced chilling tolerance in *CsSGR* ^*Q108*^ and *CsSGR* ^*Δ37*^ mutants. The level of MDR or chilling tolerance is dependent on the nature of the mutations with *CsSGR* ^*Δ37*^ being a stronger allele than *CsSGR* ^*Q108*^.

From a broader perspective, our model proposed herein seems to support a general mechanism in plants in which ROS generated from perturbation of photosynthesis/plastid biogenesis are associated with RS‐triggered defence responses against pathogen infection or increased susceptibility. For example, Arabidopsis RPH1 is an intrinsic thylakoid protein that promotes cytochrome b559 formation during PSII. The *rph1* mutant is susceptible to the oomycete pathogen *Phytophthora brassicae*, and silencing of the potato homologue *StRPH1* in a resistant potato cultivar causes susceptibility to the late blight pathogen *Phytophthora infestans* (Belhaj et al. 2009; Che et al. 2024). In rice, an SNP in the *LHCB5* of LHCII confers broad‐spectrum blast fungus (*Magnaporthe oryzae*) resistance through ROS accumulation in the chloroplast (Liu et al. 2019). *CPOX* encoding a coproporphyrinogen III oxidase in the tetrapyrrole biosynthesis pathway is involved in disease resistance and SA‐dependent cell death (Ishikawa et al. 2001; Guo et al. 2013). In the transcriptomes, cucumber homologues of multiple *CsLHCB* genes, *CsCPOX*, or *CsRPH1* were mostly upregulated either constitutively or induced by *Pcu/Cor* inoculation in the KO mutant or NIL‐R plants (Tables S6 and S7). However, none of these genes carry non‐synonymous mutations in NIL‐R. The association of increased expression of these genes with MDR is unknown.

Finally, while the *Cssgr* mutations confer MDR, there are differences in defence responses, and thus the level of resistance among the resistant lines to infection by pathogens with different lifestyles. Phenotypically, the *A323G* SNP and *SGRΔ37* alleles have similar and more effective resistance against the necrotrophic pathogen *Cca* (Figure S14). The resistance exhibits anti‐chlorosis for biotrophic pathogens (*Pcu* and *Px*), while for necrotrophic *Cca* and hemibiotrophic *Cor*, both anti‐chlorosis and anti‐necrosis are obvious with typical HR responses (Pan et al. 2018; Figures S3, S4 and S14). At the transcriptome level, many genes involved in SAR and HR in the SA‐mediated pathogen defence pathway show intensified expression at 3 dpi in response to *Cor* infection (Table S8) indicating a significant shift of the transcriptome landscape in the host in response to the shift of the *Cor* growth from biotrophic to necrotrophic phases (Gan et al. 2013; Harata et al. 2016). This contrasts with the early notion that plants share similar SA‐mediated defence responses against biotrophic and hemibiotrophic pathogens (Glazebrook 2005). Such differences in defence response may also explain the stronger resistance of the *sgr* mutations against infection by necrotrophic pathogen *Cca*. Likely, upon infection of *Cca*, *SGR* expression was more suppressed, with more delay of chlorophyll degradation, and pathogenesis‐resulted in leaf senescence. The lower accumulation of ROS may induce hypersensitive responses, less oxidative damage by *Cca* infection, and at the same time stronger defence responses. These outcomes may impact more on the *Cca* pathogenesis that thrives on dead tissue resulting in less severe disease symptoms. It is also possible that different elicitors from pathogens of different lifestyles may target *CsSGR* during plant‐pathogen interactions. For example, Li, Khan, et al. (2024) found that Q108R mutant protein in Gy14 cucumber but not the WT interacts with a secreted effector protein (CSEP30) from the PM pathogen (*Px*) to elicit a defence response against PM. The roles of *CsSGR*, retrograde signalling, and pathogens of different lifestyles played during this process merit further investigation.

## Conflicts of Interest

The authors declare no conflicts of interest.

## Supporting information

SGR supp figures PCE R1.

SGR_PCE_Supp tables.R1.

## Data Availability

All data pertinent to the reported work have been provided in the manuscript or in the supplemental online materials. Transcriptome data from Illumina sequencing have been deposited at the NCBI with Sequence Read Archive (SRA) #SUB14903339.
