# Supplementary material for: Photosystem Perturbation by Staygreen Mutations Confers Allele‐Dependent Defences Against Infections of Pathogens With Different Lifestyles and Abiotic Stress Tolerance
Source: Plant Cell Environ. 2025 Oct 13;49(1):585–603. doi: 10.1111/pce.70229 (PMC12675977; doi:10.1111/pce.70229)
Supplement: Supplementary file 1 — SGR supp figures PCE R1. [file PCE-49-585-s002.docx]

## Supporting Information

Title: **Photosystem Perturbation by *STAYGREEN* Mutations Confers Allele-dependent Defenses against Infections of Pathogens with Different Lifestyles and Abiotic Stress Tolerance**

Authors: Junyi Tan, Zhejuan Tian, Feifan Chen, Kang Gao, Jinghao Jin, Anthony P. Keinath, Ronald D. Dymerski, Zhiming Wu, Yiqun Weng

The following Supporting Information is available for this article:

**Fig. S1** A schematic diagram of chlorophyll biosynthetic and degradation pathway in plants.

**Fig. S2** Development and characterization of near isogenic lines (NILs) for the *dm1/cla/psl* locus.

**Fig. S3** Characterization of anthracnose (AR) resistance in near-isogenic resistant (NIL-R) and susceptible (NIL-S) lines for the *dm1/cla/psl* locus.

**Fig. S4** Characterization of angular leaf spot (ALS) resistance in near-isogenic resistant (NIL-R) and susceptible (NIL-S) lines for the *dm1/cla/psl* locus.

**Fig. S5** Performance of ALS resistance of NIL-S and NIL-R in response to natural *Psl* infection under field conditions.

**Fig. S6** Performance of powdery mildew (PM) resistance of NILs and QTL analysis of PM resistance in Gy14 cucumber.

**Fig. S7** H_2_O_2_ and cell death induced by infection of pathogens is mitigated in NIL-R.

**Fig. S8** Alignment of *CsSGR* coding region sequences from *CsSGR*-KO lines and WT.

**Fig. S9** Alignment of *CsSGR* promoter sequences from *CsSGR*-KO lines and WT.

**Fig. S10** The stay-green effect observed from *CsSGR* knock-out plants on plant senescence (a) and mature fruit skin color (b).

**Fig. S11** Evaluation of AR response for *CsSGR* knock-out plants.

**Fig. S12** Evaluation of ALS response for *CsSGR* knock-out plants.

**Fig. S13** Evaluation of downy mildew responses of *CsSGR* knock-out lines in open field.

**Fig. S14** Evaluation of TLS response for *CsSGR* knock-out plants.

**Fig. S15** Evaluation of cold stress responses among *CsSGR* knock-out plants.

**Fig. S16** Evaluation of horticultural traits for *CsSGR* knock-out, WT and NIL-R and NIL-S plants in greenhouse trials.

**Fig. S17** PCA analysis of RNA-seq data in this study.

**Fig. S18** Analysis of constitutively and differentially expressed genes in SGRΔ37-2 KO plants without pathogen infection.

**Fig. S19** Analysis of differentially expressed genes (DEGs) in NIL-R and NIL-S in response to artificial inoculation of the AR pathogen (*Cor*).

**Fig. S20** Analysis of differentially expressed genes (DEGs) in SGRΔ37-2 and 9930 in response to artificial inoculation of the DM pathogen, *P. cubensis*.

**Fig. S21** Analysis of differentially expressed genes (DEGs) in SGRΔ37-2 and 9930 in response to artificial inoculation of the AR pathogen (*Cor*).

**Fig. S22** Co-expression modules identified from gene co-expression analysis of transcriptomes of NIL-R and NIL-S in response to *Pcu* and *Cor* infection**.**

**Fig. S23** Domain structure of SGR homologs.

**Fig. S24** Transient overexpression of CsSGR and CsSGR^Q108R^ through agrobacterium injection in *N. benthamiana*.

**Fig. S25** Western blot analysis of CsSGR/ CsSGR^Q108R^/CsSGRΔ37 protein.

**Fig. S26.** Predicted protein structures encoded by three *CsSGR* alleles with AlphaFold program.

**Fig. S27** Mutations in *CsSGR* impair its self-interaction.

**Fig. S28** BiFC assays of interactions between CsSGR/ CsSGR^Q108R^/CsSGRΔ37 and CsLHCB1s or CsCCEs in the epidermal cells of *N. benthamiana*.

**Fig. S29** Co-IP analysis of the interaction of CsSGR/ CsSGR^Q108R^/CsSGR^D37^ with LHCB1.1 (a), LHCB1.3 (a), PPH (b), or PAO (b).


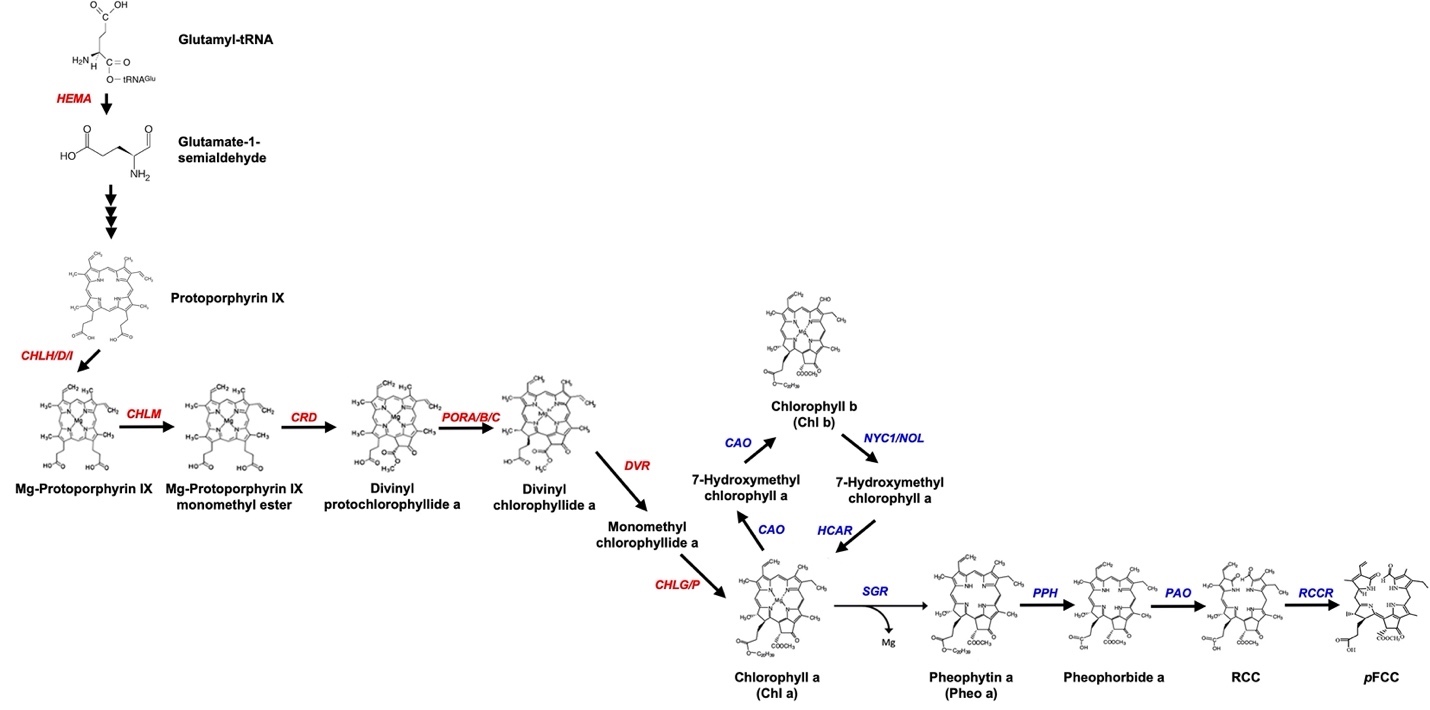


**Fig. S1 A schematic diagram of chlorophyll biosynthetic and degradation pathway in plants. HEMA,** glutamyl-tRNA reductase; **CHLH/D/I**, Mg-chelatase; **CHLM**, Mg-protoporphyrin IX methyltransferase; **CRD**, Mg-protoporphyrin IX monomethylester cyclase; **PORA/B/C**, protochlorophyllide oxidoreductase; **DVR**, divinyl chlorophyllide a 8-vinyl-reductase; **CHLG/P**, chlorophyll synthase; STAY-GREEN (**SGR**), Magnesium-dechelatase; **PPH**, Pheophytinase; **PAO**, Pheophorbide a oxygenase; **RCC**R, red chlorophyll catabolite reductase; **CAO,** Chlorophyll a oxygenase; NON-YELLOW COLORING1 (**NYC1**) and NYC1-LIKE (**NOL**), chlorophyll b reductases; **HCAR**, 7-Hydroxymethyl chlorophyll a reductase.

**
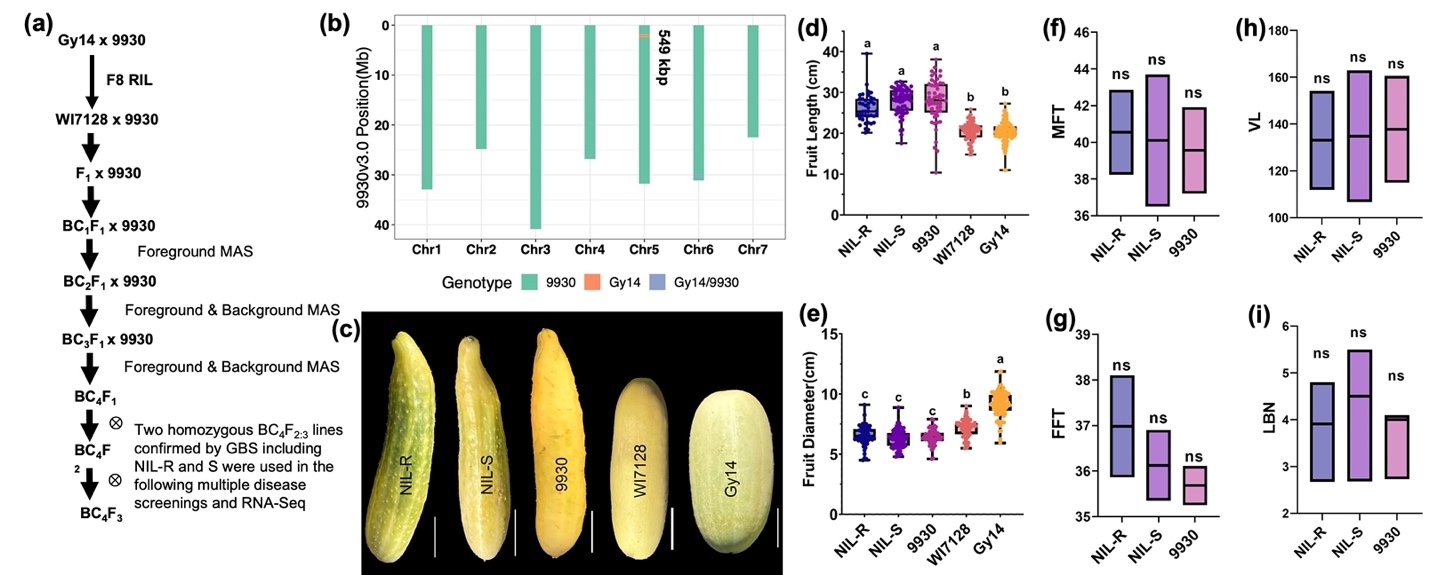
**

**Fig. S2 Development and characterization of near isogenic lines (NILs) for the *dm1/cla/psl* locus.** (**a**) Scheme of marker-assisted development of NILs. (**b**) Genotypic characterization of the NIL-Resistant (NIL-R) and NIL-Susceptible (NIL-S) lines by genotyping-by-sequencing (GBS). Orange: Gy14 alleles (donor); Green: 9930 alleles (recipient). The introgression (orange color) of the Gy14 allele in the ‘9930’ background in NIL-R is 549 kb in size (1,845,275-2,394,460 bp, 9930v3.0). (**c-e**): Appearances (**c**), length (**d**), and diameter (**e**) of mature fruits of NIL-R, NIL-S, 9930, WI7128 (F_8_ RIL and resistance allele donor), and Gy14 collected from the field. In **c** and **d**, error bars represent the standard deviation (36 fruits per line). Means that do not share a letter are significantly different based on Tukey’s tests. (**f-g**) Floating bar plots showing the mean values of MFT (male flowering time, day) and FFT (female flowering time, day) collected from the 2021 and 2022 open field trials. (**h**) A floating bar plot displays the mean values of VL (vine length, cm) collected from the 2022 and 2023 open field trials. (**i**) A floating bar plot shows the mean values of LBN (lateral branch number) collected from the 2021, 2022, and 2023 field trials. For the multi-year open field trials, an ANOVA test was performed to test if there is a significant difference among genotypes. ns=not significant.

**
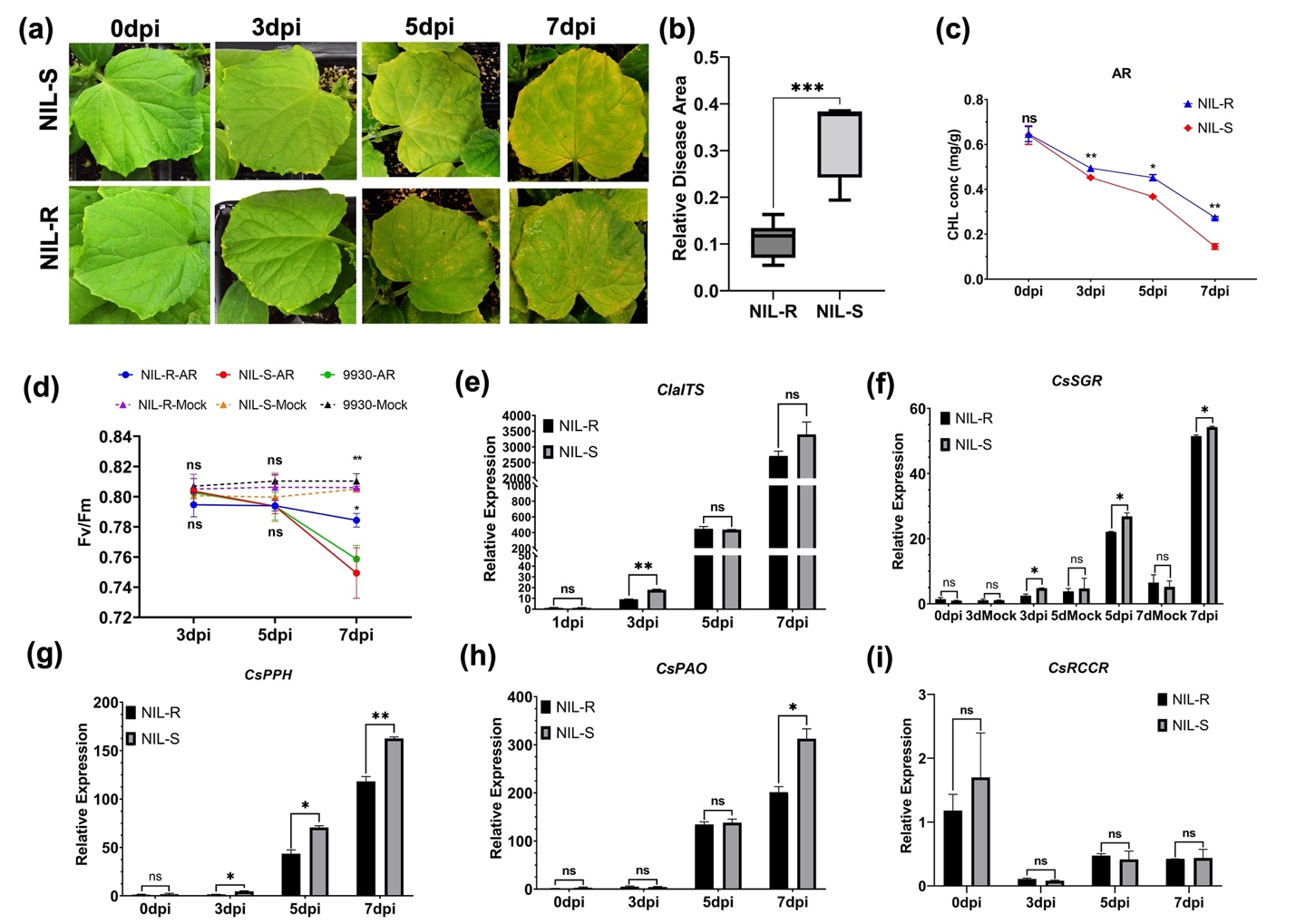
**

**Fig. S3. Characterization of anthracnose (AR) resistance in near-isogenic resistant (NIL-R) and susceptible (NIL-S) lines for the *dm1/cla/psl* locus.** (**a**) AR symptom development on the leaves of two NILs at different days post artificial inoculation (dpi) of *C. orbicular*e (*Cor*) under growth chamber conditions. (**b**) Bar graphs of mean AR disease areas of two NILs. Upon inoculation, NIL-R shows reduced CHL degradation (**c**) and less reduction of Fv/Fm value (maximum quantum yield of PSII) (**d**) than NIL-S. Pathogen growth measured with relative expression level of the *ClaTIS* gene via qRT-PCR is transiently repressed on NIL-R at 3 dpi (**e**). Upon *Cor* infection, the relative expression of three CHL catabolic genes, *CsSGR* (**f**), *CsPPH* **(g**), and *CsPAO* (**h**) is significantly up-regulated in NIL-S than in NIL-R at varying dpi while the expression of *CsRCCR* (**i**) shows no difference between the NILs at any time. Bars = mean ± SD (n=8 for B and n=3 for C thru H). ns= not significant; *, *P<0.05*; **, *P<0.01* based on pair-wise t tests.


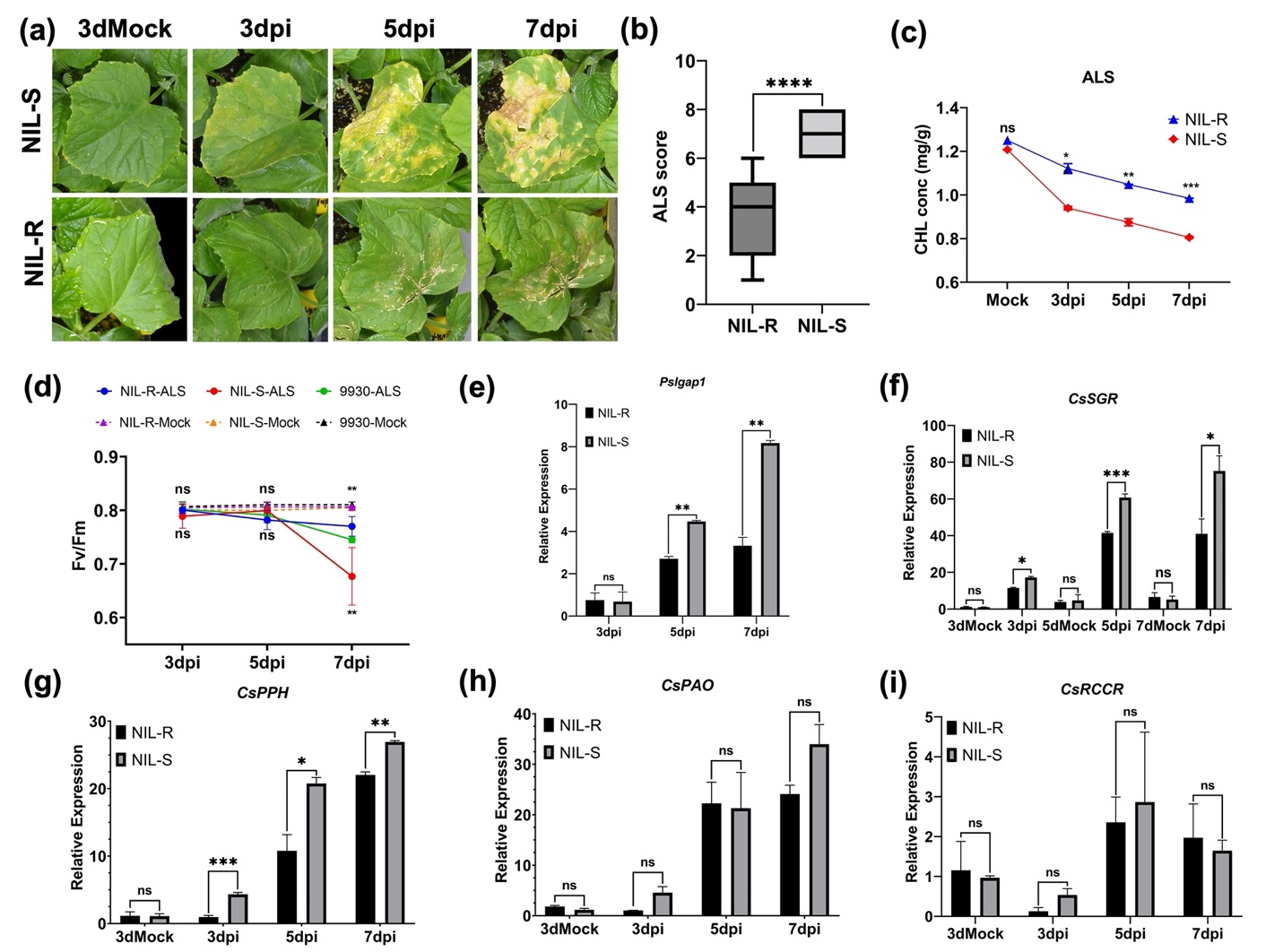


**Fig. S4. Characterization of angular leaf spot (ALS) resistance in near-isogenic resistant (NIL-R) and susceptible (NIL-S) lines for the *dm1/cla/psl* locus.** (**a**) ALS symptom development on the leaves of two NILs at different days post artificial inoculation (dpi) of *P. sringae pv. lachrymans* (*Psl*) under growth chamber conditions. (**b**) Bar graphs of mean ALS disease scores in NIL-R and NIL-S. Upon inoculation, NIL-R shows reduced CHL degradation (**c**) and less reduction of Fv/Fm value (maximum quantum yield of PSII) at 7dpi (**d**) than NIL-S. Pathogen growth measured with relative expression level of the *Pslgap1* gene via qRT-PCR is repressed in NIL-R at 3 and 7dpi (**e**). Upon *Psl* infection, the relative expression of two CHL catabolic genes, *CsSGR* (**f**) and *CsPPH* **(g**), is significantly up-regulated in NIL-S than in NIL-R at varying dpi while the expression of *CsPAO* (**h**) and *CsRCCR* (**i**) show no difference between the NILs at any time. Bars = mean ± SD (n=8 for **b** and n=3 for **c** thru **h**). ns= not significant; *, *P<0.05*; **, *P<0.01* based on pair-wise t tests.


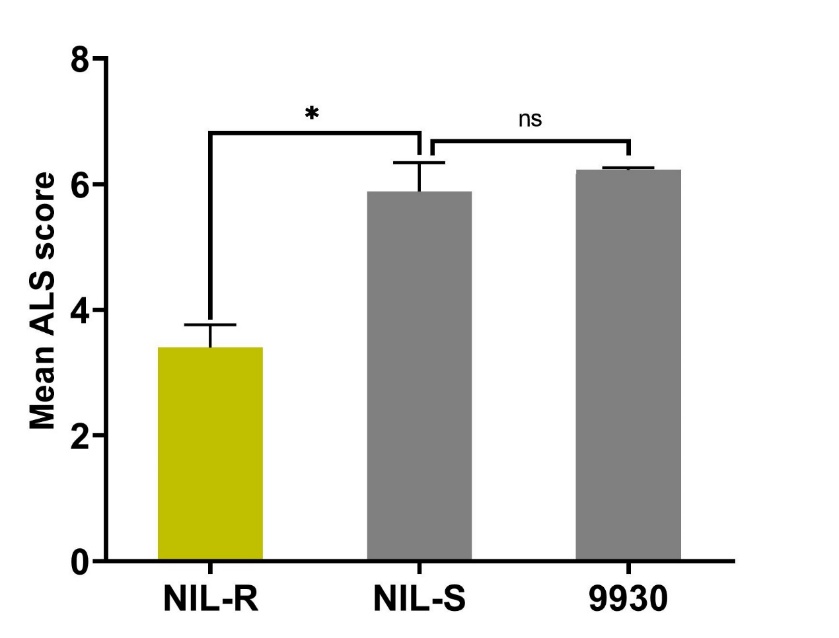


**Fig. S5. Performance of ALS resistance of NIL-S and NIL-R in response to natural *Psl* infection under field conditions.** ALS mean disease scores were from 2021 and 2022 field trials at Hancock, WI. ALS disease scores were evaluated on at least 10 plants for each genotype per year. T-tests were performed to test if there is a significant difference in mean ALS scores of NIL-R or 9930 compared with NIL-S. *, *P<0.05*; ns=not significant.

**
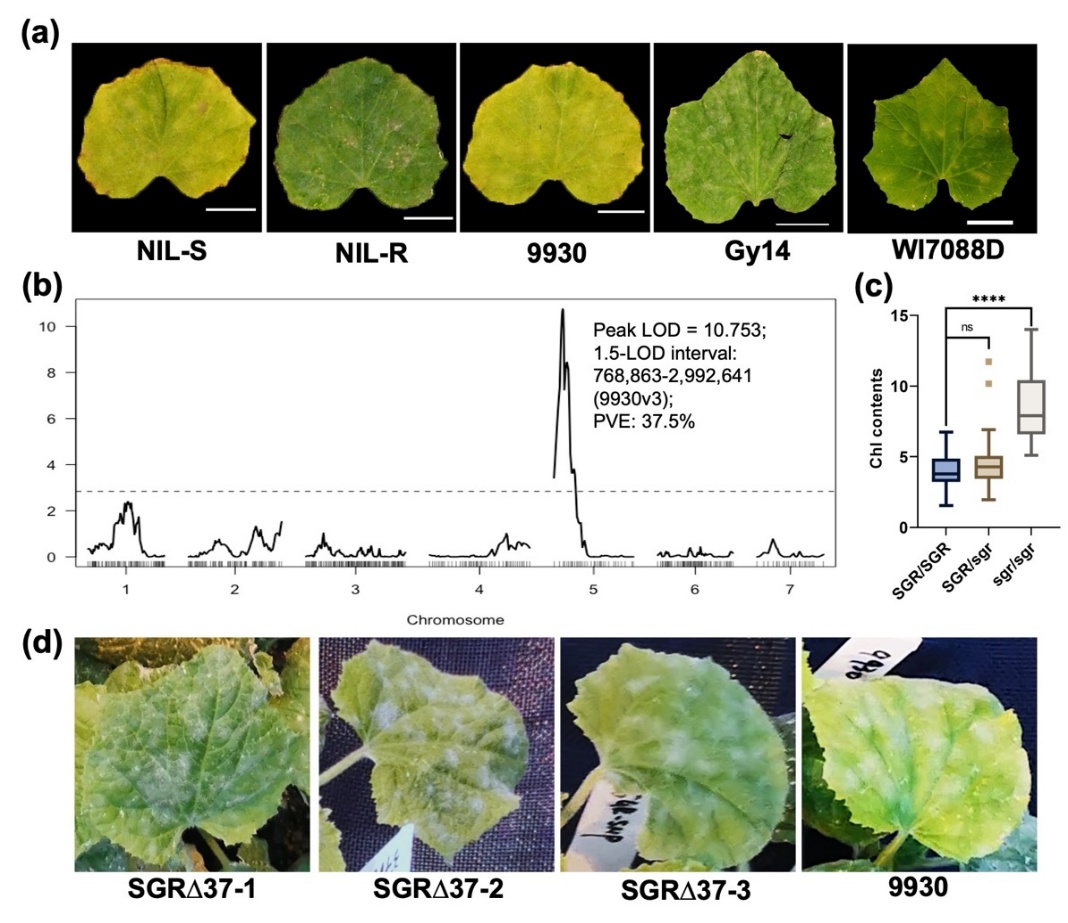
**

**Fig. S6 Performance of powdery mildew (PM) resistance of NILs and QTL analysis of PM resistance in Gy14 cucumber.** (**a**) Under greenhouse conditions, PM infection results in chlorosis or yellowing in susceptible 9930 and NIL-S plants while NIL-R, Gy14, and WI7088D stay green. (**b**) QTL mapping for the anti-chlorosis effect upon PM pathogen infection in the Gy14 × 9930 RIL population with phenotypic data from 2022 greenhouse trials. The horizontal dashed line indicates LOD threshold for significant QTL. Information for the major-effect QTL including peak LOD support value, phenotypic variance explained (PVE), and physical interval of this locus is shown, which is co-localized with the *CsSGR* locus. (**c**) Boxplot showing segregation in chlorophyll contents in PM-infected leaves of a NIL-derived F_2_ population is significantly associated with *CsSGR* alleles through the Kruskal-Wallis test (*P<=0.0001*). (**d**) Performance of PM resistance of three *CsSGR* gene-edited lines under greenhouse conditions showing the anti-chlorosis effect of SGRΔ37 mutations on *Px* infection.


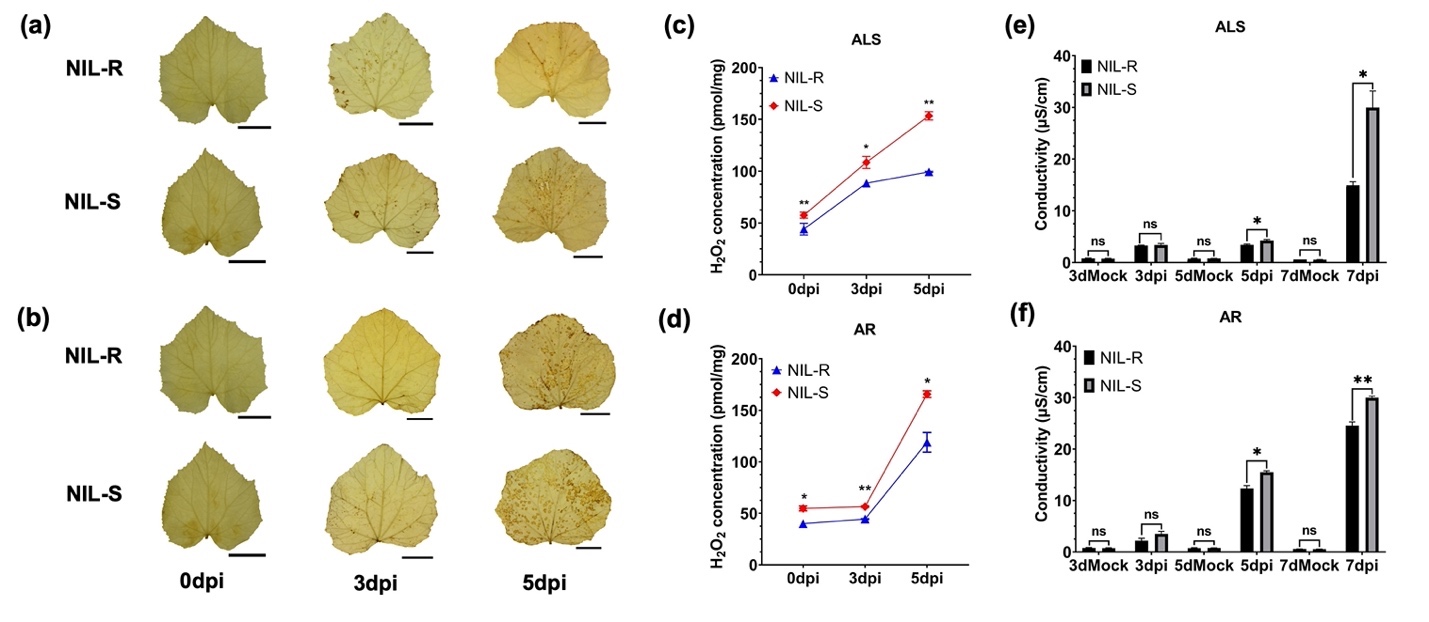


**Fig. S7 H_2_O_2_ and cell death induced by infection of pathogens is mitigated in NIL-R. a-b**, H_2_O_2_ was detected by DAB staining in NIL-R and S post inoculation of pathogens of AR and ALS, respectively; **c-d** display dynamics of H_2_O_2_ concentration in NIL-R and S post inoculation of pathogens of AR and ALS, respectively; **e-f** display cell death estimated by conductivity in NIL-R and S without or post inoculation of AR, and ALS, respectively. Bars indicate SD (n=3). ns = not significant; *, *P < 0.05*; **, *P < 0.01* from pair-wise t tests.


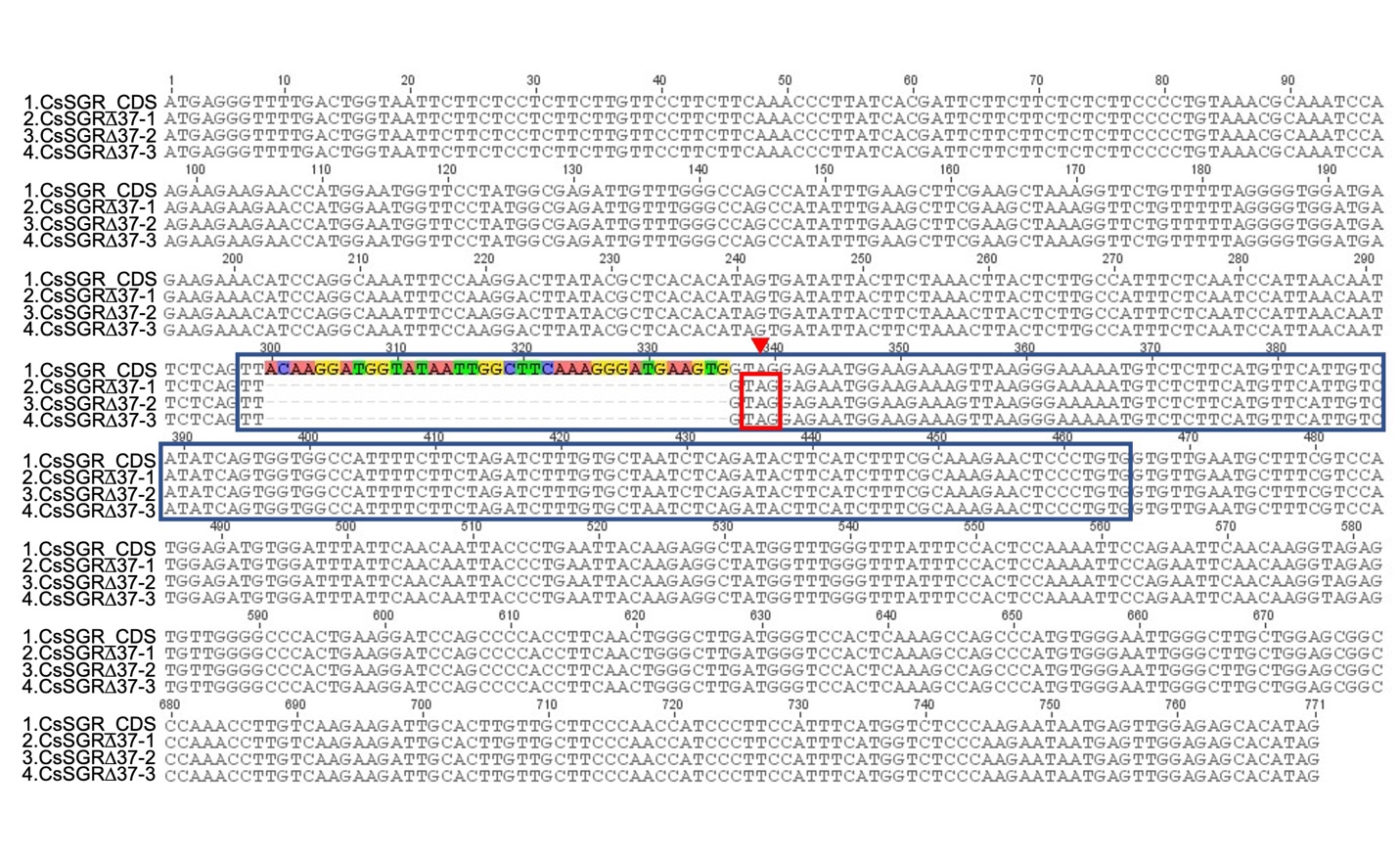


**Fig. S8 Alignment of *CsSGR* coding region sequences from *CsSGR*-KO lines and WT.** The rectangular box in blue color highlights the sequence of the 3^rd^ exon of *CsSGR*. The red box and triangle highlight a premature terminator for *CsSGRΔ37*.


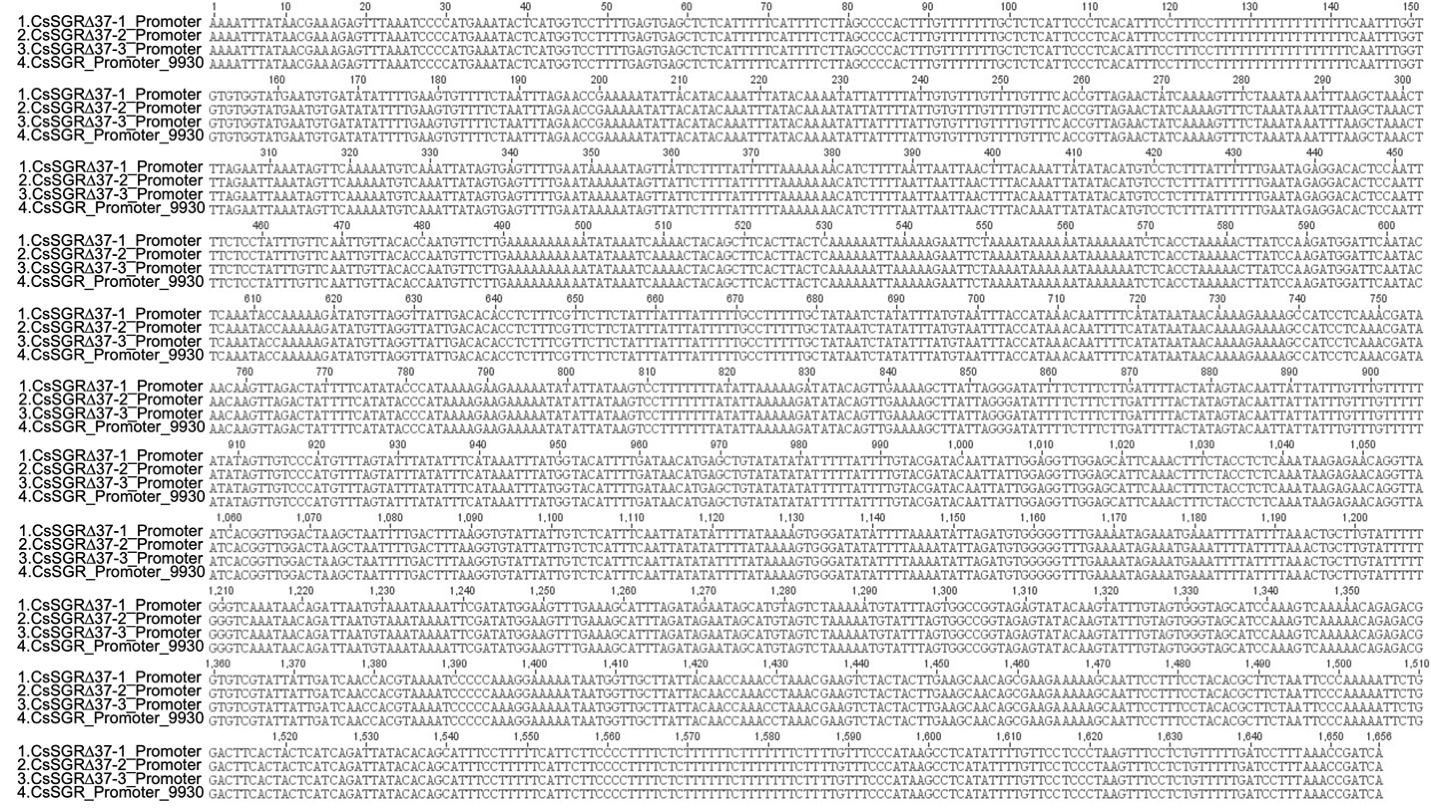


**Fig. S9 Alignment of *CsSGR* promoter sequences from *CsSGR*-KO lines and WT.**

**
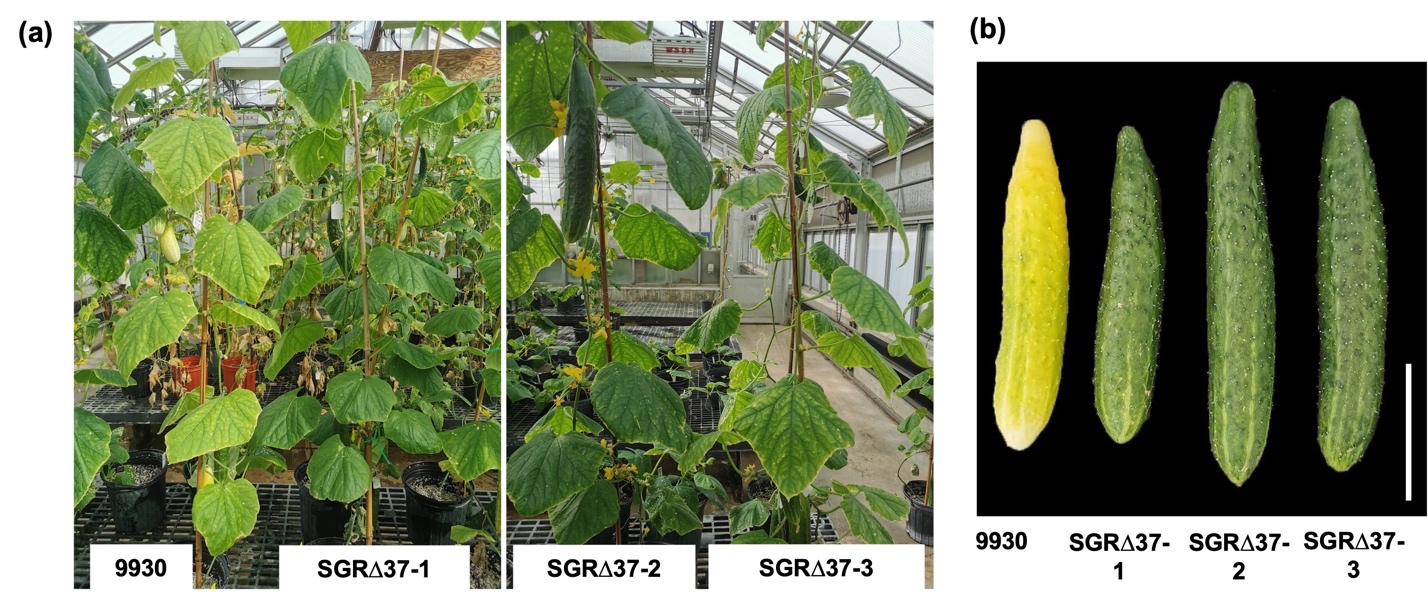
**

**Fig. S10 The stay-green effect observed from *CsSGR* knock-out plants on plant senescence (a) and mature fruit skin color (b).** Pictures were taken of plants at 75 days post-germination and mature fruits at 32 days post-pollination.

**
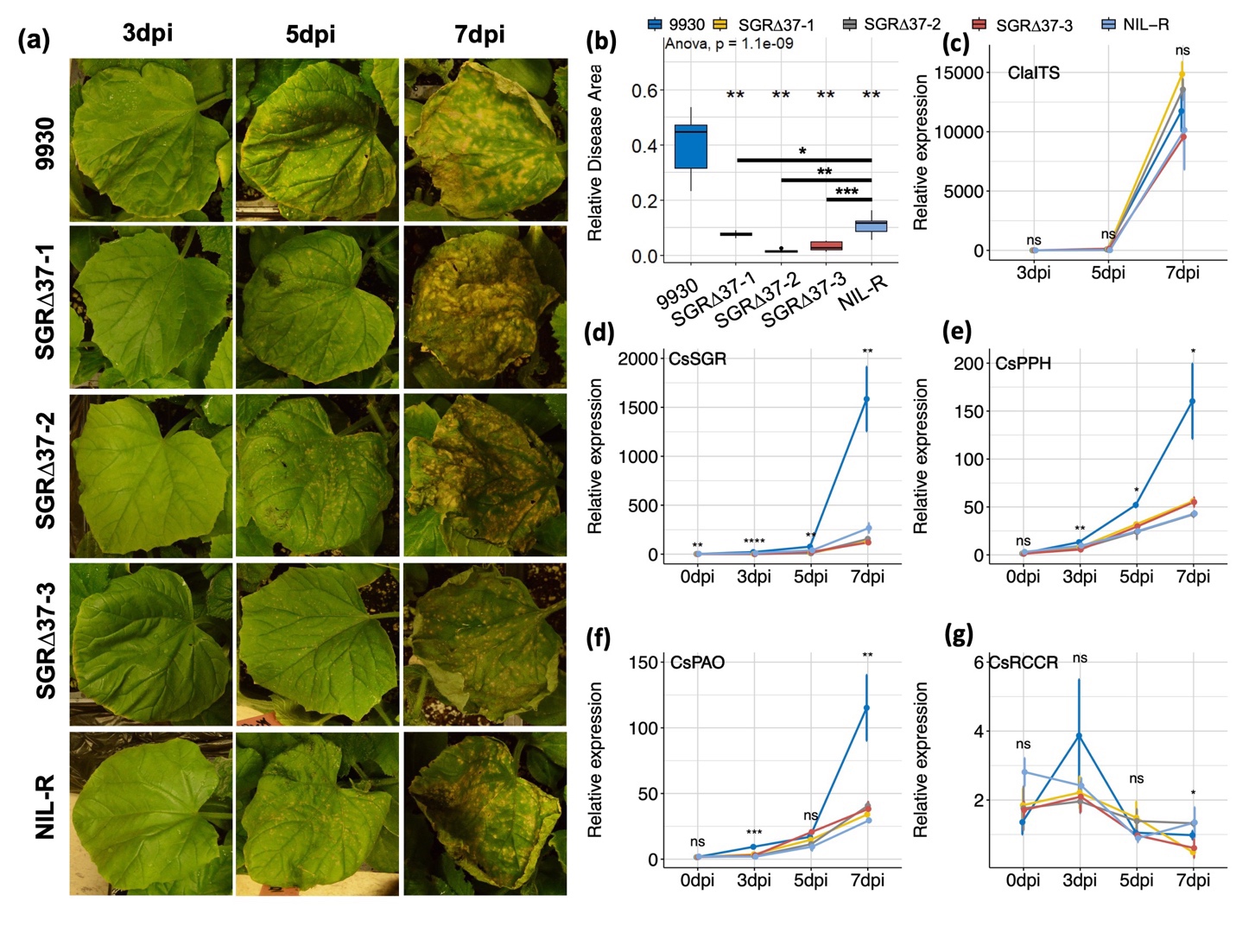
**

**Fig. S11 Evaluation of AR response for *CsSGR* knock-out plants.** (**a**) Anthracnose responses on the upper (adaxial) leaf surface inoculated with 1×10^6^ conidia/ml *C. orbicular*e during a time course (3dpi-7dpi). Cucumber seedlings were grown in growth chamber with temperature of 24°C(day)/20°C(night) and photoperiod 16h/8h(night). (**b**) A boxplot displaying AR disease severity across genotypes. AR disease severity was determined by relative disease area precisely measured by ImageJ software. At least five individuals per genotype were used in scoring. The inoculation was performed through the method of spray. ANOVA test was initially performed to test if there is a significant difference among all genotypes, and t-tests were used to compare the AR severity of each genotype with 9930. Additionally, nonparametric tests were applied to test if the AR severity of three gene-edited mutants is significantly different from NIL-R. Quantification of *C. orbicular*e (**c**) and relative expression levels of *CsCCGs* (**d-g**) in NIL-R and S post inoculation of 1×10^6^ conidia/ml *C. orbicular*e using qRT-PCR analysis. The qRT-PCR analysis was performed on three biological replicates with three technical replicates each. DNA primers specific for *C. orbicular*e internal transcribed space normalized to *Cucumis sativus* actin. Relative expression was calculated where relative expression = 2^(−∆∆Ct)^. Bars indicate SD (n=3). Ns= not significant; *, **, ***, and **** indicate *P<0.05,* *0.01, 0.001, and* 0.0001, respectively.


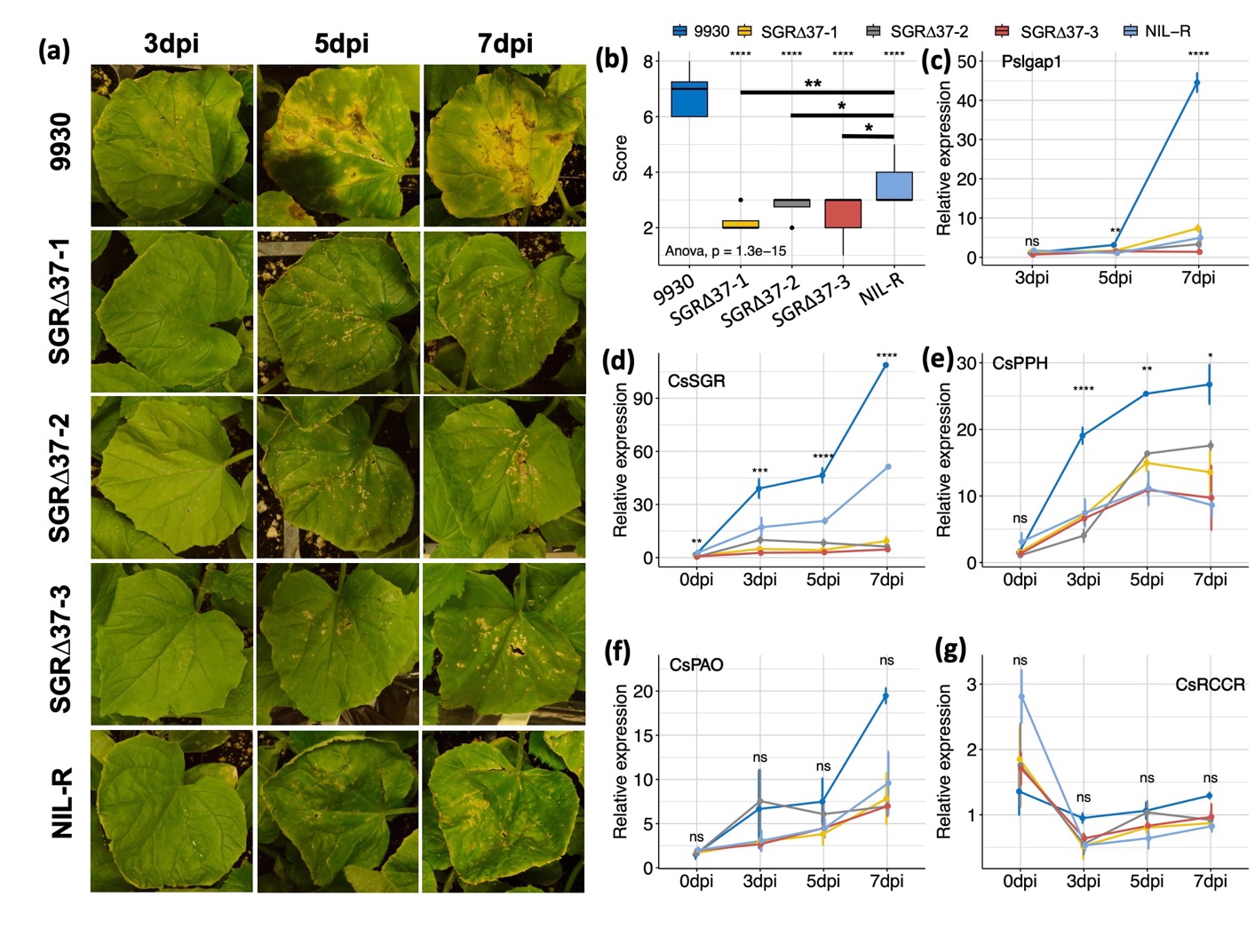


**Fig. S12 Evaluation of ALS response for *CsSGR* knock-out plants.** (**a**) Angular leaf spot responses on the lower (abaxial) leaf surface inoculated with 5×10^6^ CFU/ml *Psl* during a time course (3dpi-5dpi). Cucumber seedlings were grown in growth chamber with temperature of 24°C(day)/20°C(night) and photoperiod 16h/8h(night). The inoculation was performed through the method of spray. The inoculated seedlings were kept in darkness for 24h under plastic domes (100% relative humidity). (**b**) A boxplot displaying ALS disease scores across genotypes. At least five individuals per genotype were used in scoring. ANOVA test was initially performed to test if there is a significant difference among all genotypes, and t-tests were used to compare the mean ALS scores of each genotype with 9930. Additionally, nonparametric tests were applied to test if the ALS scores of three gene-edited mutants are significantly different from NIL-R. Quantification of *Psl* growth (**c**) and relative expression levels of *CsCCGs* (**d-g**) in NIL-R and S post inoculation of 5 × 10^6^ CFU/ml *Psl* spores with qRT-PCR analysis. DNA primers specific for *P. sringae pv. lachrymans gap1* normalized to *Cucumis sativus* actin. Relative expression was calculated where relative expression = 2^(−∆∆Ct)^. Each datapoint is mean ± SD (n=3). ns= not significant; *, **, ***, and **** indicate *P<0.05,* *0.01, 0.001, and* 0.0001, respectively.

**
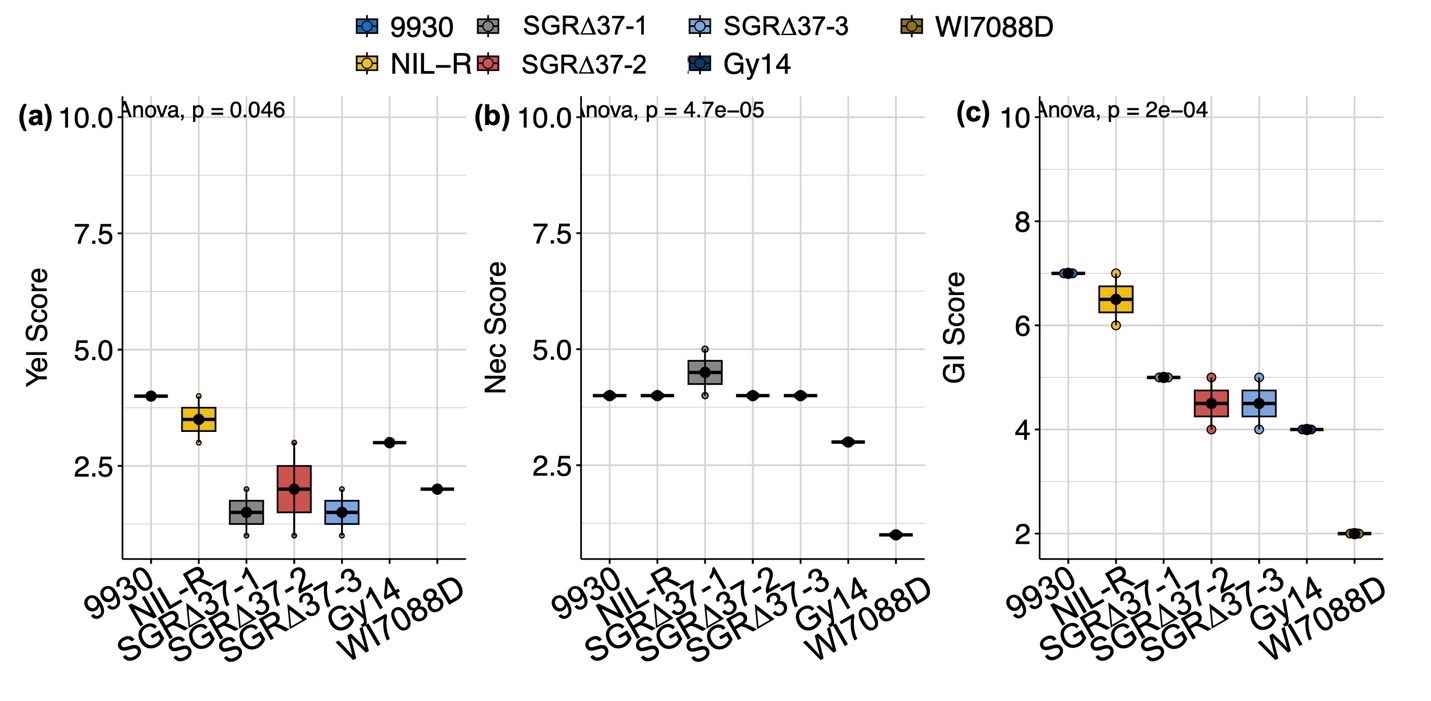
**

**Fig. S13 Evaluation of downy mildew responses of *CsSGR* knock-out lines in open field.** (**a**) DM yellowing score; (**b**) DM necrosis score; (**c**) DM general impression score. ANOVA test was performed to test if there is a significant difference among genotypes.


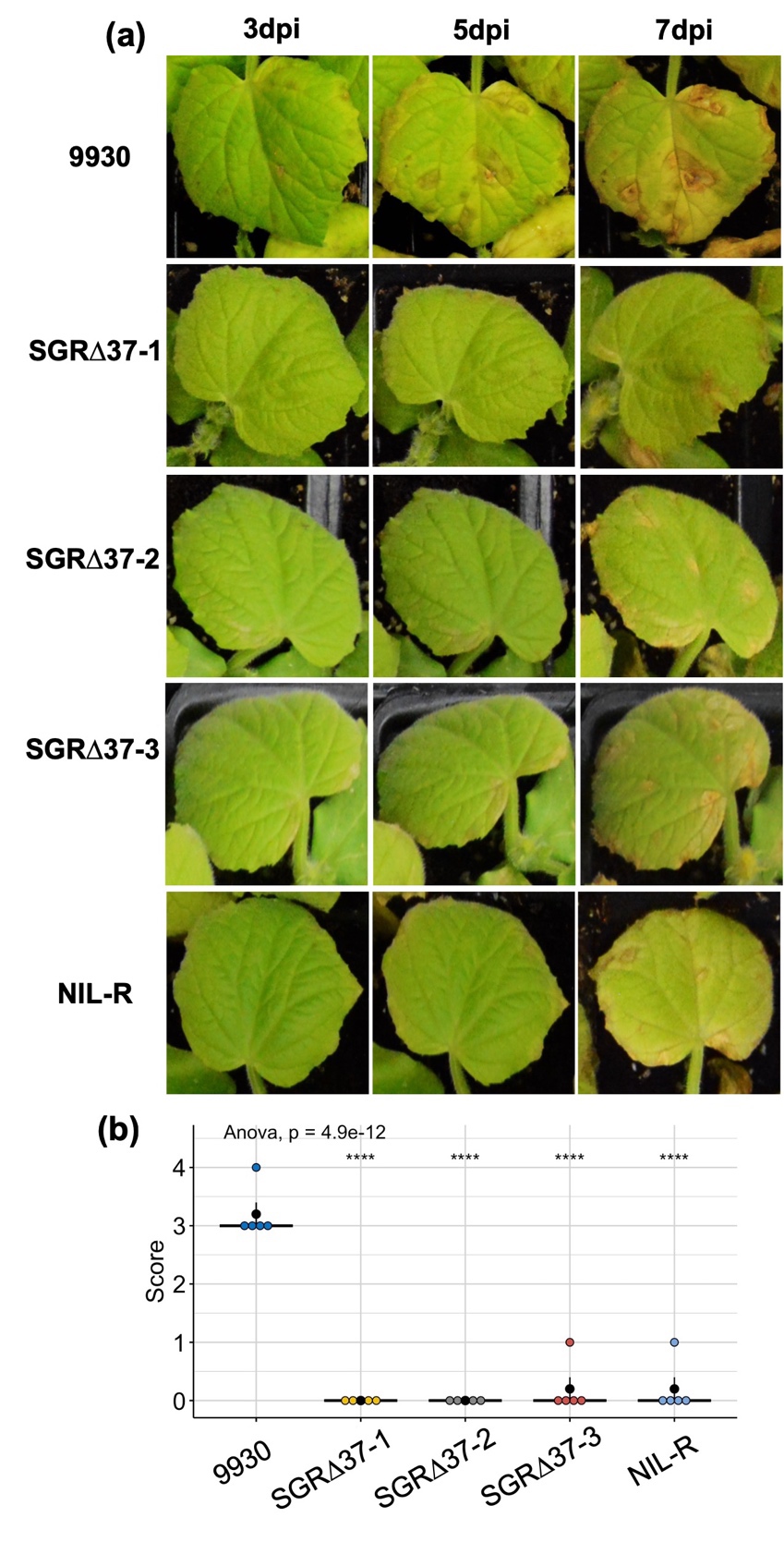


**Fig. S14 Evaluation of TLS response for *CsSGR* knock-out plants**. (**a**) TLS responses of different genotypes at 7dpi; (**b**) TLS scores of each genotype. Disease scores were evaluated using five ratings based on percentages of disease area on leaves. 0: 0-5%, 1 = 5-10%, 2 = 21-50%, 4 = 71-100% of disease areas or dead. Five individuals per genotype were used in scoring and 9930 was used as the reference in the t-test. *, *P < 0.05; **, P < 0.01; ***, P < 0.001; ****, P < 0.0001.*

*
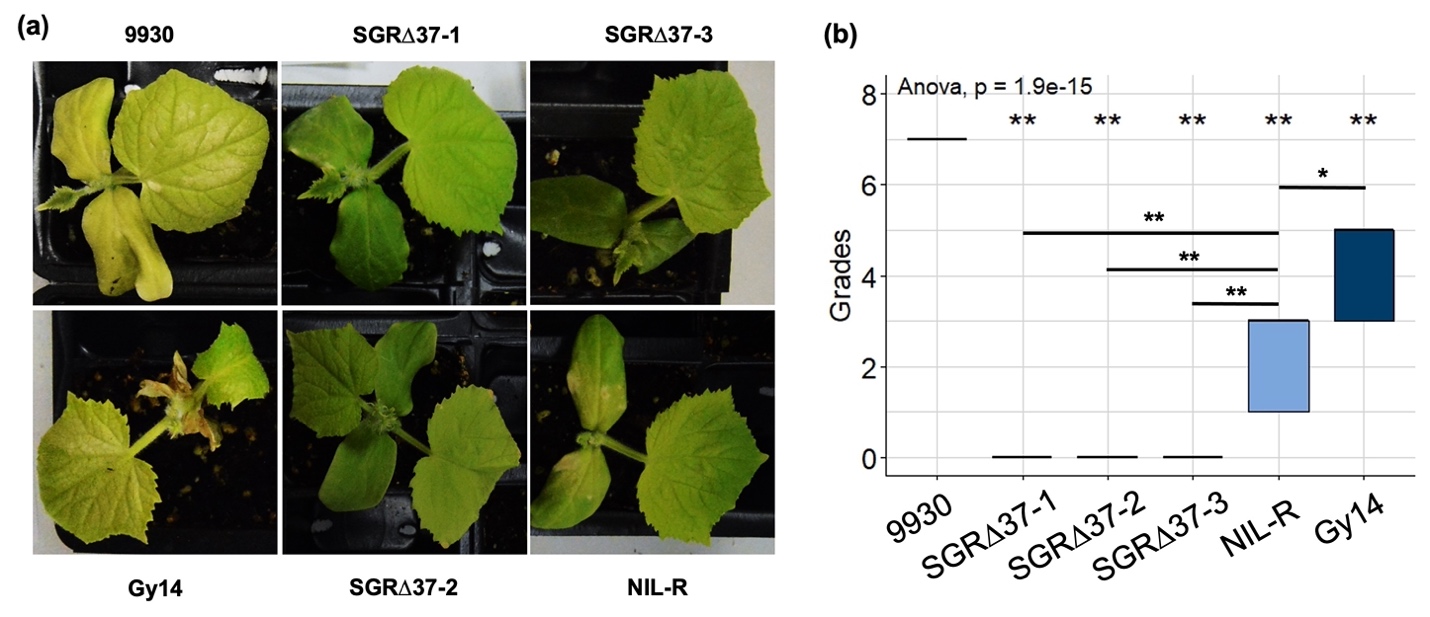
*

**Fig. S15 Evaluation of cold stress responses among *CsSGR* knock-out plants.** (**a**) Responses of different genotypes post 2-week low-temperature treatment; (**b**) Grades of low-temperature tolerance (LT) of each genotype. LT responses were quantified based on the six grades (Grade 0, 1, 3, 5, 7, 9) (Dong et al., 2019). Five individuals per genotype were used in grading and 9930 was used as the reference in the Wilcox-test. *** P < 0.01.* Additionally, nonparametric tests were applied to test if the LT grades of NIL-R were significantly different from the grades of three knock-out mutants or Gy14.

**
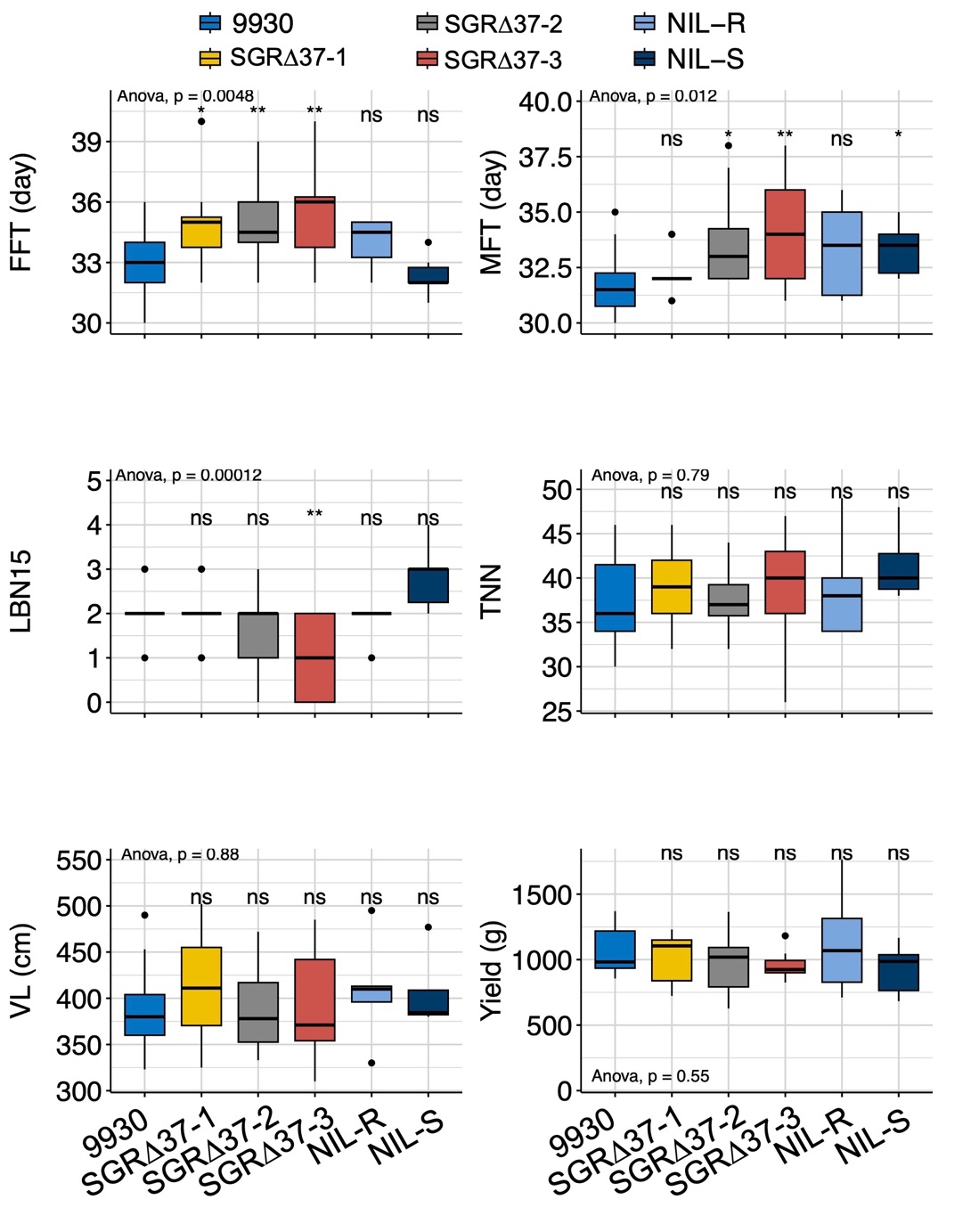
**

**Fig. S16 Evaluation of horticultural traits for *CsSGR* knock-out, WT, and NIL-R and NIL-S plants in greenhouse trials.** FFT, female flowering time; MFT, male flowering time; LBN15, lateral branch number within the first 15^th^ nodes; TNN, total number of nodes; VL, vine length; Yield, total parthenocarpic fruit weight per plant. For gene-editing lines and WT, n = 12; for NIL-R, n = 6. ns = not significant; *, *P* < 0.05; **, *P* < 0.01.


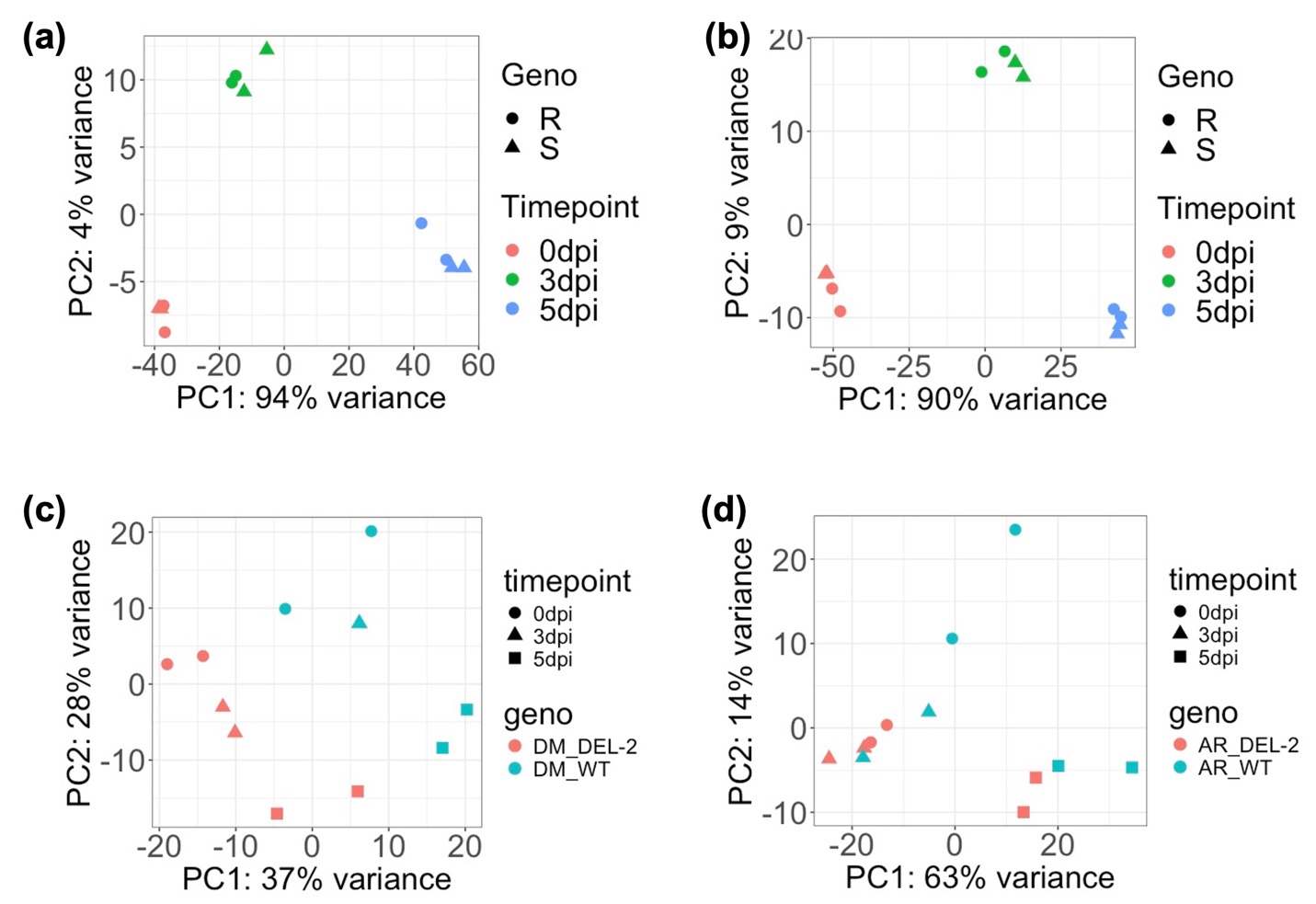


**Fig. S17 PCA analysis of RNA-seq data in this study.** (**a**) DM NILs RNA-Seq data; (**b**) AR NILs RNA-seq data; (**c**) RNA-seq data of WT (9930) and SGRΔ37-2 knock-out plants before or after DM pathogen infection; (**d**) RNA-seq data of WT (9930) and SGRΔ37-2 knock-out plants before or after AR pathogen infection. R = NIL-R; S = NIL-S.


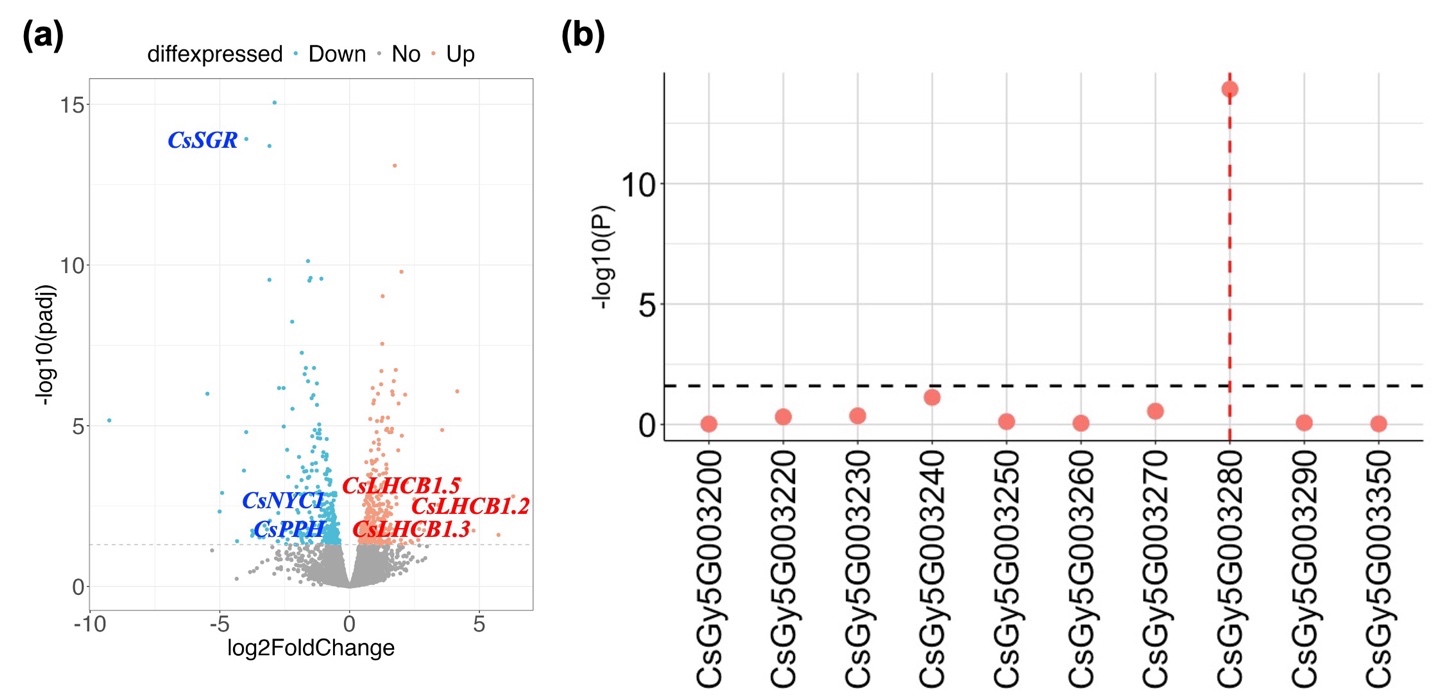


**Fig. S18 Analysis of constitutively and differentially expressed genes in SGRΔ37-2 KO plants without pathogen infection.** (**a**) Volcano plots of DEGs in SGRΔ37-2 mutant vs WT (9930) transcriptomes at 0 dpi. Orange and blue dots represent significant up-regulated or down-regulated DEGs in the SGRΔ37-2 KO plant. Selected down-regulated *CCGs* and up-regulated *CsLHCB1* genes are highlighted in blue and red color, respectively. (**b**) Within 100kb region surrounding the *CsSGR* locus with 10 annotated genes, *CsSGR* is the only DEG between the SGRΔ37-2 mutant and 9930 WT. Each dot represents one gene. *CsGy5G003200*, SPX domain-containing membrane protein; *CsGy5G003220*, Molybdopterin cofactor sulfurase, putative; *CsGy5G003230*, Unknown Protein; *CsGy5G003240*, bifunctional dTDP-4-dehydrorhamnose 3,5-epimerase/dTDP-4-dehydrorhamnose reductase; *CsGy5G003250*, homeobox protein knotted-1-like 7; *CsGy5G003260*, ubiquitin carboxyl-terminal hydrolase MINDY-2-like; *CsGy5G003270*, MADS-box transcription factor 50; *CsGy5G003280*, protein STAY-GREEN, chloroplastic-like; *CsGy5G003290*, Guanine nucleotide-binding protein subunit beta-like protein; *CsGy5G003350*, Subtilisin-like protease.


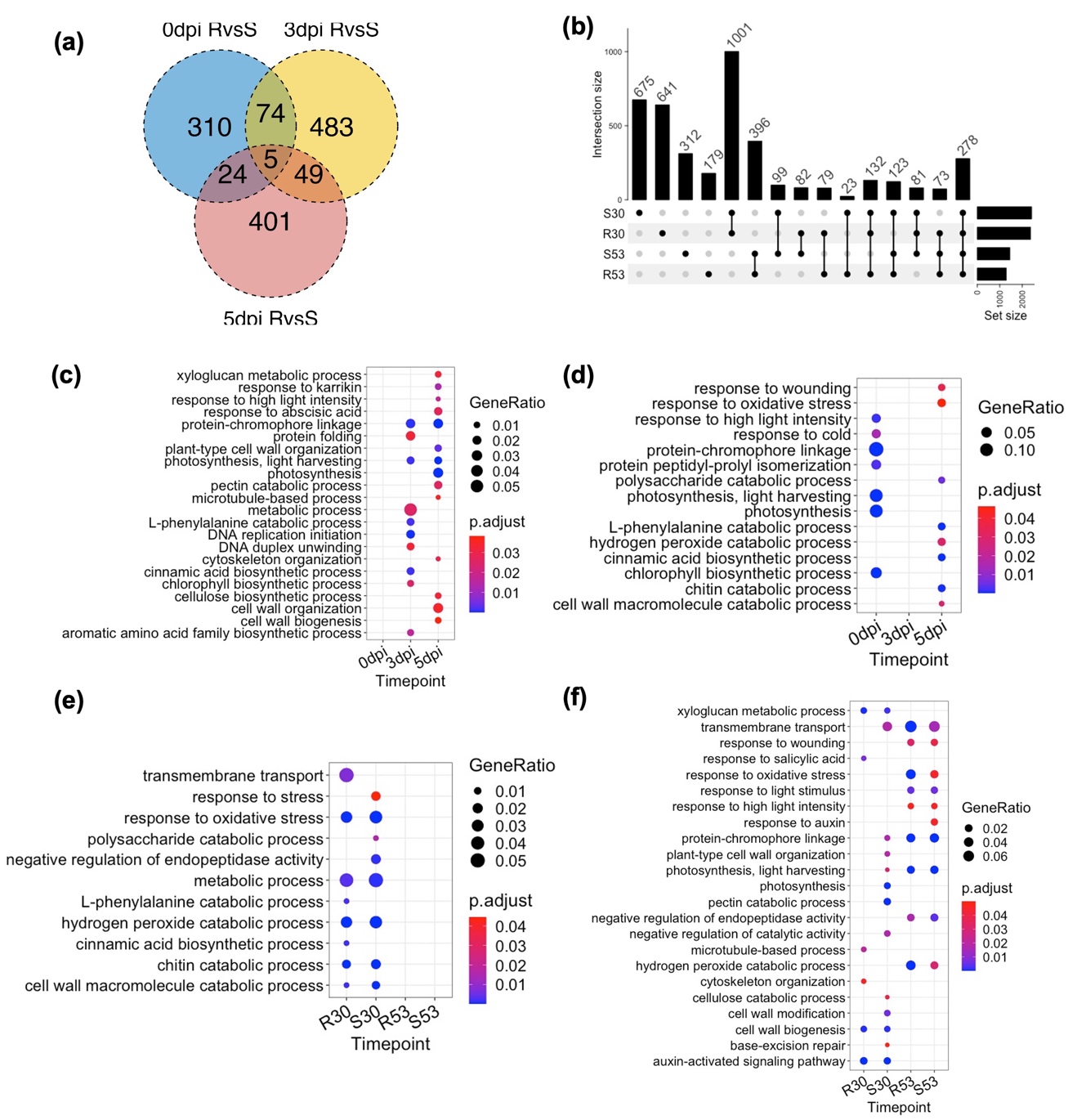


**Fig. S19 Analysis of differentially expressed genes (DEGs) in NIL-R and NIL-S in response to artificial inoculation of the AR pathogen (*Cor*).** (**a**) Venn diagram of numbers of DEGs at 0, 3, and 5 dpi in comparisons of NIL-R vs NIL-S. (**b**) UpSet plot showing shared DEGs from comparisons of transcriptomes in and between two NILs at different time points post *Cor* inoculation. (**c**) and (**d**) are top enriched Biological Process (BP) GO terms for up- and down-regulated DEGs in NIL-R vs NIL-S comparisons at 0, 3, and 5 dpi, respectively. (**e)** and (**f**) are top enriched BP GO terms of up- and down-regulated DEGs from comparisons between 3 vs 0 and 5 vs 3 dpi in two NILs, respectively.


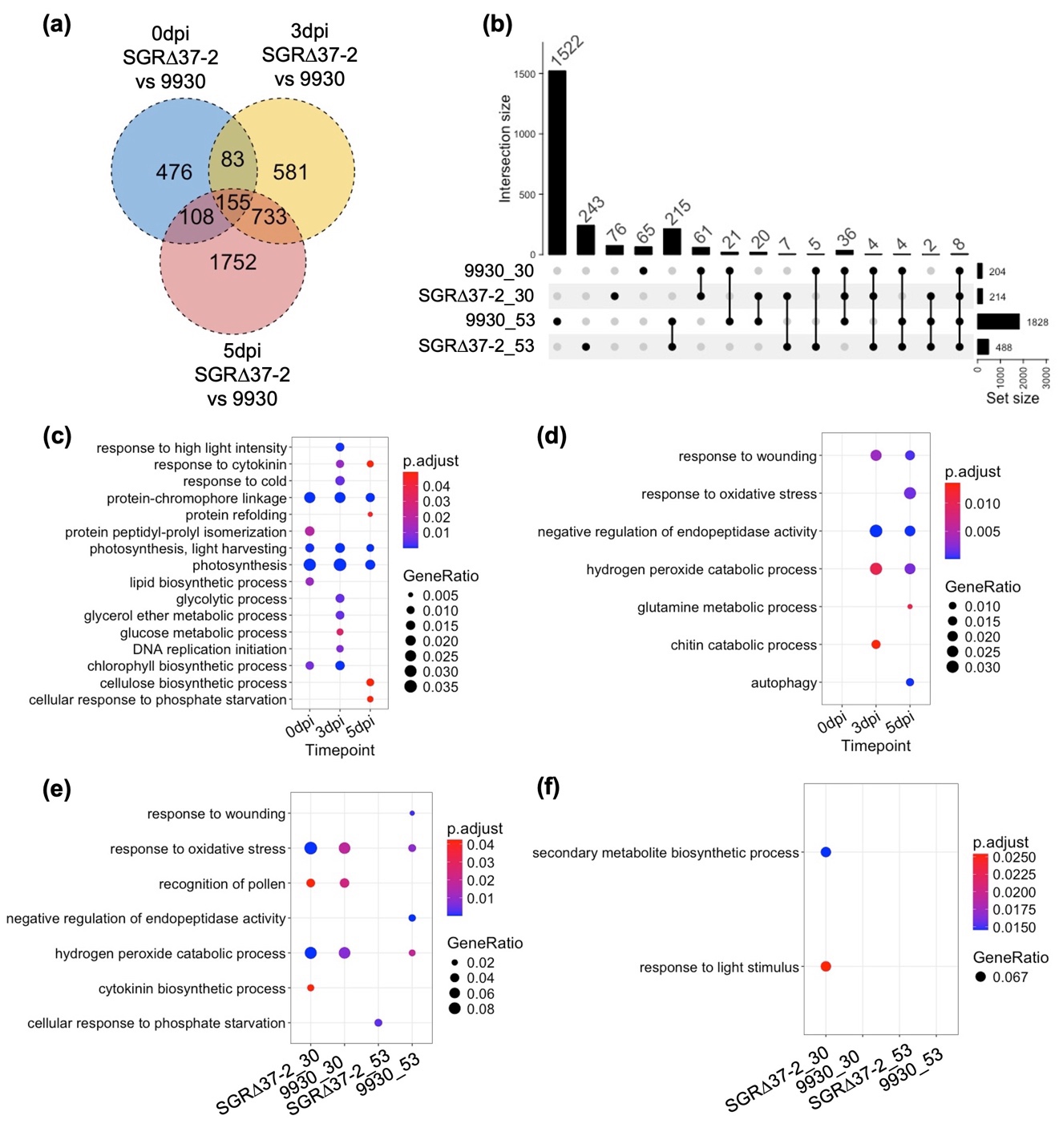


**Fig. S20 Analysis of differentially expressed genes (DEGs) in SGRΔ37-2 and 9930 in response to artificial inoculation of the DM pathogen, *P. cubensis*.** (**a**) Venn diagram of numbers of DEGs at 0, 3, and 5 dpi in comparisons of SGRΔ37-2 vs 9930. (**b**) UpSet plot showing shared DEGs from comparisons of transcriptomes in and between SGRΔ37-2 and 9930 at different time points post *Pcu* inoculation. (**c**) and (**d**) are top enriched Biological Process (BP) GO terms for up- and down-regulated DEGs in SGRΔ37-2 vs 9930 comparisons at 0, 3, and 5 dpi, respectively. (**e)** and (**f**) are top enriched BP GO terms of up- and down-regulated DEGs from comparisons between 3 vs 0 and 5 vs 3 dpi in SGRΔ37-2 and 9930, respectively.

**
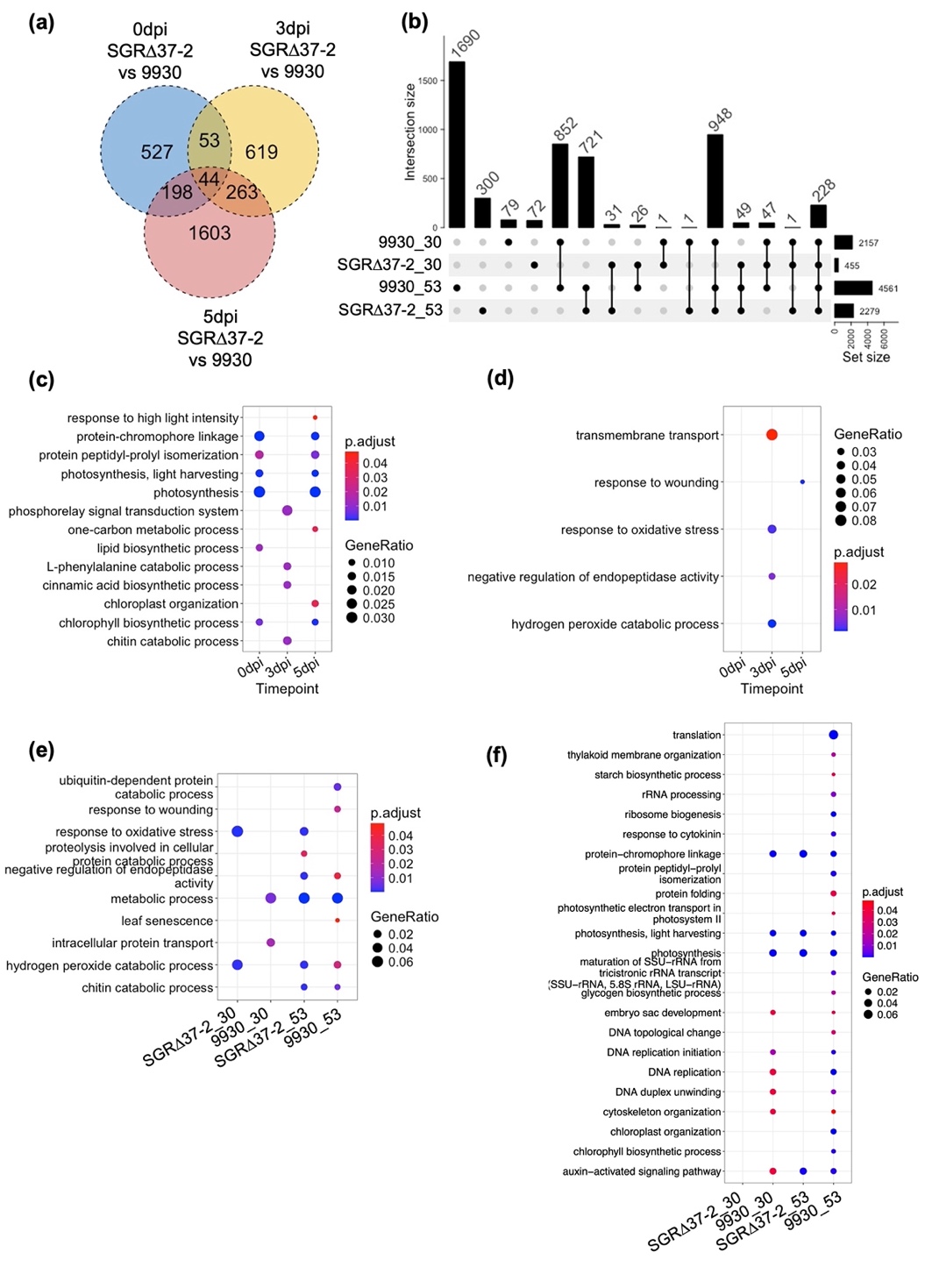
**

**Fig. S21 Analysis of differentially expressed genes (DEGs) in SGRΔ37-2 and 9930 in response to artificial inoculation of the AR pathogen (*Cor*).** (**a**) Venn diagram of numbers of DEGs at 0, 3, and 5 dpi in comparisons of SGRΔ37-2 vs 9930. (**b**) UpSet plot showing shared DEGs from comparisons of transcriptomes in and between SGRΔ37-2 and 9930 at different time points post *Cor* inoculation. (**c**) and (**d**) are top enriched Biological Process (BP) GO terms for up- and down-regulated DEGs in SGRΔ37-2 vs 9930 comparisons at 0, 3, and 5 dpi, respectively. (**e)** and (**f**) are top enriched BP GO terms of up- and down-regulated DEGs from comparisons between 3 vs 0 and 5 vs 3 dpi in SGRΔ37-2 and 9930, respectively.


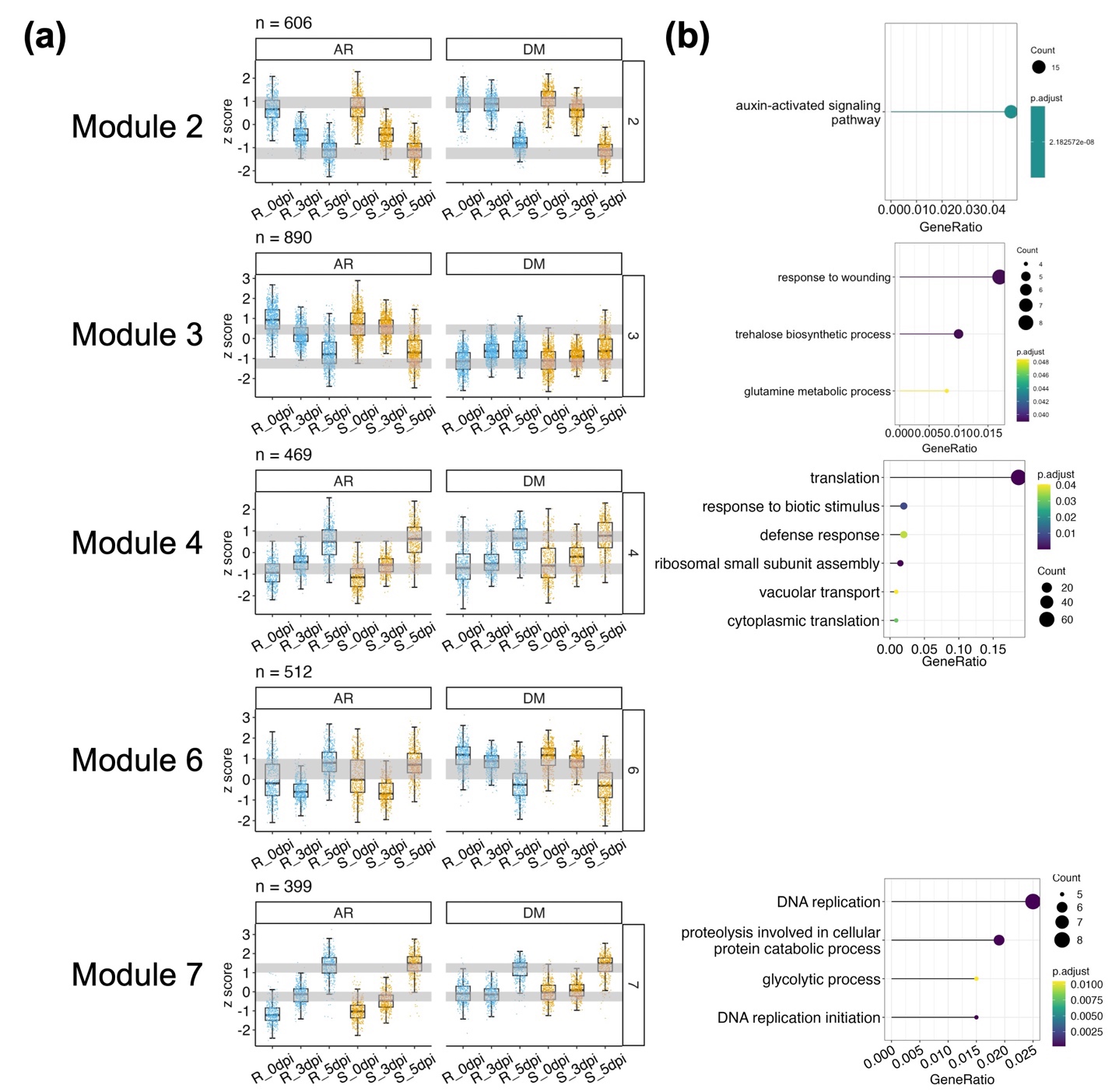


**Fig. S22 Co-expression modules identified from gene co-expression analysis of transcriptomes of NIL-R and NIL-S in response to *Pcu* and *Cor* infection.** (**a**) Barplot visualization of three gene co-expression modules in response to the inoculation of the DM (*Pcu*) and AR (*Cor*) pathogens. (**b**) Top enriched GO terms of biological processes in each co-expression module. (Figure continues on next page)


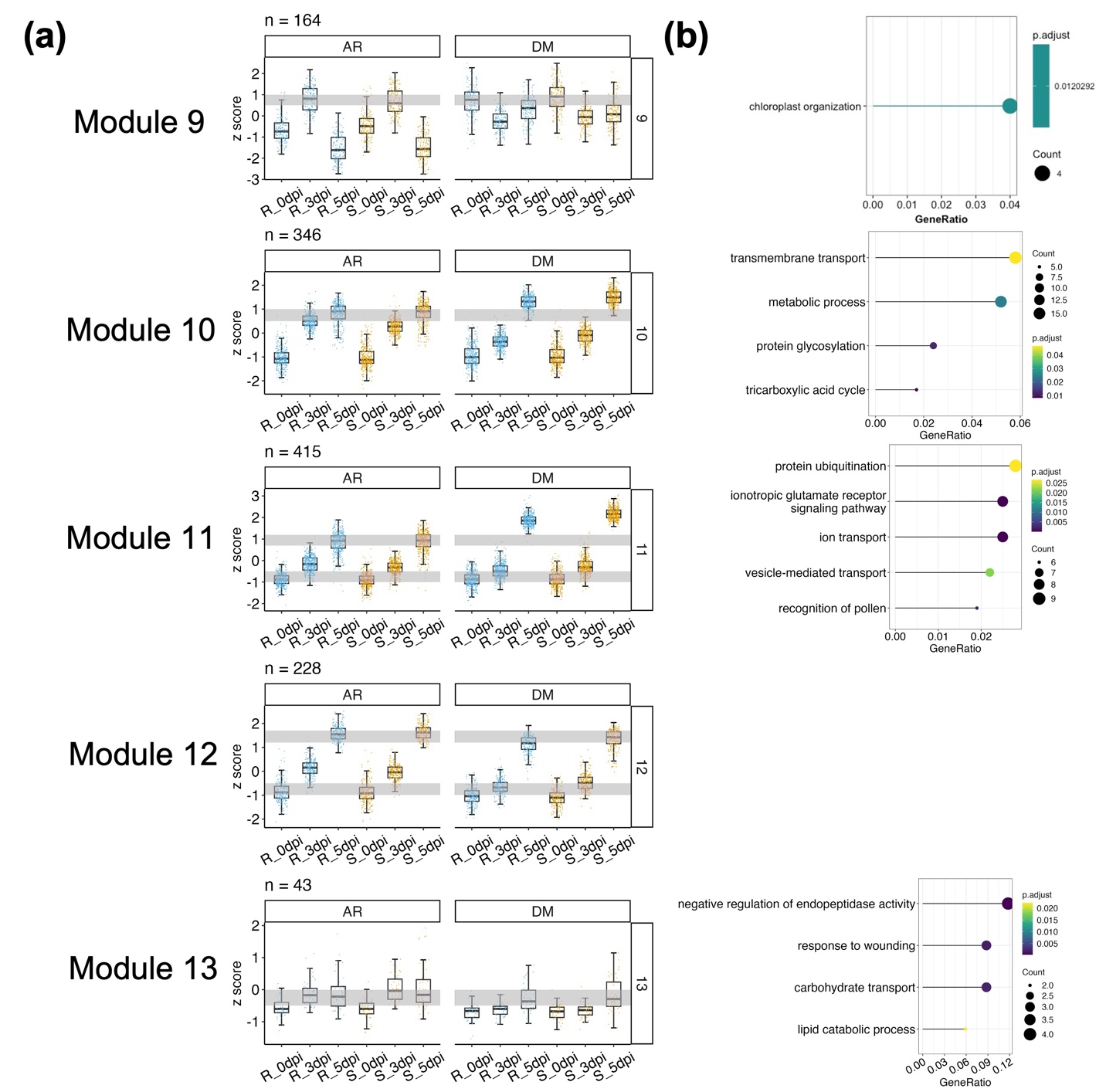


(Continued)


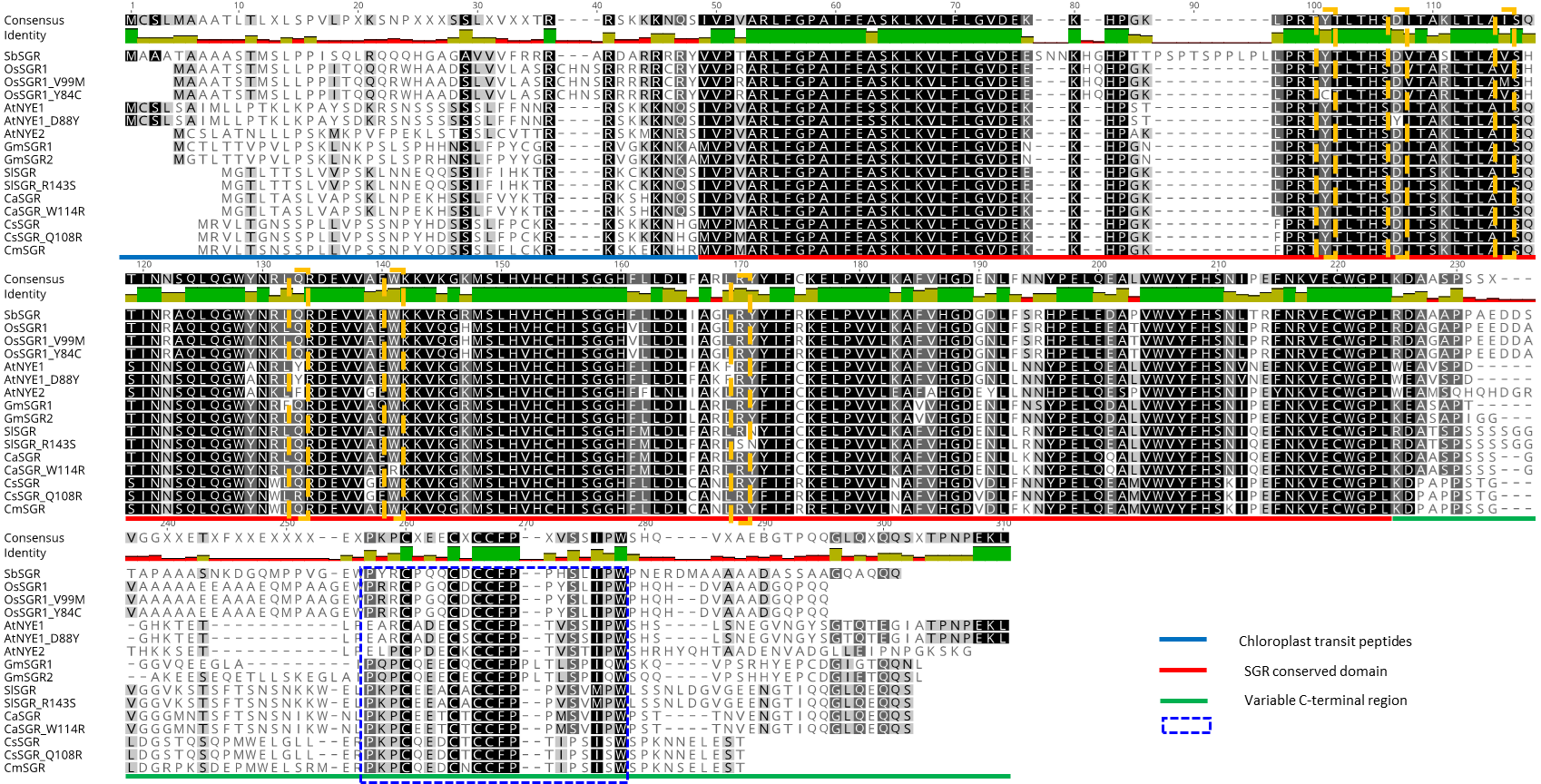


**Fig. S23 Domain structure of SGR homologs.** Blue, red, and green lines denote the chloroplast transit peptide domain, the conserved SGR domain, and the variable C-terminal region. Yellow dashed tangle denotes five critical point mutations that have been identified in the SGR conserved domain from different plant species, including Y84C and V99M in rice, D88Y in Arabidopsis, R143S in tomato, W114R in pepper, and Q108R in cucumber.


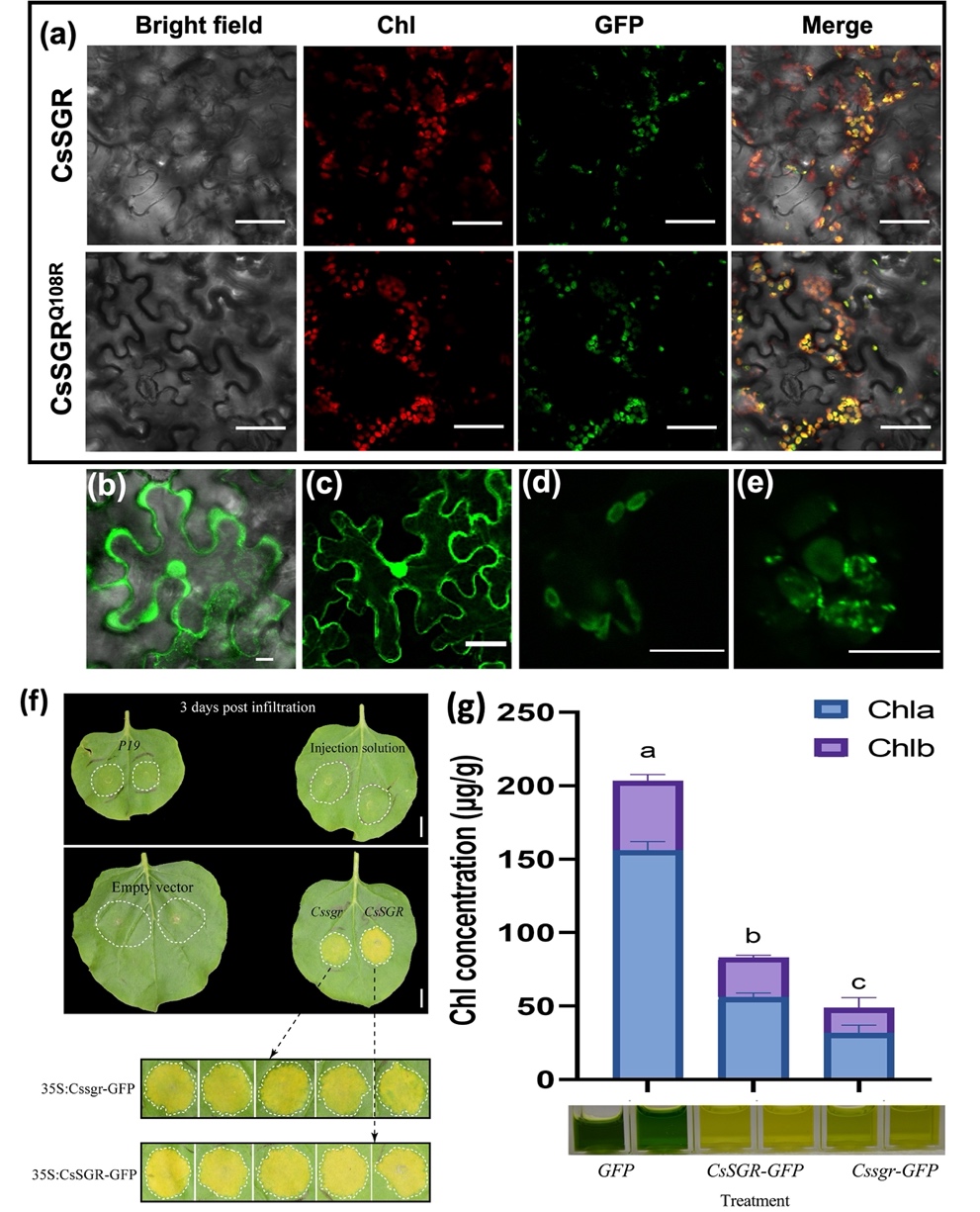


**Fig. S24 Transient overexpression of CsSGR and CsSGR^Q108R^ through *Agrobacterium* infiltration in *N. benthamiana*.** (**a-e**) CsSGR/CsSGR^Q108R^ is localized in chloroplast. GFP, GFP fluorescence; Chl autofluorescence. Bright field, white light; Merge, overlay of all three channels. GFP fluorescence in plants infiltrated with p1307:CsMV35S:GFP (**b**) and p1307:CsMV35S: CsSGRΔN-GFP(**c**) and p1307:CsMV35S: CsSGR-GFP(**d**) and p1307:CsMV35S: Cssgr-GFP(**e**). Scale bar represents 50 μm in **a** and represents 10 μm in **b-e**. Note: Cssgr is CsSGR^Q108R^. (**f**) Visualization of chlorosis of the injection area in each replicate; Upper left: tobacco leaf infiltrated with p19 plasmid; Upper right: tobacco leaf infiltrated with injection buffer; Lower left: tobacco leaf infiltrated with empty vector (35S:GFP); Lower right: tobacco leaf infiltrated with 35S:CsSGR^Q108R^-GFP and 35S:CsSGR-GFP, respectively; (**g**) Chlorophyll extraction and concentration in tobacco leaves infiltrated with 35S-GFP, CsSGR-GFP, and CsSGR^Q108R^-GFP, respectively. Each infiltration was performed on three independent biological replicates. Tukey’s test was performed to test if there is a significant difference among genotypes. Means that do not share a letter are significantly different.

**
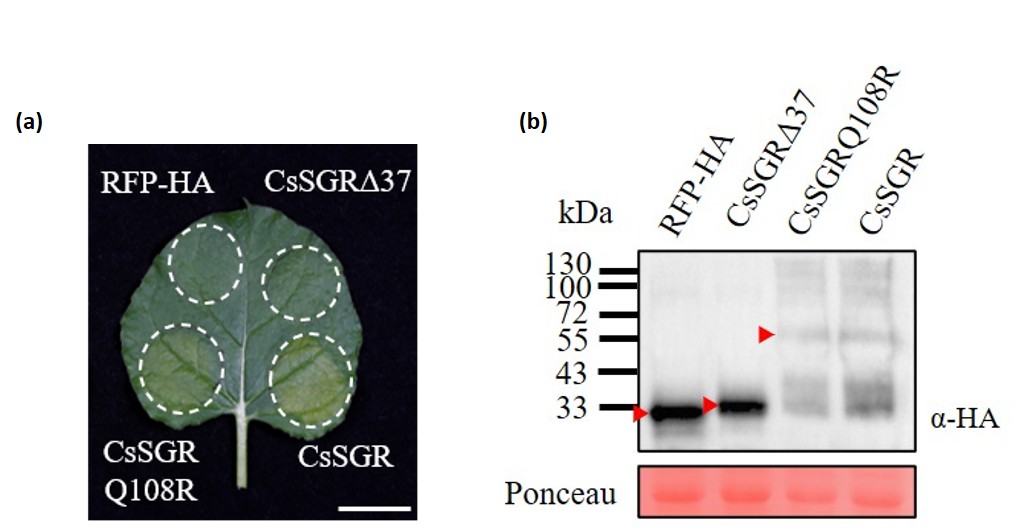
**

**Fig. S25 Western blot analysis of CsSGR, CsSGR^Q108R^, and CsSGR^Δ37^ proteins.** (**a**) Visualization of the leaves of 4-week-old *N. benthamiana* at 48 h post infiltration with Agrobacterium containing constructs of *CsSGR, CsSGR^Q108R^, CsSGR^Δ37^*-RFP-HA, or *RFP-HA* (control). (**b**) Immunoblot analysis of CsSGR, CsSGR^Q108R^, and CsSGR^Δ37^-RFP-HA.

**
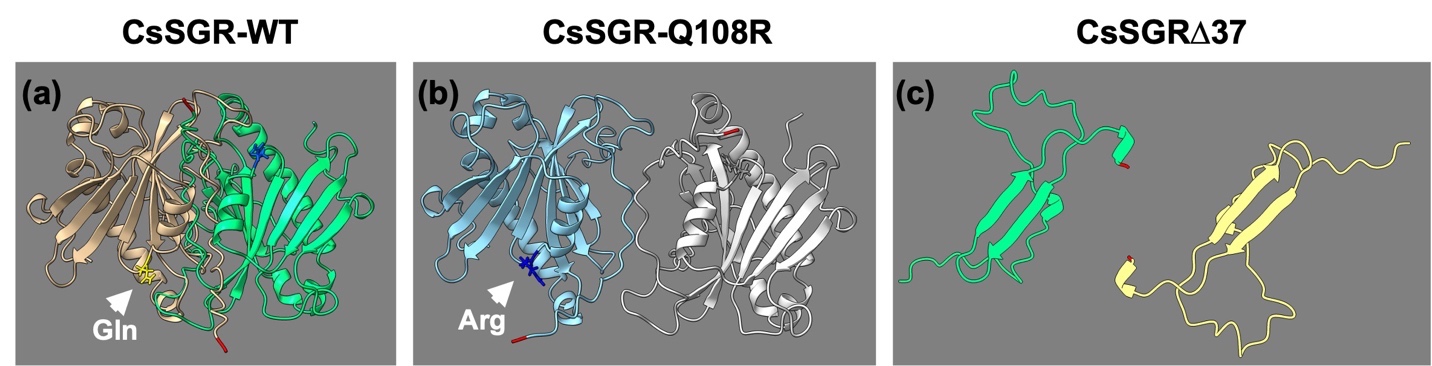
**

**Fig. S26 Predicted protein structures encoded by three *CsSGR* alleles with AlphaFold program.** (**a**) *CsSGR-*WT; (**b**) *CsSGR^Q108R^*; (**c**) *CsSGRΔ37*. The red color tail indicates the C-terminal of the protein.

**
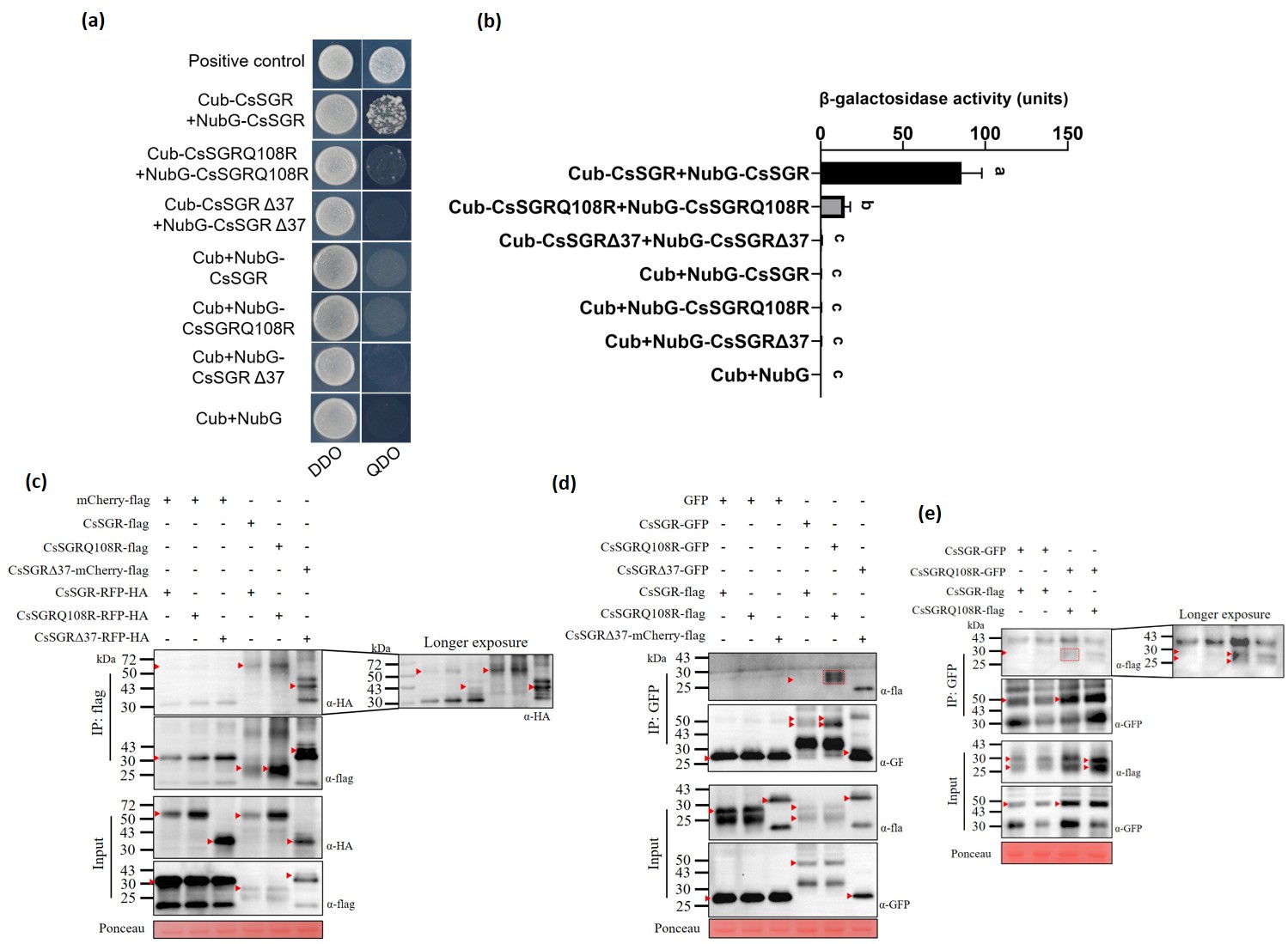
**

**Fig. S27 Mutations in *CsSGR* impair its self-interaction.** (**a**) Y2H assays of self-interactions of CsSGR^WT^, CsSGR^Q108R^, and CsSGR^Δ37^. (**b**) Quantitation of self-interactions of CsSGR, CsSGR^Q108R^, and CsSGR^Δ37^ in yeast β-galactosidase activity assays. Each value = mean ±SD (n=3). Means with different letters are significantly different based on Tukey’s tests. (**c-e**) Co-immunoprecipitation analysis of self-interactions of CsSGR^WT^, CsSGR^Q108R^, and CsSGR^Δ37^ *in vivo*. Anti-FLAG (**c**) or Anti-GFP (**d-e**) beads were used for immunoprecipitation.


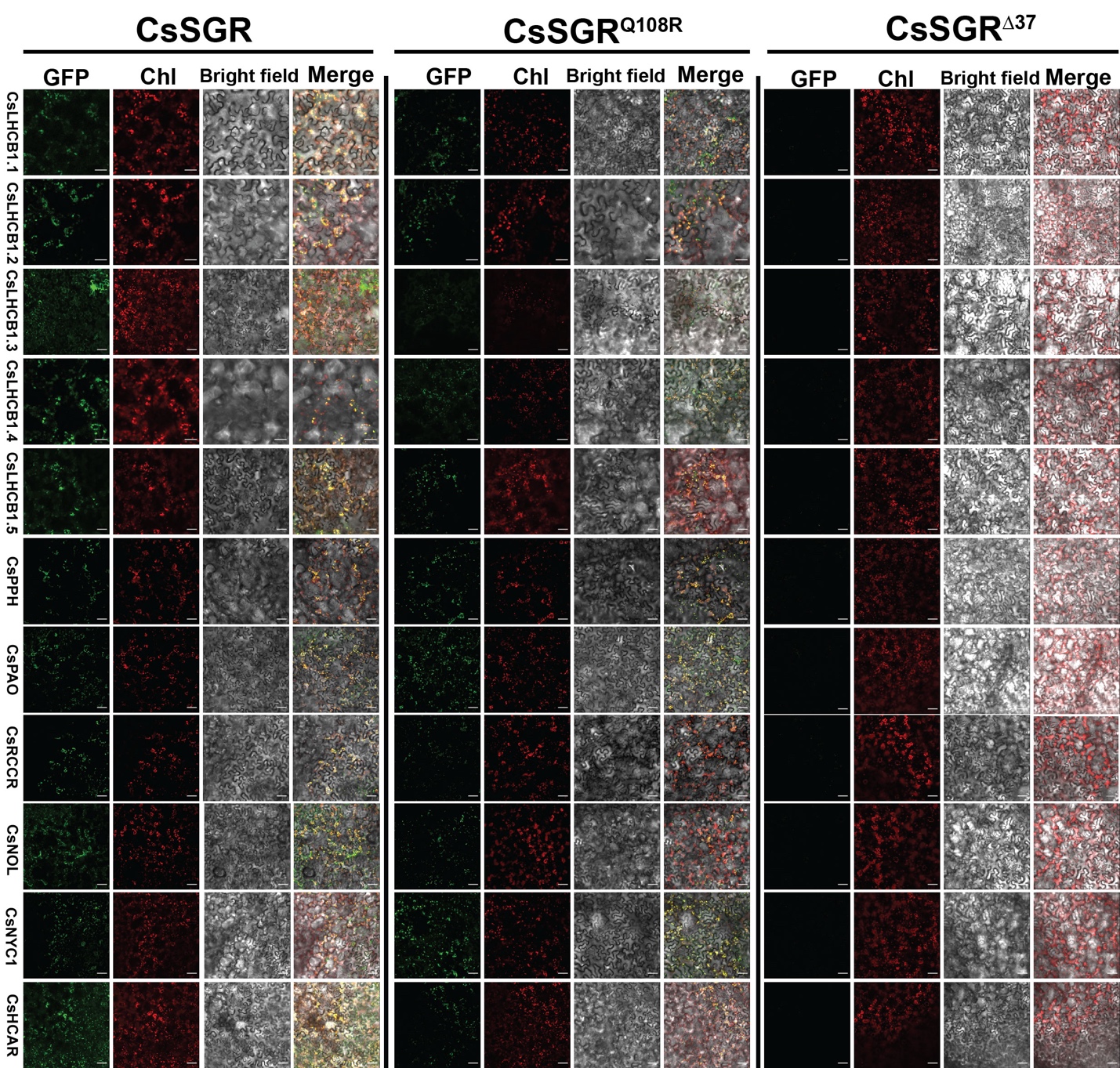


**Fig. S28 BiFC assays of interactions between CsSGR^WT^, CsSGR^Q108R^, or CsSGR^Δ37^ with CsLHCB1s or CsCCEs in the epidermal cells of *N. benthamiana*.** GFP, GFP fluorescence; Chlorophyll, chlorophyll autofluorescence; Bright field, white light; Merge, overlay of all three channels. Scale bar represents 50 μm.

**
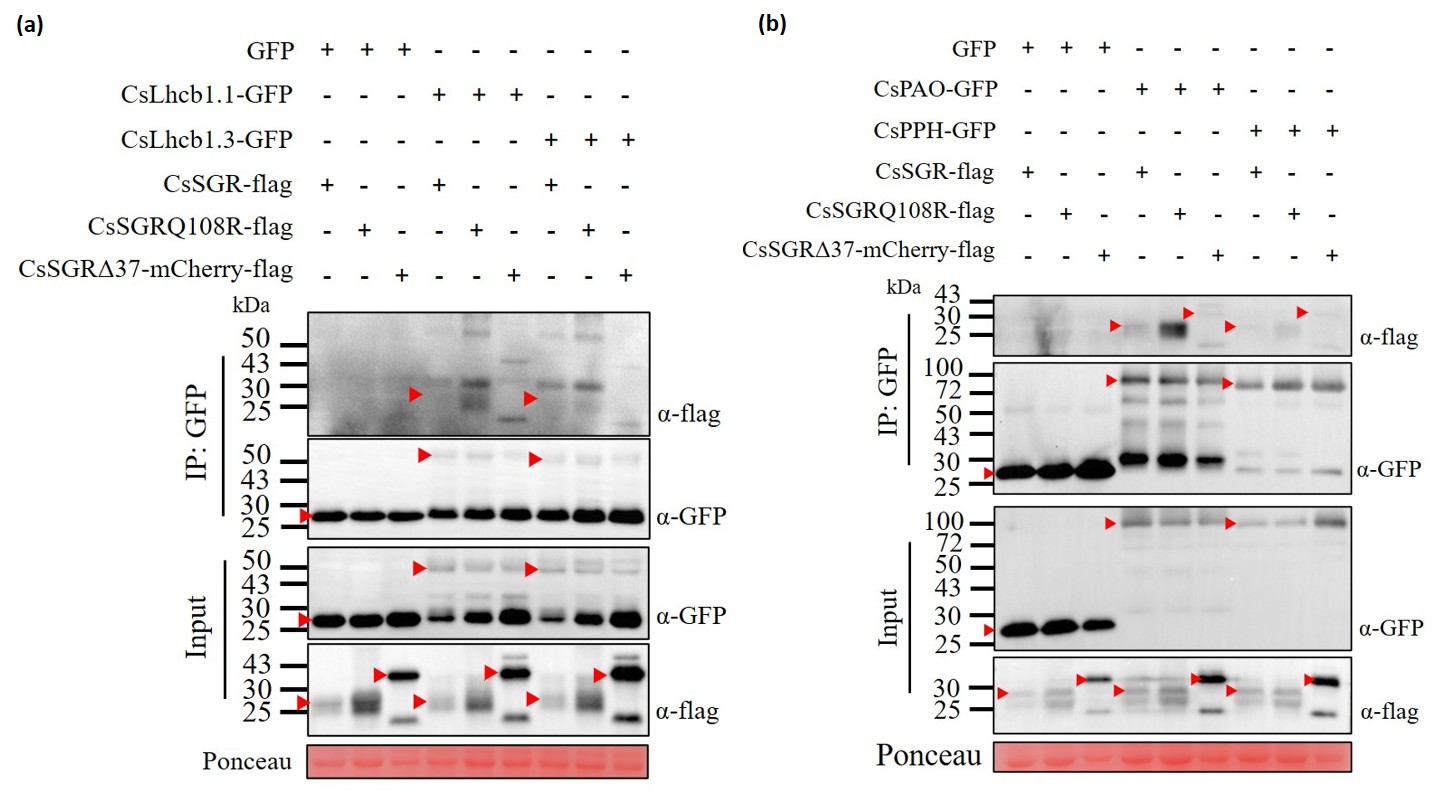
**

**Fig. S29 Co-IP analysis of the interactions of CsSGR^WT^, CsSGR^Q108R^ or CsSGR^Δ37^ with LHCB1.1 (a), LHCB1.3 (a), PPH (b), or PAO (b).** Anti-GFP beads were used for immunoprecipitation.
